# Selective P450_BM3_ Hydroxylation of Cyclobutylamine and Bicyclo[1.1.1]pentylamine Derivatives: Underpinning Synthetic Chemistry for Drug Discovery

**DOI:** 10.1021/jacs.3c10542

**Published:** 2023-12-05

**Authors:** Lucy A. Harwood, Ziyue Xiong, Kirsten E. Christensen, Ruiyao Wang, Luet L. Wong, Jeremy Robertson

**Affiliations:** † Chemistry Research Laboratory, Department of Chemistry, University of Oxford, Mansfield Road, Oxford OX1 3TA, U.K.; ‡ Oxford Suzhou Centre for Advanced Research, Ruo Shui Road, Suzhou Industrial Park, Suzhou, Jiangsu 215123, P. R. China; § Wisdom Lake Academy of Pharmacy, Xi’an Jiaotong-Liverpool University, Suzhou Industrial Park, Suzhou, Jiangsu, 215123, P. R. China; ∥ Inorganic Chemistry Laboratory, Department of Chemistry, University of Oxford, South Parks Road, Oxford OX1 3QR, U.K.

## Abstract

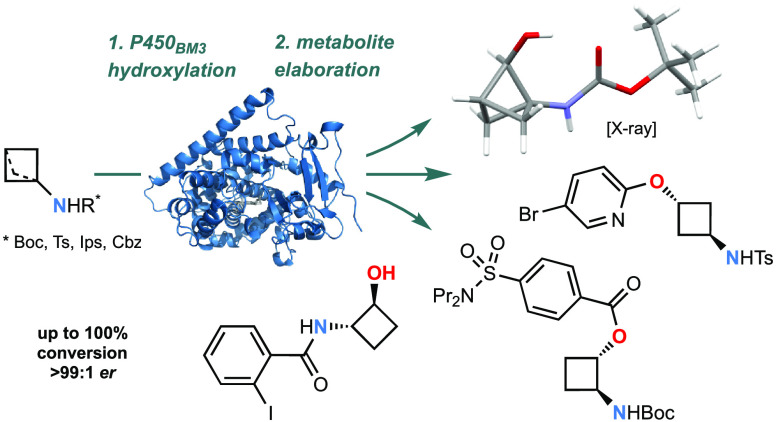

Achieving single-step syntheses of a set of related compounds divergently and selectively from a common starting material affords substantial efficiency gains when compared with preparing those same compounds by multiple individual syntheses. In order for this approach to be realized, complementary reagent systems must be available; here, a panel of engineered P450_BM3_ enzymes is shown to fulfill this remit in the selective C–H hydroxylation of cyclobutylamine derivatives at chemically unactivated sites. The oxidations can proceed with high regioselectivity and stereoselectivity, producing valuable bifunctional intermediates for synthesis and applications in fragment-based drug discovery. The process also applies to bicyclo[1.1.1]pentyl (BCP) amine derivatives to achieve the first direct enantioselective functionalization of the bridging methylenes and open a short and efficient route to chiral BCP bioisosteres for medicinal chemistry. The combination of substrate, enzyme, and reaction engineering provides a powerful general platform for small-molecule elaboration and diversification.

## Introduction

**Figure 1:**
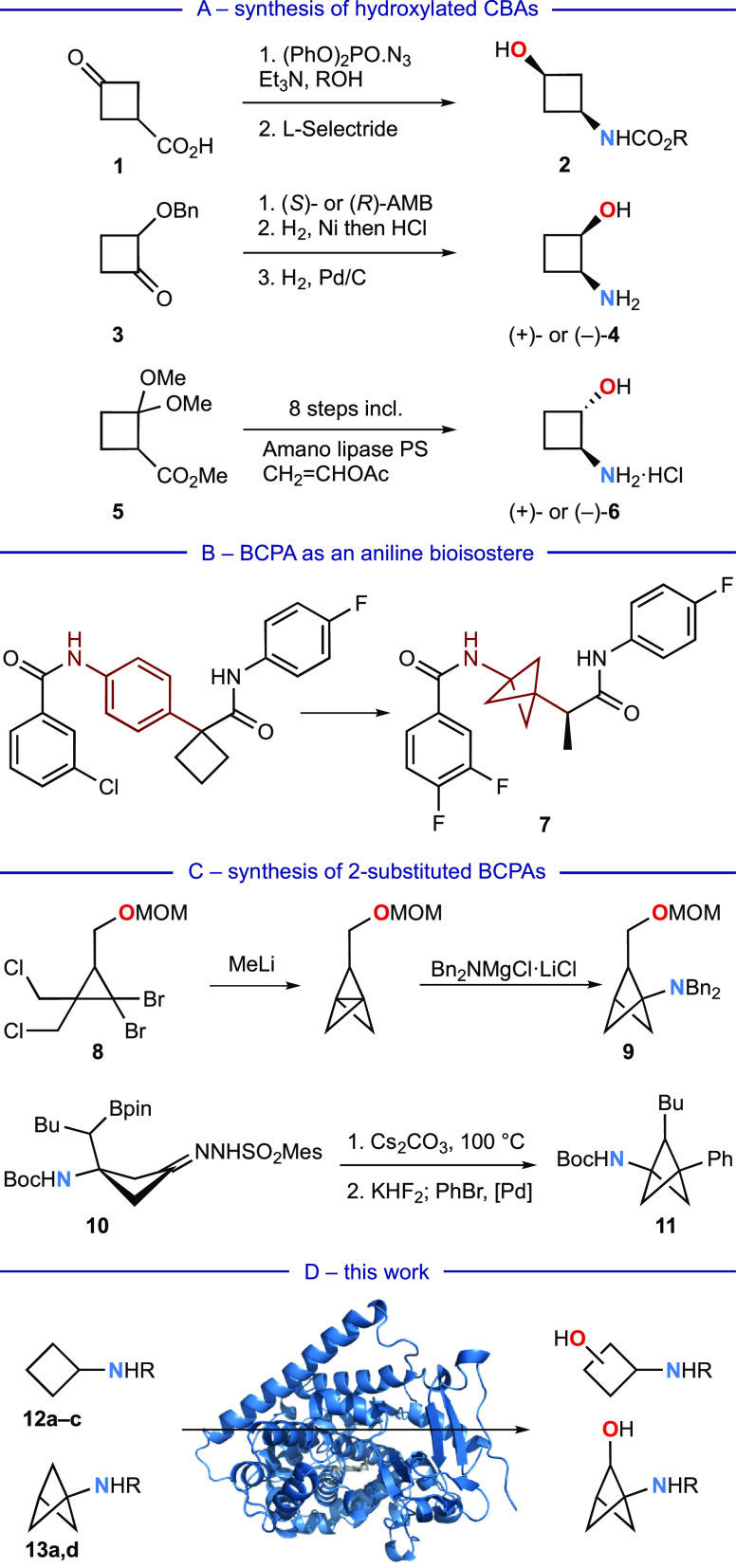
(A) Representative routes to 2- and 3-hydroxylated cyclobutylamines (CBAs); AMB = α-methylbenzylamine. (B) Replacing an aniline linking group in IDO1 inhibitor **6** with bicyclo[1.1.1]pentylamine (BCPA) leads to improved hydrolytic stability and a more favorable profile overall in **7**. (C) Recent routes to C2-functionalized BCPAs. (D) One-step access to CBA- and BCPA-alcohols using P450_BM3_ C–H hydroxylation. For **12** and **13**, R = Boc (**a**), Ts (**b**), Ips (**c**), and Cbz (**d**).

Cyclobutyl amino alcohols are versatile synthetic intermediates whose derivatives feature in medicinal chemistry as key components in pharmaceutical candidates,^1^ as motifs for exploring QSAR models and in lead/fragment-based drug discovery,^2^ and as sp^3^-rich bioisosteric replacements for their aryl counterparts.^3^ Despite this, there are few general methods for producing such compounds, particularly in an enantiomerically enriched form. Usually, each ring size and regio- or stereoisomer requires its own bespoke synthetic route. Representative multistep sequences leading to *cis*-3-hydroxy-cyclobutylamine (CBA) derivatives **2** and the enantiomers of both *cis-***4** and *trans-*2-hydroxy-CBA **6** are summarized in Figure 1A.^4−7^

Bicyclo[1.1.1]pent-1-ylamine (BCPA), a more globular CBA rigidified by virtue of an additional 1,3-methano bridge, presents defined potential exit vectors extending three-dimensionally.^8^ BCPA itself is incorporated into pharmaceutical candidates as an aniline bioisostere, and the readily accessible 3-substituted BCPAs act as bioisosteric replacements for *para-*substituted aniline linkages (e.g., **7**, Figure 1B).^9^ Chiral 3-substituted BCPAs have been prepared in enantioenriched form, but in these the BCP core is stereochemically inert, offering little beyond conformational rigidity and functional group separation.^10^ The 2-disubstituted BCPAs are far more compelling since they are inherently chiral and, as potential bioisosteres for *ortho-* and *meta-*substituted anilines, are desirable targets; however, their synthesis is challenging, particularly in enantioenriched form.^11^ This challenge has been met in part by the groups of Ma^12^ and Baran^13^ building on strain-release amination concepts (**8** → **9**, Figure 1C).^14^ The approach reported by Qin’s group, comprising the cyclization of sulfonyl hydrazones,^15^ the multistep route from Mykhailiuk’s group,^16^ and the elaboration of 2-bromo-BCP-1-carboxylic acid derivatives from MacMillan’s group^17^ all have potential in this context.

While impressive, these high-profile recent advances in CBA and BCPA chemistry have not yet led to a unified synthetic strategy that delivers access to all positions around these cores. Such a strategy would be extremely valuable to both academia and industry, and this report describes developments toward a solution based on selective P450_BM3_-biocatalytic hydroxylation of CBA and BCPA cores (Figure 1D). The approach achieves structural and functional small-molecule diversification via unique oxidized metabolites which connect with the vast chemistry of the hydroxyl and carbonyl groups, circumventing the requirement for tailored syntheses of specific target compounds.

Herein, we highlight the capacity of a P450_BM3_ library to catalyze selective hydroxylation of CBA and BCPA cores via a progressive screening approach, without the need for multiple rounds of mutagenesis and rescreening, testing the extent to which, collectively, these enzymes may be considered off-the-shelf reagents for selective C–H oxidation. The enzyme library evolved from four parent mutants of the P450_BM3_ wild-type, through studies on the oxidation of a variety of substrate classes as described in earlier publications.^18^ Substrates were screened against the same 48-member subset of the wider library (with minor variations, see Supporting Information section S2), selected on the basis of reactivity profiles established in previous work with substrates of similar molecular weights and structural motifs, including anilides, cyclic amines, and cycloalkanes.^19^

In selected cases, focused second-generation panels were constructed from existing variants within the full library based on the metabolite profiles of the initial 48-variant library, and these were subsequently screened against substrates to explore further improvements in selectivity.

## Results and Discussion

Initial analytical screens were conducted for substrate conversion and product selectivity, as judged by gas chromatographic (GC) analysis of the crude organic extract from reactions in 24-well plates. Promising reactions were scaled up sufficiently for NMR characterization of the metabolites and for analytical assays to be established. Free amines are not well tolerated in P450_BM3_ reactions; therefore, the *tert-*butyloxycarbonyl (Boc) derivative **12a** of cyclobutylamine (Figure 1D) was chosen for initial screening. The Boc group imparts a steric and electronic basis for achieving orientation and binding within the active site and is easy to remove when it is no longer required. The main focus of the study remained with Boc-CBA but further N-substituted analogues were screened for comparison.

**Figure 2:**
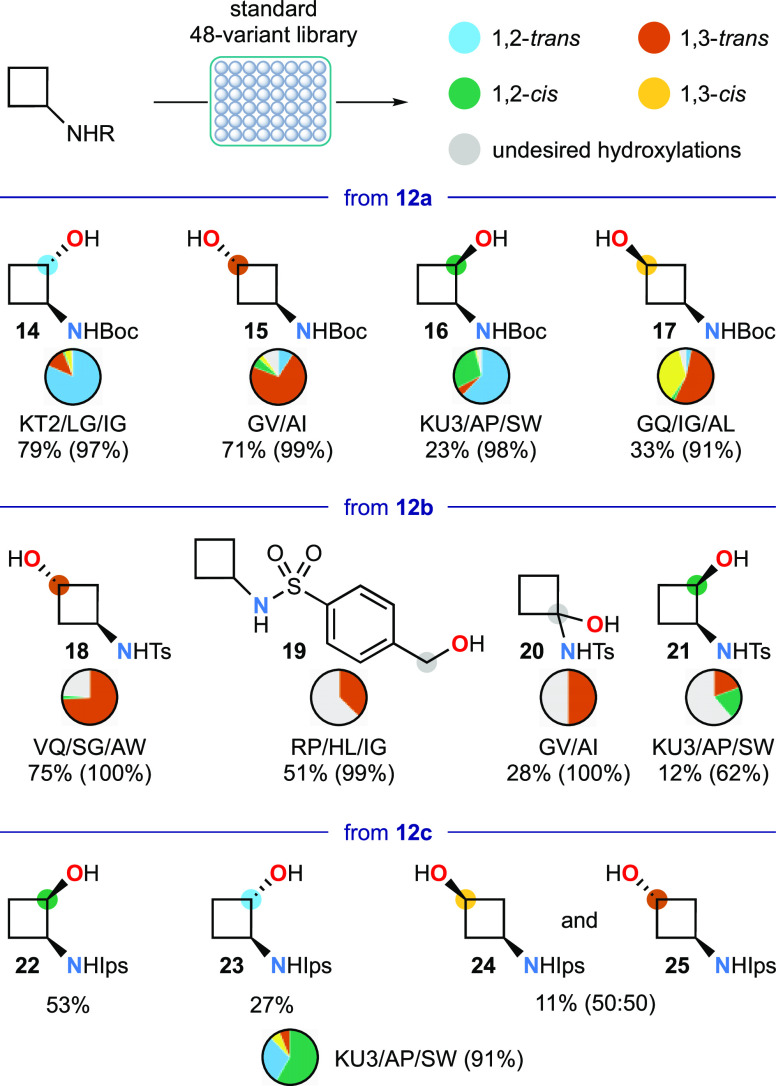
Summary of the screening results for biocatalytic hydroxylation of CBA substrates **12a**–**c** against panels of 48 P450_BM3_ variants. For each substrate, the metabolites are presented in order (left to right) of the most- to least-commonly produced across the panel. Of the P450_BM3_ variants converting ≥30% of the substrate, the most productive variant for each metabolite is given, with the selectivity (%) for that product and substrate conversion (%) in parentheses. Pie charts convey the distribution of metabolites for each variant. Structures for chiral metabolites represent relative configuration. Product **20** was not isolated but was inferred from the observed TsNH_2_ (GC).

The wild-type (WT) P450_BM3_ showed no conversion in the initial screen, but 36 of the variants converted at least 30% of substrate **12a** to products within 24 h. All four 2- and 3-monohydroxylated metabolites were observed (Figure 2), of which the *trans-*2- and *trans-*3-hydroxylated products **14** and **15**, respectively, dominated in most cases. The *cis-*1,2 isomer **16** was the major product for just one of the significantly converting enzymes (RK/AL) although this metabolite was produced more efficiently with KU3/AP/SW despite isomer **14** being the major product with this variant. The *cis-*1,3 isomer **17** was not found as the major product during the screen; the most favorable outcome was with GQ/IG/AL which achieved 33% **17** and 49% **15** at 91% conversion. Minor unidentified metabolites comprised no more than ∼15% of the product mixtures for the high-converting (≥70%) variants. Any α-oxidation product is expected to degrade to cyclobutanone by eliminating *tert-*butyl carbamate and was not observed. The screening program revealed that, collectively, the 48-enzyme subset exhibited a roughly 7:1 preference for hydroxylation *trans* to the NHBoc substituent, with a slight preference for 2- over 3-hydroxylation.

With access to the less-favored *cis-*hydroxylated metabolites in mind, and as a simple alternative to conducting rounds of mutagenesis, the N-substituent was varied in a substrate engineering approach.^20^ The *p-*toluenesulfonyl (Ts) analogue **12b** was also well tolerated by the screening panel, with 31 variants achieving at least 30% conversion of the substrate. Only *trans*-1,3 **18** and *cis-*1,2 **21** cyclobutane hydroxylation products were identified, although *p-*toluenesulfonamide was observed in the GC traces, comprising up to almost 30% of the metabolite integration, consistent with production of the unstable 1-hydroxylation product **20**. 3-Hydroxylation dominated, with 2-hydroxylation occurring to a comparable extent in just two cases. The methyl substituent in the Ts group was susceptible toward hydroxylation, and the derived benzyl alcohol **19** was observed in many screening reactions, accounting for up to 68% of the metabolite mixture and being the second most abundant metabolite overall.

The isopropanesulfonyl (Ips) derivative **12c** showed limited reactivity toward the screening panel with only three variants converting at least 30% of the substrate. Variant KU3/AP/SW gave 91% conversion to all four monohydroxylated products, with the *cis-*1,2 isomer **22** being the major product; notably, this enzyme had also been the most productive for *cis-*1,2 metabolites **16** and **21** from substrates **12a** and **12b**, respectively.

From this initial screening round, changes in the N-substituent were sufficient to favor different major hydroxylated CBA isomers: *trans-*1,2 from **12a**, *trans-*1,3 from **12b**, and *cis-*1,2 from **12c**. The most productive and selective combinations of N-substituent and enzyme variant for each isomer are summarized in Table 1.

**Table 1:** Optimum Combinations of N-Substituent and Enzyme Variant which Achieve Particular Hydroxylation Outcomes from Substrates **12a**–**c** during the Initial Screening Based on Either Production or Selectivity (GC)

	production	selectivity
*trans*-1,2	Boc	Boc
	KT2/LG/IG	RK/AG
*cis*-1,2	Ips	Ips
	KU3/AP/SW	KU3/AP/SW
*trans*-1,3	Ts	Boc or Ts
	VQ/SG/AW	GVQ/IG/AL
*cis*-1,3	Boc	Boc
	GQ/IG/AL	GQ/IG/AL

The chiral *trans-*1,2 metabolite **14** was chosen as an exemplar for optimization, aiming to maximize the enantioselectivity on a synthetically meaningful scale. To begin, variant RP/HL/IG was selected from the highest-converting enzymes in the initial screen because it was the most selective for **14**, it gave the product in 98% ee, and there was an existing panel available of 41 further enzyme variants derived from this starting point. From these second-generation variants, the L437 insertion mutants were found to be the most effective in terms of conversion and selectivity for the production of metabolite **14**. Of these, the RG/LLV variant was more readily expressed in *E. coli* and showed excellent scaleability, with the TTN exceeding 5000 at a substrate/enzyme ratio of 20 000:1 (substrate concentration = 10 mM/1.7 g L^–1^). For preparative scale reactions, a higher substrate concentration achieved a more manageable reaction volume but at the expense of conversion; however, oxygenation of the reaction mixture restored much of the original reactivity. Thus, when either air or oxygen were bubbled through reaction mixtures containing 1.0 mmol of substrate **12a**, conversion progressed approximately four times (air) to six times (oxygen) further during the first six hours of reaction; after this time, the conversions began to plateau, leading to a roughly 2.5-fold (air) and 3.5-fold (oxygen) relative progression at 12 h [see section S5.3.1].

Under these conditions, a 3.42 g (20 mmol) scale reaction (2.0 L) reached 85% conversion at 40 h leading to a 48% isolated yield of (1*S*,2*S*)-**14** (>99% ee), the absolute configuration here being established by correlation with the single crystal X-ray structure of the product from a reaction with the RP/HL/IG/AI variant. From a separate reaction (1.0 mmol of **12a**), sufficient of the minor *cis-*1,2 metabolite **16** (93% ee) was obtained to allow assignment of its absolute configuration as (1*R*,2*S*)- by chemical correlation [see section S5.5]. Repeated screening reactions of substrate **12a** with selected mutants established the reproducibility of the hydroxylation. With five representative enzymes, the absolute values of the conversion, selectivity, and ee varied by ∼1–5%, ∼1–8%, and <1%, respectively [see section S3.3].

For **12b**, second-round screening using a sublibrary of variants designed to increase the steric demand of active site residues by including larger amino acids such as tryptophan, showed variants RT2/FW and RT2/AW to be particularly reactive and selective for the *trans-*1,3 metabolite **18** with few other side-products generated. Reactions with RT2/AW scaled up more successfully, and from 0.1 to 0.6 mmol of substrate **12b**, product **18** was isolated in 46–65% yield. From these reactions, a sample of the minor *cis-*1,2 metabolite **21** was obtained, assigned as (1*S*,2*R*)- from the single crystal diffraction study, complementary to (1*R*,2*S*)-**16** obtained from **12a**.

Substrate **12c** was sufficiently soluble in the aqueous buffer used in the hydroxylation reactions that an organic cosolvent was unnecessary, and this allowed the detrimental effect of typical cosolvents on reactivity to be highlighted. With variant KU3/AP/SW, conversion of substrate **12c** (0.1 mmol) after 48 h was approximately two to three times higher with **12c** added neat than when the substrate was added as a solution in DMSO or ethanol [see section S5.3.2]. With this modification, a preparative-scale reaction showed 88% selectivity for the 2-position and the *cis***22** and *trans***23** isomers were isolated in 22% and 26% yield, respectively. The assigned (1*S*,2*R*)- absolute configuration of the *cis* isomer **22** was supported by Mosher’s ester analysis [see section S6.4]. The improved efficiency of this reaction enabled the minor 3-hydroxylated metabolites to be isolated as an unseparated mixture of the *cis***24** and *trans***25** isomers (8.5%, 60:40 ratio).

**Figure 3:**
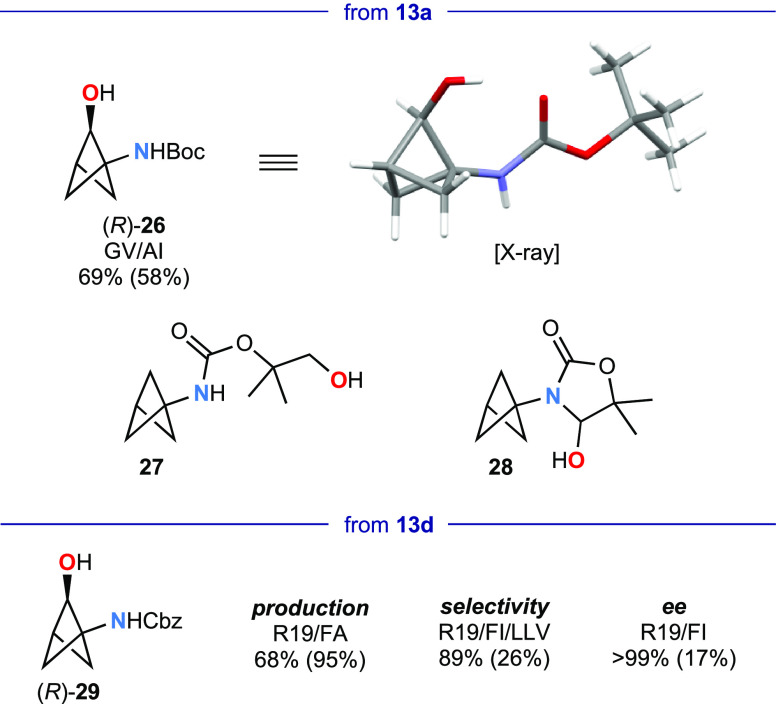
Selected screening results for biocatalytic hydroxylation of BCPA substrates **13a**,**d** against panels of 48 P450_BM3_ variants. The most productive variant for (*R*)-**26** is given, with the selectivity (%) for that product and substrate conversion (%) in parentheses. For metabolite (*R*)-**29**, the enzyme variant providing the highest numerical value of each parameter is given with substrate conversion in parentheses.

Turning to the BCPA series, of the two potential oxidation sites in the BCP ring the high strength of the bridgehead methine C–H bond (BDE ∼110 kcal mol^–1^)^21^ likely precludes biocatalytic hydroxylation at that site; accordingly, interest focused on achieving enantioselective hydroxylation of one of the methylene bridging groups. *N*-Boc BCPA **13a** showed modest reactivity during screening, with the most efficient variant GV/AI achieving 58% conversion, of which 69% was the desired 2-hydroxylation product **26** (Figure 3), with 66% ee in favor of the (2*R*)-enantiomer. The reaction scaled well to produce (2*R*)-**26** in 44% isolated yield from 1.5 mmol of substrate **13a** and recrystallization led to a sample in >99% ee from which the absolute configuration was established from single crystal X-ray diffraction studies [see sections S6.5 and S8]. Just 10 of the other enzyme variants gave 20% or greater conversion, in each case affording **26** with enantiomeric excess ranging from 77% (2*R*)- to 47% (2*S*)- although higher values (up to 82%-*R* and 58%-*S*) were found for some of the less reactive enzyme variants. In this substrate, oxidation of the Boc group was observed, giving hydroxymethyl compound **27** and cyclic hemiaminal **28** [crystallographic data, section S8], usually as minor products in about a quarter of the variants.

The screening panel had captured a wide range of reactivity and enantioselectivity, and preliminary attempts to improve these parameters by rational mutagenesis around key residues (data not shown) were not productive. The *tert-*butyl substituent of the Boc group is roughly isosteric with the BCP core; therefore, the termini of substrate **13a** are poorly differentiated and additionally may limit access of the central heteroatom functionality to the active site residues, both factors likely to negatively impact conversion and selectivity. In view of this, the benzyloxycarbonyl (Cbz) analogue **13d** was screened, and this was found to be significantly more active than **13a**, with 20 of the enzyme variants achieving at least 80% conversion to products. The 2-hydroxylation product **29** dominated over all other identifiable metabolites for over one-third of the enzymes and enantiomeric excesses >90% were achieved in nine cases. The major enantiomer of this metabolite was in the same series throughout, shown to be 2*R*- by comparison of its chiral GC data with a sample prepared from (2*R*)-**26** by Boc deprotection and formation of the Cbz derivative [see section S5.5]. No correlation of the ee values for **26** and **29** with the enzyme variants could be discerned across the two series; for example, variants that delivered **29** in ∼90% or greater ee gave ees for **26** ranging from −50 to +50%, and those giving racemic **29** gave **26** in ees up to 82%. The hydroxylation with variant K19/FV/QP was scaled up to prepare 74 mg of alcohol **29** from 1.4 mmol of substrate **13d** [39% based on recovered **13d** (42%)] from which side-products were observed by NMR spectroscopy that had not been discernible using the GC parameters employed during screening. These included benzyl carbamate and (2-hydroxymethyl)phenol, the latter likely arising from sequential aryl and benzylic hydroxylation and then reduction of the so-formed salicylaldehyde.

**Scheme 1:**
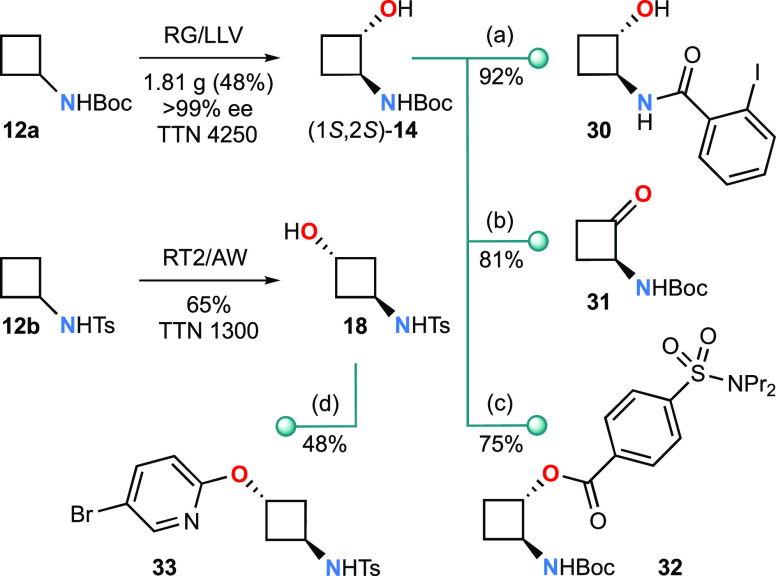
Metabolite Elaboration Reagents and conditions: (a) (i) HCl, aq MeOH, rt, 2 h; (ii) 2-iodobenzoic acid, HATU, *i*-Pr_2_NEt, CH_3_CN, rt, 16 h; (b) Dess–Martin periodinane, CH_2_Cl_2_, rt, 20 h; (c) probenicid, DCC, DMAP, CH_2_Cl_2_, rt, 14 h; (d) NaH, 5-bromo-2-fluoropyridine, DMF, rt, 6 h then 90 °C, 18 h.

Direct hydroxylation of CBA and BCPA cores provides difunctionalized templates from which selective transformations of either the protected amine or the free alcohol functionality can lead rapidly to diverse small-molecule collections.^22^ As summarized in Scheme 1, the newly introduced hydroxyl group in (1*S*,2*S*)-**14** is compatible with removal of the Boc group and N-acylation to give, in this case, a substrate **30** for potential elaboration by cross-coupling chemistry. Conversely, the NHBoc substituent is compatible with oxidation to ketone **31**, under conditions that result in minimal loss of enantiopurity (98% ee).

Selective O-functionalization is illustrated for both (1*S*,2*S*)-**14** and **18** in two medicinal-chemistry relevant applications. First, the 2-aminocyclobutyl ester derivative **32** of probenecid, an antihyperuricemic drug, was obtained by Steglich esterification. Second, the 5-bromo regioisomer **33** of a key intermediate in Amgen’s synthesis of candidate PDE10 inhibitors was prepared by S_N_Ar reaction as a single diastereomer; this compares favorably with the published route, which starts with the considerably more expensive 3-oxocyclobutane carboxylic acid and gives a mixture of diastereomers of the 3-bromo regioisomer in 5% overall yield.^23^

## Conclusion

In combination with varying the N-substituent, the chosen enzymes, representing ∼10% of the larger enzyme library, achieved selective hydroxylation at the 2- and 3-positions in CBA with all diastereomers being accessible. In the absence of targeted mutagenesis campaigns, almost quantitative substrate conversion and 70–80% selectivity were achieved for the *trans* diastereomers, and high enantioselectivity was found for the 1,2-regioisomers. With BCPA carbamates, reliable hydroxylation at the bridging methylenes generated products in both enantiomeric series with ee values spanning the range −58% [(2*S*)-**26**] to >99% [(2*R*)-**29**]. Accordingly, this study confirms that when combined with trivial substrate modifications, a highly focused subset of the existing P450_BM3_ library can achieve strategic C–H hydroxylation to provide high value chiral product molecules. The selectivity of high-performing variants for each product can be enhanced by combining impactful mutations from screening and site-saturation mutagenesis. In principle, further screening could identify variants capable of transforming the first-round metabolites and derivatives leading to trisubstituted small ring amines.

## Supporting Information Available

Experimental procedures and data, screening data, mutations in each enzyme variant described, determination of absolute configuration, and NMR and X-ray crystallographic data (PDF)
Conversion data of variants (XLSX)

## Abbreviations

BCP: bicyclo[1.1.1]pentane
BCPA: bicyclo[1.1.1]pentylamine
CBA: cyclobutylamine
DCC: *N*,*N*′-dicyclohexylcarbodiimide
DMAP: 4-dimethylaminopyridine
DMF: dimethylformamide
HATU: hexafluorophosphate azabenzotriazole tetramethyl uronium
WT: wild-type
