# Supplementary material for: Selective P450BM3 Hydroxylation of Cyclobutylamine and Bicyclo[1.1.1]pentylamine Derivatives: Underpinning Synthetic Chemistry for Drug Discovery
Source: J Am Chem Soc. 2023 Dec 5;145(50):27767–73. doi: 10.1021/jacs.3c10542 (PMC10740007; doi:10.1021/jacs.3c10542)
Supplement: Supplementary file 1 — ja3c10542_si_001.pdf [file ja3c10542_si_001.pdf]

Selective P450$_{BM3}$ Hydroxylation of Cyclobutylamine and Bicyclo[1.1.1]pentylamine Derivatives: Underpinning Synthetic Chemistry for Drug Discovery

Supplementary Information

Lucy A. Harwood[a], Ziyue Xiong[b], Kirsten E. Christensen[a], Ruiyao Wang[c], Luet L. Wong[b,d]* and Jeremy Robertson[a,b]*

[a] Department of Chemistry
University of Oxford
Chemistry Research Laboratory
Mansfield Road, Oxford OX1 3TA, UK.

[b] Oxford Suzhou Centre for Advanced Research
Building A, 388 Ruo Shui Road, Suzhou Industrial Park, Jiangsu, 215123, P. R. China.

[c] Wisdom Lake Academy of Pharmacy
Xi'an Jiaotong-Liverpool University, Suzhou Industrial Park, Jiangsu, 215123, P. R. China.

[d] Department of Chemistry
University of Oxford
Inorganic Chemistry Laboratory
South Parks Road, Oxford OX1 3QR, UK.

Table of Contents

**S1**	General procedures	S3
**S1.1**	Materials and methods	S3
**S1.2**	Gas chromatographic analysis	S4
**S1.3**	Enzymes and molecular biology	S4
**S2**	Lists of variants and mutations	S5
**S3**	Analytical scale library screening	S10
**S3.1**	Protocols	S10
**S3.2**	Tabulated screening data	S10
**S3.3**	Repeats and titer tests	S21
**S4**	Selected GC traces	S23
**S4.1**	GC traces of screening reactions	S23
**S4.2**	Chiral GC traces of isolated metabolites	S26
**S5**	Synthetic chemistry	S32
**S5.1**	Synthesis of substrates	S32
**S5.2**	Semi-preparative hydroxylations for metabolite characterization	S35
**S5.3**	Reaction optimization and preparative-scale hydroxylations	S42
**S5.3.1**	Effect of aeration during reaction	S42
**S5.3.2**	Effect of substrate stock solvent	S43
**S5.3.3**	Hydroxylation of *tert*-butyl cyclobutylcarbamate **12a** (20 mmol)	S43
**S5.3.4**	Hydroxylation of *tert*-butyl bicyclo[1.1.1]pent-1-ylcarbamate **13a** (1.5 mmol)	S44
**S5.3.5**	Hydroxylation of benzyl bicyclo[1.1.1]pent-1-ylcarbamate **13d** (1.4 mmol)	S44
**S5.4**	Metabolite derivatization	S45
**S5.5**	Synthesis of racemic samples and other compounds used for establishing *ee*	S48
**S6**	Determination of stereochemical configuration	S50
**S6.1**	NOESY correlations for **14** and **16**	S50
**S6.2**	Absolute configuration of (1*R*,2*S*)-**16**	S51
**S6.3**	Absolute configuration of (*R*)-**29**	S52
**S6.4**	Absolute configuration of (1*S*,2*R*)-**22**	S53
**S6.5**	Chiral GC data to accompany crystallographic data	S54
**S7**	NMR spectra	S55
**S8**	Crystallographic data	S78
**S9**	References	S86

S1 General procedures

S1.1 Materials and methods

Solvents and reagents were used as commercially supplied or were purified by standard techniques. Acetonitrile, diethyl ether ('ether'), dichloromethane and benzene were obtained from Grubbs canisters, with the solvent passing through an activated alumina column under argon. THF was freshly distilled over Na/benzophenone. Hünig's base and triethylamine were distilled over KOH and stored under argon. All glassware was dried with a heat-gun before use. All organic synthesis reactions were run under nitrogen or argon atmosphere unless otherwise stated.

Media components, kanamycin and IPTG were supplied by Melford Laboratories, UK; lysozyme was from Sigma-Aldrich, $NADP^+$ was from Prozomix and glucose dehydrogenase (GDH) was from Codexis, USA. Oligonucleotides were supplied by Eurofins Genetic Service, UK. Standard phosphate buffer used as reaction media in biotransformations was prepared from KH_2PO_4 (2.40 g) and K_2HPO_4 (31.7 g) dissolved in deionized water (1.0 L), and the pH adjusted to 7.9 (by addition of small amounts of either phosphate salt) before use.

Merck aluminium-backed DC60 F254 plates (0.2 mm) were used for TLC analysis and were visualized with ultra-violet light before staining with $KMnO_4$, phosphomolybdic acid or vanillin staining solutions and developed with heat. Merck silicagel 60 (43–60 µm) was used for column chromatography and the solvent system used and retention factors are recorded with experimental data.

Proton (^1H) and carbon (^{13}C) NMR spectra were recorded on Bruker AVIII 400 MHz, 500 MHz or 600 MHz spectrometers as specified. Chemical shift (δ_H and δ_C) values are recorded to the nearest 0.01 ppm and 0.1 ppm, respectively; coupling constants have been rounded to the nearest 0.5 Hz. Peak multiplicities are described as apparent (app.), broad (br.), singlet (s), doublet (d), triplet (t), quartet (q), etc., combinations thereof, or multiplets (m). NMR spectra are referenced (in MestReNova) to the appropriate solvent resonance: $CDCl_3$ 7.26/77.16 ppm; DMSO-d_6 2.50/39.52 ppm. Peak assignments were made based on chemical shift, integration, coupling constants, COSY and HSQC spectra. HMBC spectra were obtained in selected cases as necessary. Infra-red (IR) spectra were recorded using a Bruker Tensor 27 FT-IR spectrometer. Absorption maxima are reported in wavenumbers/cm^{-1} and described as strong (s), medium (m), weak (w) and broad (br.) relative to the most intense peak; weak peaks not attributed to a specific functional group are not reported. High resolution mass spectra were obtained for novel compounds on a Bruker Daltronics MicroTOF spectrometer (ESI); mass to charge ratios (*m/z*) are reported in Daltons. Melting points were recorded in degrees Celsius (°C) using a Griffin melting point apparatus. Optical rotation data were acquired on a UniPol 2000 digital polarimeter (Schmidt & Haensch, Germany). Measurements were taken at 25 °C using a wavelength of 589.44 nm. The pathlength of the cell was 1 dm. Specific rotations, $[\alpha]_D^{25}$, were calculated by the formula $[\alpha]_D^{25} = \alpha/(l \times c)$, where α is the average of 10 readings taken from the polarimeter, l is the pathlength in decimetres, and c is the sample concentration in g/100 mL.

S1.2 Gas chromatographic analysis

Gas chromatographic (GC) analyses were carried out with a ThermoFisher Scientific Trace 1300 instrument equipped with a flame ionization detector (FID) and an AI1310 autosampler using a J&W DB-1MS fused silica column (30 m × 0.25 mm × 0.25 μm, Agilent Technology, UK) or for chiral phase analysis a Cyclosil-B fused silica column (30 m × 0.25 mm × 0.25 μm, Agilent Technology, UK), with helium as carrier gas at a flow rate of 1.5 mL min^{-1}. For normal phase analyses, both the injector and the FID were held at 250 °C. For chiral phase analyses, the injector was held at 200 °C and the FID at 250 °C. Substrate conversion and product percentages were determined from integrated peak areas.

For normal-phase GC analysis of the oxidation products of *N*-Boc-cyclobutylamine, **12a**, the oven temperature was held at 90 °C for 3 min then raised at 20 °C/min to 240 °C and held for 1 min (total time = 11.5 min). For normal-phase GC analysis of the oxidation products of *N*-Ts-cyclobutylamine, **12b**, the oven temperature was held at 180 °C for 1 min then raised at 15 °C/min to 300 °C and held for 2 min (total time = 11 min). For normal-phase GC analysis of the oxidation products of *N*-Ips-cyclobutylamine, **12c**, the oven temperature was held at 130 °C for 2 min then raised at 20 °C/min to 270 °C and held for 1 min (total time = 10 min). For normal-phase GC analysis of the oxidation products of *N*-Boc-bicyclo[1.1.1]pentylamine, **13a**, the oven temperature was held at 90 °C for 3 min then raised at 20 °C/min to 240 °C and held for 1 min (total time = 11.5 min). For normal-phase GC analysis of the oxidation products of *N*-Cbz-bicyclo[1.1.1]pentylamine, **13d**, the oven temperature was held at 180 °C for 1 min then raised at 15 °C/min to 300 °C and held for 1 min (total time = 10 min).

S1.3 Enzymes and molecular biology

Genes encoding $P450_{BM3}$ enzymes were cloned in the pET28+ vector by NcoI and BamHI restriction sites.[1] Site-directed mutagenesis was carried out by standard PCR-based protocols using a KOD Hot Start DNA Polymerase kit from Sigma-Aldrich, UK. The presence of the target mutation(s) was confirmed by DNA sequencing. The relevant plasmid was transformed into chemically competent *E. coli* BL21 (DE3) cells for enzyme production and subsequent purification as described previously.[2] P450 content was quantified using the CO-difference method, using UV-vis spectra acquired on a Varian CARY50 spectrophotometer at 30 °C using 1 cm pathlength quartz cuvettes.[3]

S2 Lists of variants and mutations

First generation library enzymes

	Variant	Mutations
1	AP	A330P
2	GLQ/IG/AG	A74G/F87L/L188Q/I263G/A328G
3	GQ/IG/AL	A74G/L188Q/I263G/A328L
4	GV/AI	A74G/F87V/A184I
5	GV/AI/IG/AG	A74G/F87V/A184I/I263G/A328G
6	GVQ	A74G/F87V/L188Q
7	GVQ/A264G	A74G/F87V/L188Q/A264G
8	GVQ/A328G	A74G/F87V/L188Q/A328G
9	GVQ/AW	A74G/F87V/L188Q/A330W
10	GVQ/IG	A74G/F87V/L188Q/I263G
11	GVQ/IG/AL	A74G/F87V/L188Q/I263G/A328L
12	K19/FV	F87V/H171L/Q307H/N319Y
13	K19/FV/AG	F87V/H171L/A264G/Q307H/N319Y
14	K19/FV/EV	F87V/H171L/E267V/Q307H/N319Y
15	K19/FV/EV/VI	V78I/F87V/H171L/E267V/Q307H/N319Y
16	K19/FV/IG	F87V/H171L/I263G/Q307H/N319Y
17	K19/FV/QP	F87V/H171L/Q307H/N319Y/Q403P
18	K19/FA/AM/IG	A82M/F87A/H171L/I263G/Q307H/N319Y
19	K19/FA/FW	F81W/F87A/H171L/Q307H/N319Y
20	K19/FA/IA	F87A/H171L/I263A/Q307H/N319Y
21	K19/FA/IG	F87A/H171L/I263G/Q307H/N319Y
22	KT2/LG/IG	R47L/Y51F/L188G/A191T/N239H/I259V/I263G/A276T/L353I
23	KU3/AP/AI	N239H/I259V/A276T/A328I/A330P
24	KU3/AP/SW	S72W/N239H/I259V/A276T/A330P
25	R19/AL/AL	R47L/Y51F/H171L/A184L/Q307H/N319Y/A328L
26	R19/AL/IA	R47L/Y51F/H171L/I263A/Q307H/N319Y/A328L
27	R19/AW	R47L/Y51F/H171L/Q307H/N319Y/A330W
28	R19/FI	R47L/Y51F/F87I/H171L/Q307H/N319Y
29	R19/FI/LLV	R47L/Y51F/F87I/H171L/Q307H/N319Y/L437LV
30	R19/SG/AW	R47L/Y51F/S72G/H171L/Q307H/N319Y/A330W
31	R19	R47L/Y51F/F87V/H171L/Q307H/N319Y
32	R19/FA	R47lL/Y51F/F87A/H171L/Q307H/N319Y
33	R19/FA/AI	R47L/Y51F/F87A/H171L/A184I/Q307H/N319Y
34	RK/AI/TG/AG	R47L/Y51F/F87A/H171L/A184I/T269G/Q307H/N319Y/A328G
35	RK/AG	R47L/Y51F/F87A/H171L/Q307H/N319Y/A328G
36	RK/AG/AG	R47L/Y51F/F87A/H171L/A264G/Q307H/N319Y/A328G
37	RK/AG/PG/AG	R47L/Y51F/F87A/H171L/Q307H/N319Y/A328G/P329G/A330G
38	RK/AL	R47L/Y51F/F87A/H171L/Q307H/N319Y/A328L

39	RK/FW/TG/AG	R47L/Y51F/F81W/F87A/H171L/T269G/Q307H/N319Y/A328G
40	RK/T260G	R47L/Y51F/F87A/H171L/T260G/Q307H/N319Y
41	RK/T269G	R47L/Y51F/F87A/H171L/T269G/Q307H/N319Y
42	RKA/SW	R47L/Y51F/S72W/F87A/H171L/Q307H/N319Y/A328I
43	RKA/VI	R47L/Y51F/V78I/F87A/H171L/Q307H/N319Y/A328I
44	RP/AM/IA	R47L/Y51F/A82M/I263A/I401P
45	RP/HL/IG	R47L/Y51F/H171L/I263G/I401P
46	RP/HL/IG/A184I	R47L/Y51F/H171L/A184I/I263G/ /I401P
47	RP/IA/EV	R47L/Y51F/I263A/E267V/I401P
48	RT2/SG/AW	R47L/Y51F/S72G/A191T/N239H/I259V/A276T/A330W/L353I
49	VQ/SG/AW	S72G/F87V/L188Q/A330W
50	WT	WT

Second generation library enzymes

	Variant	Mutations
1	GA/AI	A74G/F87A/A184I
2	GG/AI	A74G/F87G/A184I
3	GL/AI	A74G/F87L/A184I
4	GV/AF	A74G/F87V/A184F
5	GV/AI	A74G/F87V/A184I
6	GV/AM	A74G/F87V/A184M
7	GVAI/AL	A74G/F87V/A184I/A328L
8	GVAI/AI	A74G/F87V/A184I/A330I
9	GVAI/AW	A74G/F87V/A184I/A330W
10	GVAI/HL/LQ	A74G/F87V/H171L/A184I/L188Q
11	GVAI/IW	A74G/F87V/A184I/I263W
12	GVAI/LM	A74G/F87V/A184I/L188M
13	GVAI/LQ	A74G/F87V/A184I/L188Q
14	GVAI/LF	A74G/F87V/A184I/L437F
15	GVAI/LLF	A74G/F87V/A184I/L437LF
16	GVAI/LLH	A74G/F87V/A184I/L437LH
17	GVAI/LLM	A74G/F87V/A184I/L437LM
18	GVAI/LM	A74G/F87V/A184I/L437M
19	GVAI/LF	A74G/L75F/F87V/A184I
20	GVAI/SM	S72M/A74G/F87V/A184I
21	GVAI/VF	V26F/A74G/F87V/A184I
22	GVAI/VL	V26L/A74G/F87V/A184I
23	GVAI/VM	V26M/A74G/F87V/A184I
24	GVQ/SW	S72W/A74G/F87V/L188Q
25	HL/IG	H171L/I263G
26	IG	I263G
27	IR	I263R
28	IS	I263S
29	IV	I263V
30	IW	I263W
31	K19/FV/AL	F87V/H171L/Q307H/N319Y/A328L
32	K19/FA/AI	F87A/H171L/Q307H/N319Y/A328I
33	K19/FA/AI/IA	F87A/H171L/I263A/Q307H/N319Y/A328I
34	K19/FA/AM	A82M/F87A/H171L/Q307H/N319Y
35	KU3/AS/LLA	N239H/I259V/A276T/A330S/L437LA
36	R19/AW	R47L/Y51F/H171L/Q307H/N319Y/A330W
37	R19/FI/AP	R47L/Y51F/F87I/H171L/Q307H/N319Y/A330P
38	R19/FI/AW	R47L/Y51F/F87I/H171L/Q307H/N319Y/A330W
39	RL/YF/HL/IR	R47L/Y51F/H171L/I263R

40	RL/YF/HL/IS	R47L/Y51F/H171L/I263S
41	RL/YF/HL/IV	R47L/Y51F/H171L/I263V
42	RL/YF/HL/IW	R47L/Y51F/H171L/I263W
43	RL/YF/VL/HL/IR	R47L/Y51F/V78L/H171L/I263R
44	RL/YF/VL/HL/IS	R47L/Y51F/V78L/H171L/I263S
45	RL/YF/VL/HL/IV	R47L/Y51F/V78L/H171L/I263V
46	RL/YF/VL/HL/IW	R47L/Y51F/V78L/H171L/I263W
47	RG	R47L/Y51F/H171L/I263G
48	RG/AP	R47L/Y51F/H171L/I263G/A330P
49	RG/AM	R47L/Y51F/A82M/H171L/I263G
50	RG/FW	R47L/Y51F/F81W/H171L/I263G
51	RG/K202KPG	R47L/Y51F/H171L/K202KPG/I263G
52	RG/LLA	R47L/Y51F/H171L/I263G/L437LA
53	RG/LLF	R47L/Y51F/H171L/I263G/L437LF
54	RG/LLH	R47L/Y51F/H171L/I263G/L437LH
55	RG/LLM	R47L/Y51F/H171L/I263G/L437LM
56	RG/LLS	R47L/Y51F/H171L/I263G/L437LS
57	RG/LLV	R47L/Y51F/H171L/I263G/L437LV
58	RG/LS	R47L/Y51F/H171L/I263G/L437S
59	RG/LVL	R47L/Y51F/H171L/I263G/L437VL
60	RG/RH	R47L/Y51F/H171L/I263G/R398H
61	RG/VI/LLA	R47L/Y51F/V78I/H171L/I263G/L437LA
62	RG/VI/LLH	R47L/Y51F/V78I/H171L/I263G/L437LH
63	RG/VI/LLI	R47L/Y51F/V78I/H171L/I263G/L437LI
64	RG/VI/LLM	R47L/Y51F/V78I/H171L/I263G/L437LM
65	RG/VI/LLS	R47L/Y51F/V78I/H171L/I263G/L437LS
66	RG/VI/LLV	R47L/Y51F/V78I/H171L/I263G/L437LV
67	RG/VI/LVL	R47L/Y51F/V78I/H171L/I263G/L437VL
68	RG/VL	R47L/Y51F/V78L/H171L/I263G
69	RP/EG	R47L/Y51F/E267G/I401P
70	RP/EV	R47L/Y51F/E267V/I401P
71	RP/FV/EV	R47L/Y51F/F87V/E267V/I401P
72	RP/HL/IG	R47L/Y51F/H171L/I263G/I401P
73	RP/HL/IG/AI	R47L/Y51F/H171L/A184I/I263G/I401P
74	RP/HL/IG/AP	R47L/Y51F/H171L/I263G/A330P/I401P
75	RP/HL/IG/LLV	R47L/Y51F/H171L/I263G/I401P/L437LV
76	RP/HL/IR	R47L/Y51F/H171L/I263R/I401P
77	RT2	R47L/Y51F/A191T/N239H/I259V/A276T/L353I
78	RT2/AN	R47L/Y51F/A191T/N239H/I259V/A276T/A328N/L353I
79	RT2/AW	R47L/Y51F/A191T/N239H/I259V/A276T/A330W/L353I
80	RT2/AP/AI	R47L/Y51F/A184I/A191T/N239H/I259V/A276T/A330P/L353I
81	RT2/AP/AM	R47L/Y51F/A82M/A191T/N239H/I259V/A276T/A330P/L353I

82	RT2/AP/LLA	R47L/Y51F/A191T/N239H/I259V/A276T/A330P/L353I/L437LA
83	RT2/AP/VI	R47L/Y51F/V78I/A191T/N239H/I259V/A276T/A330P/L353I
84	RT2/FW	R47L/Y51F/F81W/A191T/N239H/I259V/A276T/L353I
85	RT2/IP	R47L/Y51F/A191T/N239H/I259V/A276T/L353I/I401P
86	RT2/SG/AP/LLA	R47L/Y51F/S72G/A191T/N239H/I259V/A276T/A330P/L353I/L437LA
87	RT2/SG/AW/LLA	R47L/Y51F/S72G/A191T/N239H/I259V/A276T/A330W/L353I/L437LA
88	SW/FV/AI	S72W/F87V/A184I

S3 Analytical scale library screening

S3.1 Protocols

Reaction conditions: the substrate (DMSO stock solution) and enzyme concentration are stated below for each screen; the cofactor and recycling reagents comprised glucose (50 µL, 1.0 M solution in phosphate buffer), GDH (10 µL, 2.0 U/µL solution in phosphate buffer), $NADP^+$ monosodium salt (10 µL, 4.0 mM solution in buffer), in phosphate buffer (320 µL). Reactions were conducted in 24-well plates shaken at 120 rpm at 20 °C for 24 h after which the content of each well was mixed with ethyl acetate (300 µL), vortexed in 1.5 mL microcentrifuge tubes for 30 s, and then centrifuged at 13,300 *g* for 1 min after which 150 µL of the organic layer was transferred to GC vials for GC analysis. Conversions <1% are denoted as '-'. *Total turnover number* (TTN) is calculated by [1–(peak area of substrate/100)] × (ratio of substrate to enzyme). *Selectivity* is calculated as (ratio of specifed product/all products), and *turnover number* (TON) for a specified product is therefore calculated as TTN × selectivity. TTN and TON values are rounded to the nearest 5.

S3.2 Tabulated screening data

Table S1: First generation library screening data for substrate **12a**, at 1.0 mM substrate concentration and 2.0 µM enzyme concentration (500:1 substrate-to-enzyme ratio), ordered by conversion.

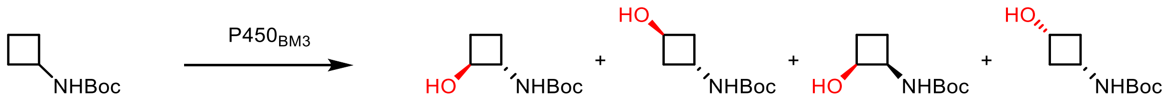

Variant	Conversion /%	14 /%	15 /%	16 /%	17 /%	Other /%	TTN
RP/HL/IG/AI	100	68	20	-	4	8	500
GV/AI	99	9	71	6	3	10	495
RP/HL/IG	99	70	20	2	4	3	495
K19/FV/EV	98	33	47	5	4	9	490
KU3/AP/SW	98	61	5	28	1	3	490
K19/FV/QP	97	16	62	1	4	14	485
KT2/LG/IG	97	79	12	1	4	1	485
R19/FI/LLV	96	54	26	1	5	10	480
VQ/SG/AW	93	26	51	7	5	4	465
GQ/IG/AL	91	3	49	2	33	4	455
R19/FA	90	63	13	-	-	14	450
K19/FV/IG	89	31	45	1	4	8	445
GVQ/AW	86	22	43	4	5	12	430
GVQ/IG	85	35	31	2	5	12	425
K19/FA/IG	83	42	28	2	5	6	415
R19/FI	81	45	23	9	2	2	405
GVQ	78	24	39	3	3	9	390
GVQ/IG/AL	78	2	60	-	6	10	390

K19/FV	71	19	36	7	2	7	355
RK/AG	71	71	-	-	-	-	355
RT2/SG/AW	69	41	2	11	1	14	345
R19/FA/AI	68	34	22	3	4	5	340
RP/AM/IA	64	5	42	8	8	1	320
K19/FA/FW	62	27	6	-	4	25	310
K19/FA/IA	54	13	29	4	2	6	270
RKA/SW	51	16	10	3	9	13	255
RK/AL	50	8	10	22	2	8	250
GV/AI/IG/AG	48	21	15	7	2	3	240
RK/AG/PG/AG	43	37	2	1	-	3	215
RK/FW/TG/AG	41	11	-	1	-	29	205
RP/IA/EV	39	17	12	5	1	4	195
K19/FV/EV/VI	36	22	7	6	1	-	180
GVQ/A328G	34	22	4	6	1	1	170
R19/AW	32	19	-	6	-	7	160
R19/SG/AW	31	21	-	5	-	5	155
K19/FV/AG	30	3	15	6	6	-	150
R19	25	13	2	8	-	2	125
GVQ/A264G	16	2	7	2	4	1	80
GLQ/IG/AG	15	-	11	-	-	4	75
R19/AL/IA	12	-	3	7	-	2	60
RKA/VI	11	3	2	1	3	2	55
RK/AG/AG	8	5	-	1	-	2	40
K19/FA/AM/IG	4	-	-	2	-	2	20
RK/AI/TG/AG	4	2	-	1	-	1	20
RK/TG	4	2	-	-	-	2	20
AP	3	1	1	-	-	1	15
KU3/AP/AI	1	-	-	-	-	1	5
WT	-	-	-	-	-	-	-

Table S2: Second generation library screening data for substrate **12a**, at 4.0 mM substrate concentration and 2.0 µM enzyme concentration (2000:1 substrate-to-enzyme ratio), ordered by conversion.

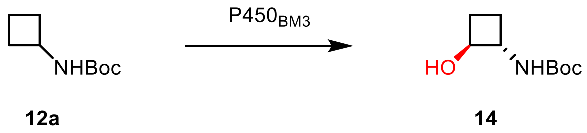

Variant	Conversion /%	14 /%	Other /%	TTN	TON
RG/VI/LVL	70	59	12	1410	1170
RP/HL/IG/LLV	67	56	11	1345	1125
RG	64	31	33	1285	625
RG/LLV	64	51	13	1285	1020
RG/LVL	64	49	15	1290	990
RP/HL/IG/AI	62	44	19	1245	870
RG/FW	58	23	35	1170	460
RG/V78I/L437LV	57	45	12	1145	905
RG/RH	44	20	25	890	395
RG/K202KPG	43	22	21	865	440
RG/LLA	42	32	11	850	630
RG/VI/LLI	41	30	10	810	605
RG/VL	41	27	14	815	540
RG/VI/LLA	39	26	13	785	520
RG/LLF	37	28	10	750	555
RP/HL/IG	37	27	11	745	530
RG/LLH	35	27	8	700	530
IG	32	17	15	635	745
HL/IG	31	15	16	615	295
RP/HL/IG/AP	31	19	11	610	390
RG/AP	30	17	13	605	345
RG/VI/LLH	27	17	10	535	345
RG/LLM	23	14	10	470	275
IS	21	1	20	415	10
RG/VI/LLS	21	13	8	425	255
RG/LLS	16	10	5	315	210
RL/YF/HL/IS	16	4	13	325	70
RG/LS	15	4	11	310	85
RG/VI/LLM	14	9	5	275	180
RG/AM	9	1	7	175	25
RL/YF/VL/HL/IR	7	5	1	130	110
RL/YF/HL/IR	6	5	1	115	100
IR	2	1	-	30	25
RL/YF/HL/IV	2	1	1	50	20
RL/YF/HL/IW	2	1	1	40	25
RL/YF/VL/HL/IV	2	1	1	35	25
RL/YF/VL/HL/IW	1	1	-	20	15
IV	-	-	-	0	-
IW	-	-	-	5	10
RL/YF/VL/HL/IS	-	-	-	0	-
RP/HL/IR	-	-	1	15	-

Table S3: First generation library screening data for substrate **12b**, at 1.0 mM concentration and 2.0 µM enzyme concentration (500:1 substrate-to-enzyme ratio), ordered by conversion.

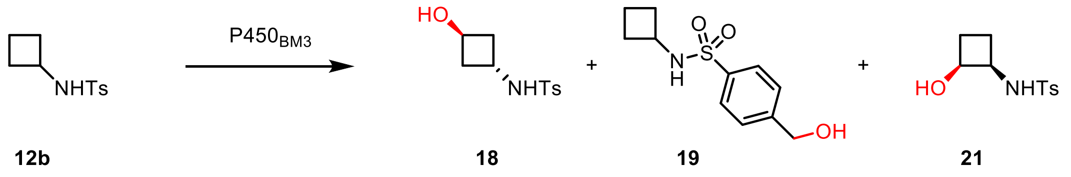

Variant	Conversion /%	18 /%	19 /%	$TsNH_2$ /%	21 /%	Other /%	TTN
GV/AI	100	50	-	28	-	22	500
K19/FV/IG	100	40	31	3	-	27	500
RP/HL/IG/AI	100	57	31	-	-	13	500
VQ/SG/AW	100	75	7	9	2	8	500
KT2/LG/IG	99	47	39	-	-	14	495
RP/HL/IG	99	37	51	-	-	11	495
GVQ/AW	98	68	11	7	-	11	490
GVQ/IG	98	54	19	2	-	23	490
K19/FV/QP	97	52	2	11	5	26	485
GVQ	86	61	7	5	4	10	430
R19/FI/LLV	75	6	51	6	7	5	375
GLQ/IG/AG	72	24	20	-	-	28	360
R19/SG/AW	71	52	3	-	6	10	355
R19	71	55	9	-	4	4	355
GVQ/IG/AL	69	54	8	1	1	6	345
R19/AW	66	44	-	-	6	17	330
R19/FI	66	26	4	15	4	17	330
KU3/AP/SW	62	12	14	6	12	18	310
RT2/SG/AW	55	37	-	-	-	18	275
K19/FV/EV	43	31	8	1	1	3	215
K19/FA/IG	41	13	18	-	1	10	205
GVQ/A328G	38	25	-	-	-	12	190
KSK19/FW	33	13	1	2	4	14	165
RP/AM/IA	29	-	13	-	-	16	145
K19/FV	22	14	1	-	1	5	110
R19/FA/AI	22	15	2	-	-	5	110
KSK19/IA	21	16	3	1	-	1	105
GVQ/A264G	18	9	-	-	5	4	90
R19/FA	12	-	-	-	-	12	60
RKA/SW	12	-	-	-	-	12	60
AP	10	-	-	-	-	10	50
K19/FV/AG	8	-	-	-	-	8	40
R19/AL/IA	8	-	-	-	-	8	40
RK/FW/TG/AG	7	-	-	-	-	7	35
RK/A328G	6	-	-	4	-	2	30
WT	6	-	-	-	-	6	30
KU3/AP/AI	3	-	-	-	-	3	15
RK/AG/PG/AG	3	-	-	-	-	3	15

RK/AL	3	-	-	-	-	3	15
K19/FA/AM/IG	2	-	-	-	-	2	10
R19/AL/AL	2	-	-	-	-	2	10
RK/AI/TG/AG	2	-	-	-	-	2	10
RKA/VI	2	-	-	-	-	2	10
RP/IA/EV	2	-	2	-	-	1	10
K19/FV/EV/VI	1	-	-	-	-	1	5
RK/A328G	1	-	-	-	-	1	5
RK/AG/AG	1	-	-	-	-	1	5
RK/TG	1	-	-	-	-	1	5

Table S4: Second generation library screening data for substrate **12b**, at 2.0 mM substrate concentration and 2.0 µM enzyme concentration (1000:1 substrate-to-enzyme ratio), ordered by conversion, focusing on optimizing for metabolite **18**.

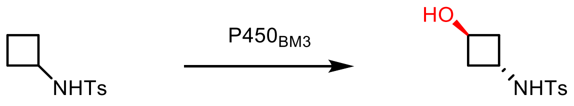

Variant	Conversion/%	18 /%	Other /%	TTN	TON
K19/FA/AI/IA	99	79	20	995	790
RT2/FW	95	83	11	950	830
RT2/A330W	82	67	16	825	665
KU3/AS/LLA	68	23	46	680	225
GVQ/SW	61	36	25	610	365
K19/FV/AL	54	17	37	540	175
R19/FI/AW	46	16	30	460	165
R19/AW	30	21	8	295	215
RP/EV	22	14	8	225	145
RT2/SG/AW/LLA	17	4	13	165	45
RT2/AP/AM	16	9	7	160	90
RT2/AP/VI	16	10	6	160	100
RP/FV/EV	14	7	6	140	75
RT2/IP	14	8	6	140	75
K19/FA/AI	13	7	6	135	70
K19/FA/AM	13	7	6	135	75
RT2/AP/AI	13	7	6	135	75
R19/FI/AP	12	4	9	125	40
RT2/AP/LLA	6	-	7	60	-
RT2/SG/AP/LLA	6	-	5	55	-
RT2	3	1	2	25	10
RP/EG	1	-	1	10	-

Table S5: First generation library screening data for substrate **12c**, at 1.0 mM substrate concentration and 2.0 μM enzyme concentration (500:1 substrate-to-enzyme ratio), ordered by conversion.

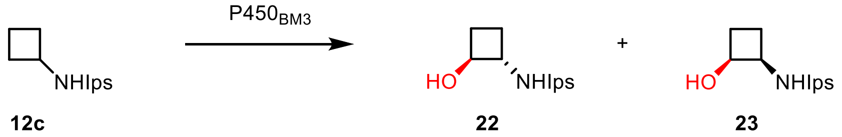

**Variant**	**Conversion /%**	**22 /%**	**23 /%**	**Other /%**	**TTN**
KU3/AP/SW	91	53	27	11	910
RK/TG	43	-	-	43	430
GV/AI	38	-	-	38	380
RP/HL/IG/AI	23	-	-	23	235
VQ/SG/AW	17	-	-	17	170
K19/FA/IG	12	-	-	12	115
K19/FV/EV	10	-	-	10	100

All other screened enzymes had <10% conversion

Table S6: First generation library screening data for substrate **13a**, at 2.0 mM substrate concentration and 2.0 µM enzyme concentration (1000:1 substrate-to-enzyme ratio), ordered by conversion.

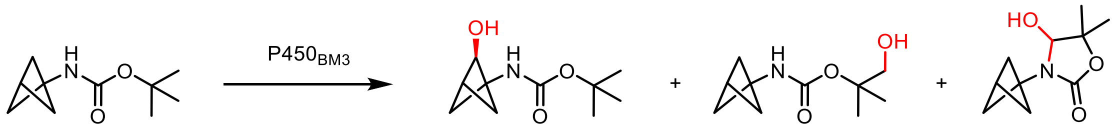

Variant	Conversion /%	26 /%	27 /%	28 /%	Other /%	TTN	TON	*ee* (*R*)-26 /%
R19/FI/AP[a]	79	14	32	33	-	795	140	-
GV/AI	58	40	5	-	13	585	395	66
K19/FV/EV	35	27	-	-	8	345	265	20
R19/FI/LLV	30	19	-	-	11	295	185	–21[b]
K19/FA/FW	29	20	4	-	5	290	205	76
RK/AG	27	15	3	-	9	270	145	10
R19/FA/AI	27	14	2	-	10	265	140	77
K19/FV/IG	26	14	-	-	12	260	145	–47[b]
R19/FA	24	10	3	-	11	245	100	63
VQ/SG/AW	23	7	6	-	9	225	75	16
RK/AG/PG/AG	22	13	-	-	10	220	125	-
K19/FV/QP	20	7	2	-	11	195	65	50
GVQ/IG	18	8	-	-	10	180	75	–58[b]
K19/FV/AG	17	2	-	-	15	170	20	–50[b]
RKA/SW	17	4	3	-	10	170	35	82
RKA/VI	16	2	-	-	14	165	25	82
K19/FV/EV/VI	16	-	-	-	16	160	-	-
GVQ/AW	13	3	2	-	8	135	35	11
K19/FA/IG	13	7	-	-	6	130	75	16
K19/FA/IA	13	8	1	-	4	130	75	50
GVQ/AG	13	2	-	-	11	125	20	2
RK/AL	12	3	1	-	8	125	35	82
RP/HL/IG/AI	12	-	-	-	12	120	-	-
GVQ/AG	11	-	-	-	11	115	-	-
WT	11	-	-	-	11	110	-	-
RK/AI/TG/AG	11	-	-	-	11	105	-	-
GVQ/IG/AL	10	-	-	-	10	105	-	-
KT2/LG/IG	10	-	-	-	10	100	-	-
R19/SG/AW	9	-	-	-	9	90	-	-
RK/FW/TG/AG	9	-	-	-	9	90	-	-
GLQ/IG/AG	9	-	-	-	9	90	-	-
KU3/AP/SW	9	-	2	-	6	85	-	-
RP/IA/EV	8	-	-	-	8	80	-	-
RK/AG/AG	8	-	-	-	8	80	-	-
R19/AL/IA	8	-	-	-	8	80	-	-
RK/TG	8	-	-	-	8	80	-	-
K19/FV	8	4	-	-	3	75	45	37
RP/AM/IA	7	-	-	-	7	75	-	-
AP	7	-	-	-	7	75	-	-

R19	7	-	-	-	7	73	-	-
RT2/SG/AW	7	-	-	-	7	70	-	-
R19/AL/AL	7	-	-	-	7	65	-	-
RP/HL/IG	6	-	-	-	6	60	-	-
GVQ	4	4	-	-	-	40	40	17
R19/FI	3	3	-	-	-	30	30	36
R19/AW	-	-	-	-	-	-	-	-

[a] This second-generation enzyme was included in the primary screen

[b] Negative values: (*S*)-**26** is major

Table S7: First generation library screening data for substrate **13d**, at 1.0 mM substrate concentration and 2.0 μM enzyme concentration (500:1 substrate-to-enzyme ratio), ordered by conversion.

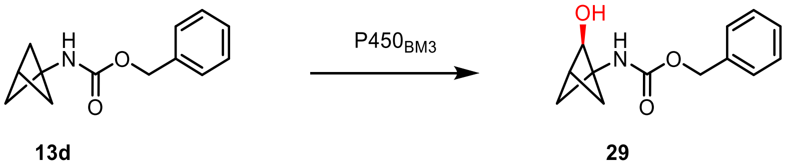

Variant	Conversion /%	29 /%	Other /%	TTN	TON	*ee* (*R*)-29 /%
K19/FV/QP	100	66	34	500	330	93
K19/FA/IG	100	61	39	500	305	12
K19/FA/FW	100	61	39	500	305	43
K19/FA/IA	100	49	51	500	245	49
GV/AI	100	23	77	500	115	-
VQ/SG/AW	100	11	89	500	55	-
RK/AG	100	-	100	500	-	-
R19/FA	95	72	23	475	360	83
RP/HL/IG/AI	95	12	84	475	60	-
KU3/AP/SW	94	44	50	470	220	96
GVQ/AW	94	24	70	470	120	-
R19	92	27	64	460	135	-
RP/AM/IA	91	7	84	455	35	-
RK/FW/TG/AG	91	-	91	455	-	-
R19/FA/AI	88	64	23	440	320	51
RT2/SG/AW	86	17	69	430	85	-
K19/FV	84	64	20	420	320	96
RK/AG/PG/AG	82	47	36	410	235	69
GVQ	78	55	23	390	275	95
GVQ/AG	77	19	58	385	95	24
KT2/LG/IG	69	8	61	345	40	-
K19/FV/AG	54	38	17	270	190	92
R19/AW	49	6	44	245	30	-
RK/AI/TG/AG	45	-	45	225	-	-
K19/FV/EV	39	33	6	195	165	98
GVQ/IG	38	29	9	190	145	72
K19/FV/IG	34	28	6	170	140	88
R19/FI/LLV	26	23	3	130	115	98
KU3/AP/AI	26	-	26	130	-	-
RK/TG	24	8	16	120	40	-
GQ/IG/AL	21	2	19	105	10	-
GVQ/AG	19	15	4	95	75	93
GV/AI/IG/AG	18	14	4	90	70	77
R19/FI	17	14	3	85	70	>99
K19/FV/EV/VI	14	4	10	70	20	-
RP/HL/IG	9	2	7	45	10	-
R19/SG/AW	9	1	7	45	5	-
RKA/SW	8	4	5	40	20	-
RK/AG/AG	6	-	6	30	-	-
RKA/VI	4	2	2	20	10	-
RP/IA/EV	4	1	3	20	5	-

RK/AL	3	2	1	15	10	-
GVQ/IG/AL	3	1	2	15	5	-
AP	3	-	2	15	-	-
GLQ/IG/AG	3	-	3	15	-	-
K19/FA/AM/IG	2	-	2	10	-	-
WT	2	-	2	10	-	-
R19/AL/IA	1	-	1	5	-	-

S3.3 Repeats and titer tests

Table (a) summarizes screening data from four repeats each of the oxidation of substrate **12a** with five representative enzymes; conversions and selectivities vary about the mid-point by no more than ±5% and ±8.5%, respectively. Reaction conditions: 10 mM substrate concentration (20 µL of 0.25 M stock solution in DMSO), 1.0 µM enzyme concentration (50 µL of 10 µM P450$_{BM3}$ stock solution in phosphate buffer), glucose (250 µL, 1.0 M solution in phosphate buffer), GDH (50 µL, 2.0 U/µL solution in phosphate buffer), NADP$^+$ monosodium salt (50 µL, 4.0 mM solution in phosphate buffer) in phosphate buffer (80 µL). Reactions were conducted in 24-well plates shaken at 120 rpm at 20 °C for 24 h and the content of each well was mixed with ethyl acetate (300 µL), vortexed in 1.5 mL microcentrifuge tubes for 30 s, and then centrifuged at 13,300 *g* for 1 min after which the organic layer was transferred into GC vials for GC analysis. Conversions are given to two significant figures.

(a) Repeats

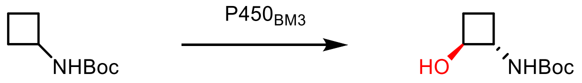

12a **14**

P450$_{BM3}$ variant	Conversion /%	TTN	Selectivity 14 /%	TON
IG	10	1020	39	400
	8.8	875	45	395
	8.0	805	44	355
	7.9	785	44	345
HL/IG	26	2640	40	1060
	28	2810	41	1140
	30	2960	42	1230
	30	2985	41	1220
RP/HL/IG	35	3500	62	2160
	37	3670	73	2670
	25	2545	79	1955
	33	3300	62	2030
RP/HL/IG/LLV	35	3475	73	2545
	29	2890	73	2110
	33	3290	72	2380
	34	3390	75	2545
RG/LLV	56	5600	77	4325
	55	5520	77	4240
	55	5535	79	4385
	53	5285	76	3990

(b) TTNs in the biocatalytic oxidation of substrate **12a** by two enzymes with varying relative substrate/enzyme concentration ratios.

RP/HL/IG/LLV		**[Substrate] /mM**					
		**0.5**	**1.0**	**2.0**	**4.0**	**8.0**	**10**
**[Enzyme] /µM**	**0.25**	1910	3420	7245	10580	6750	11950
	**0.5**	1000	2000	3930	6735	7350	8165
	**1.0**	500	1000	2000	3925	6370	7265
	**2.0**	250	500	1000	1990	3755	4500

RG/LLV		**[Substrate] /mM**					
		**0.5**	**1.0**	**2.0**	**4.0**	**8.0**	**10**
**[Enzyme] /µM**	**0.25**	1500	2105	2340	2285	4590	3525
	**0.5**	905	1595	2405	3030	4795	5145
	**1.0**	485	925	1615	2670	3790	3580
	**2.0**	250	485	920	1575	2430	2815

S4 Selected GC traces

S4.1 GC traces of screening reactions

In all traces, the x-axis is the retention time (minutes), the y-axis is the FID response (pA).

GC trace for a reaction of substrate **12a** with RP/HL/IG:

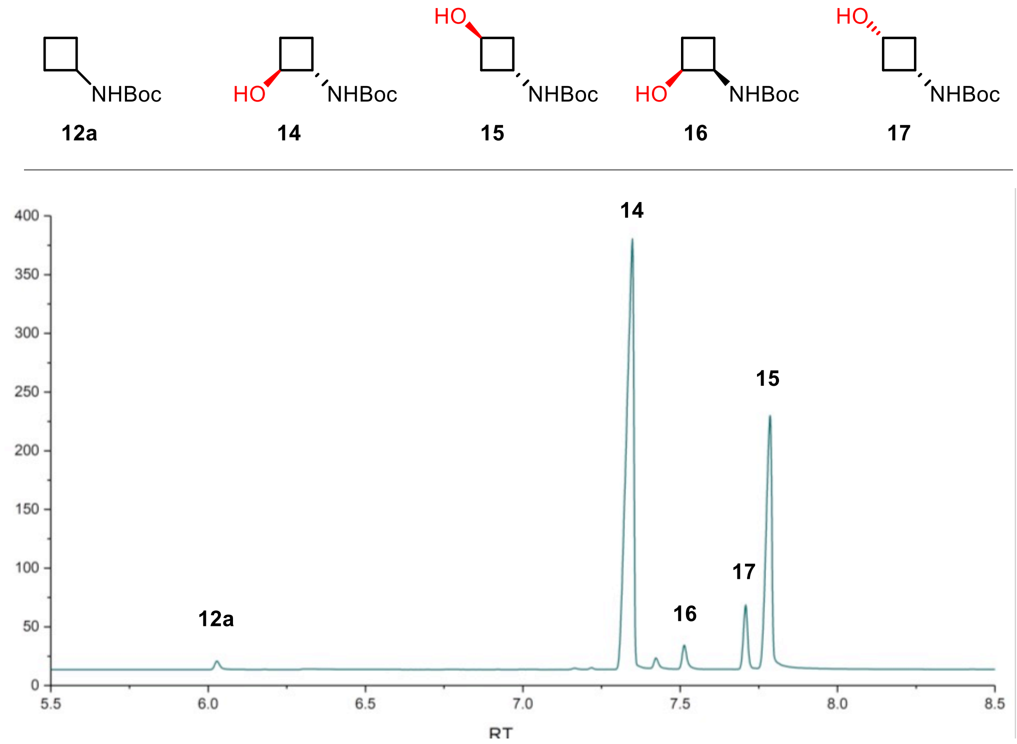

GC trace for a reaction of substrate **12b** with RT2/A330W:

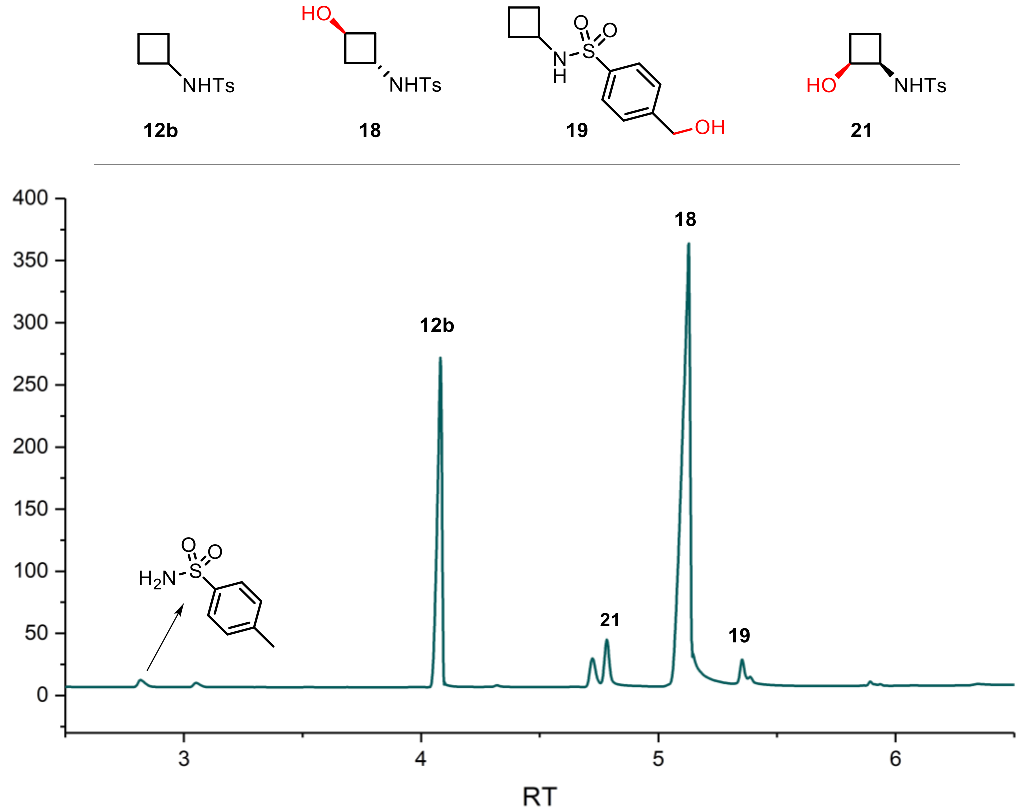

GC trace for a reaction of substrate **12c** with KU3/AP/SW:

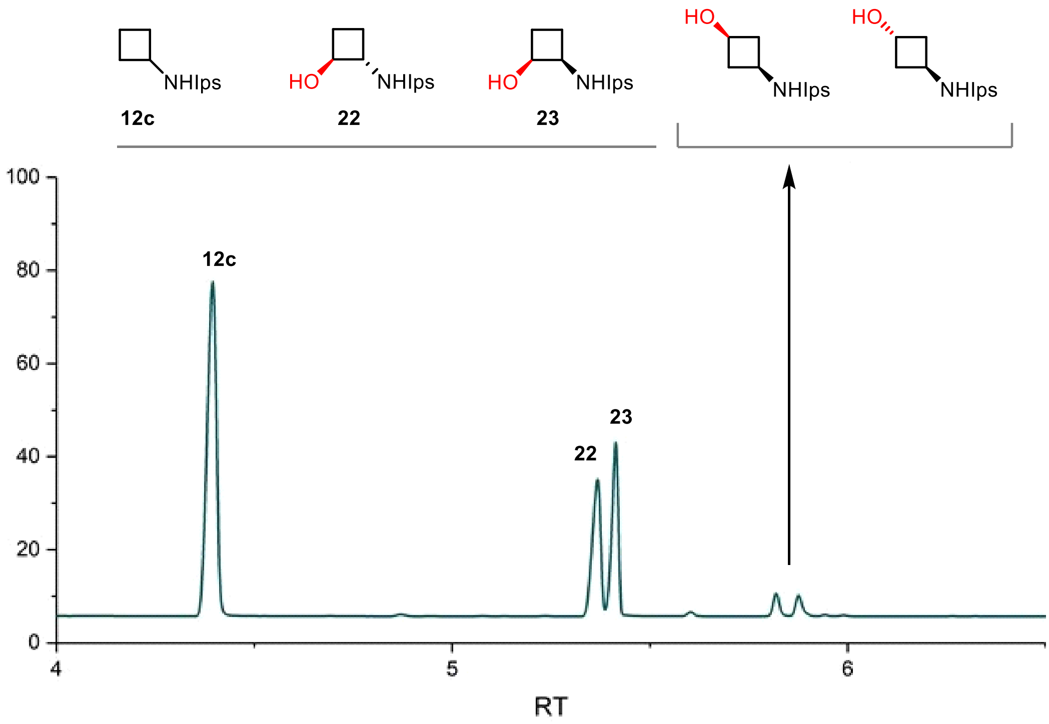

GC trace for a reaction of substrate **13a** with R19/FI/AP:

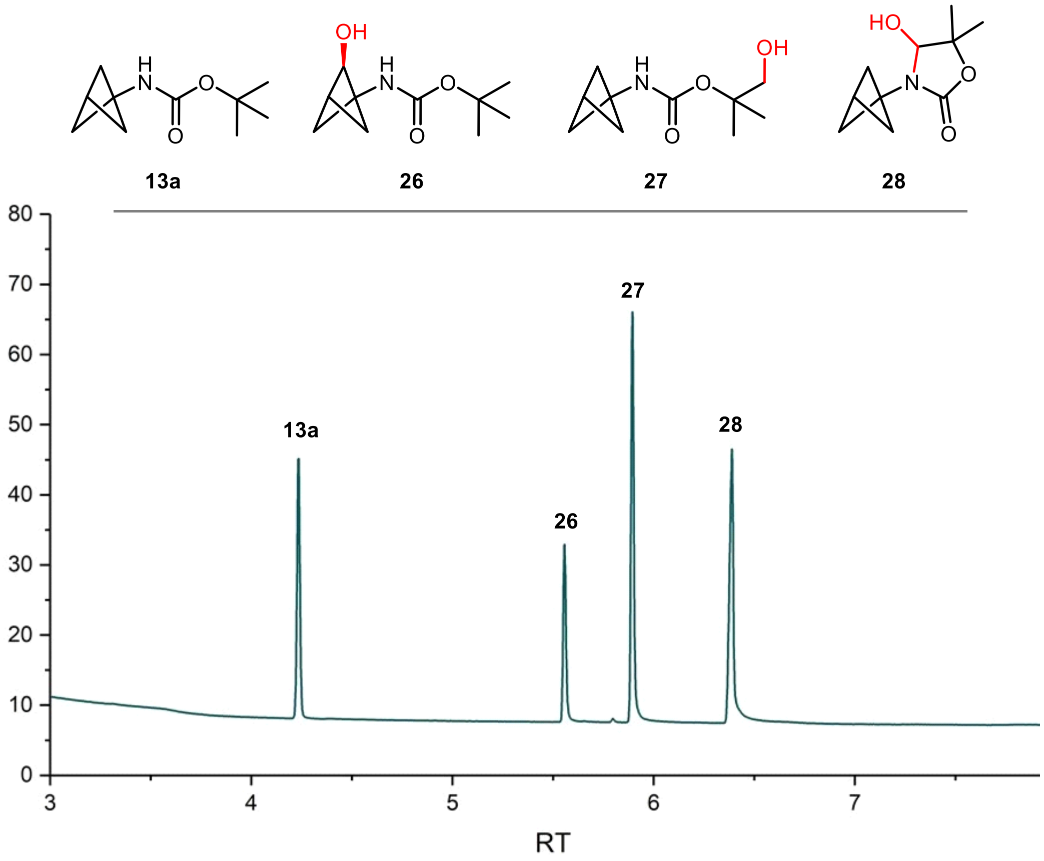

GC trace for a reaction of substrate **13d** with K19/FV/QP:

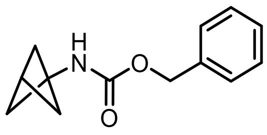

13d

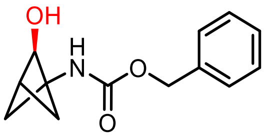

29

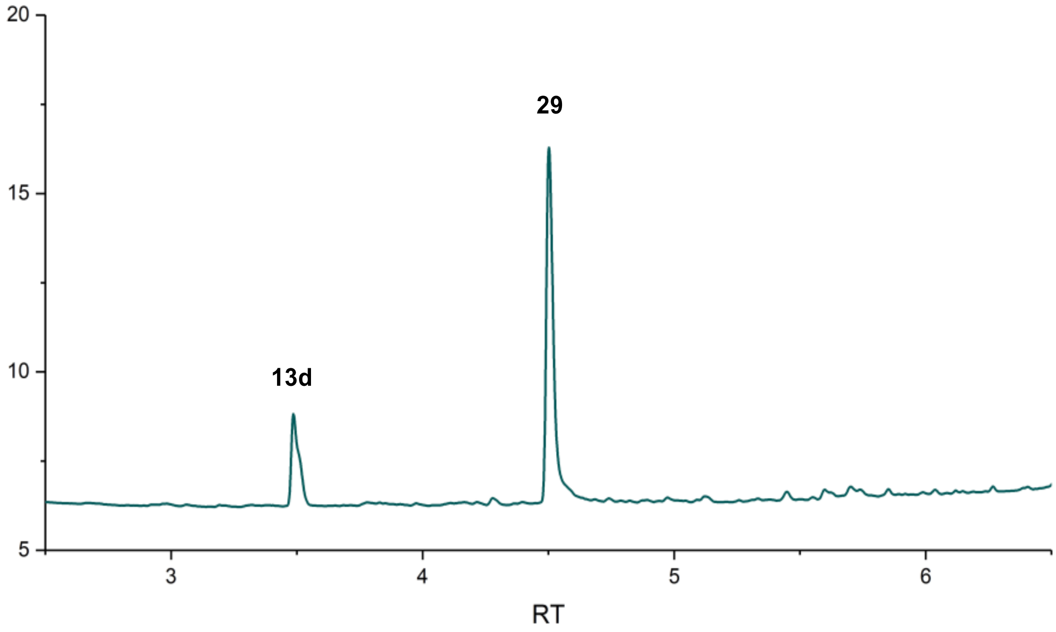

S4.2 Chiral GC traces of isolated metabolites

S4.2.1 *tert*-Butyl [(1*S*,2*S*)-2-hydroxycyclobutyl]carbamate 14

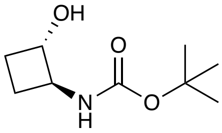

Column: Cyclosil-B [Length: 30 m, Diameter: 0.25 mm, Film thickness: 0.25 µm]

Ramp: t = 0 min: 90 °C; 0.5 °C/min between 90–130 °C; 130 °C hold = 10 min; 10 °C/min between 130–200 °C; 200 °C hold = 1 min.

Enantioenriched (from RG/LLV): 99.9% *ee*

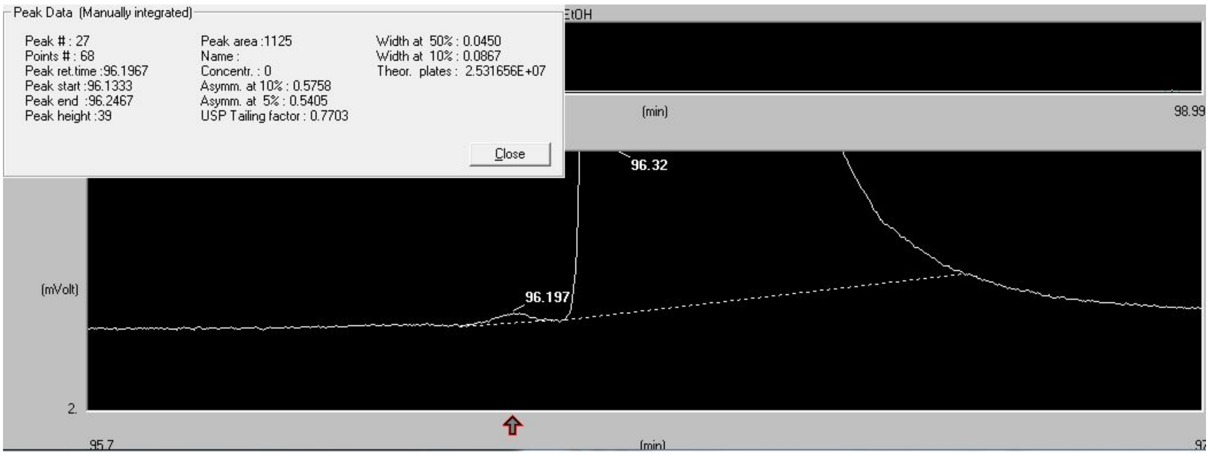

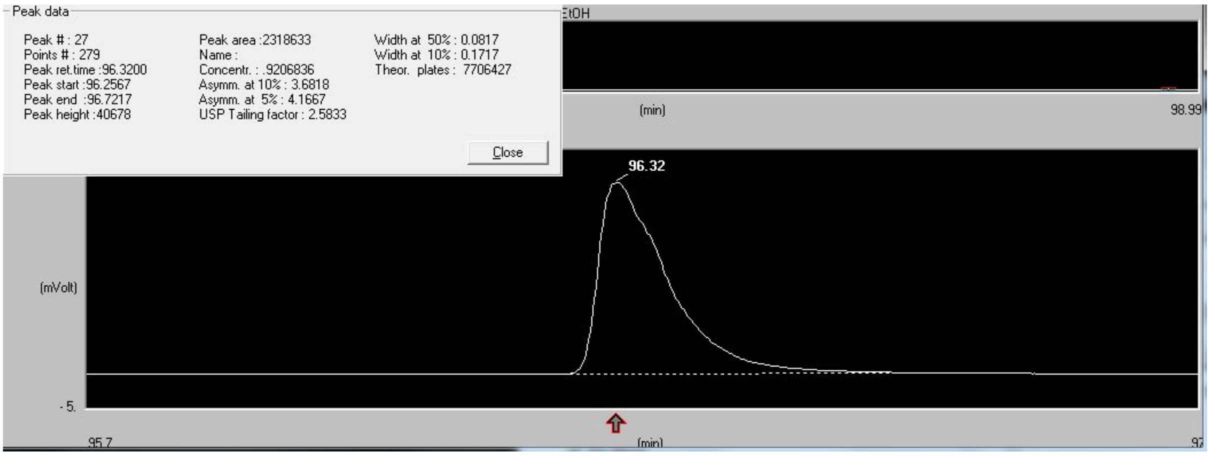

R_T	Peak Area	%
96.197	1125	0.0485
96.32	2318633	99.952

Racemic (sample synthesis described in **S5.5**):

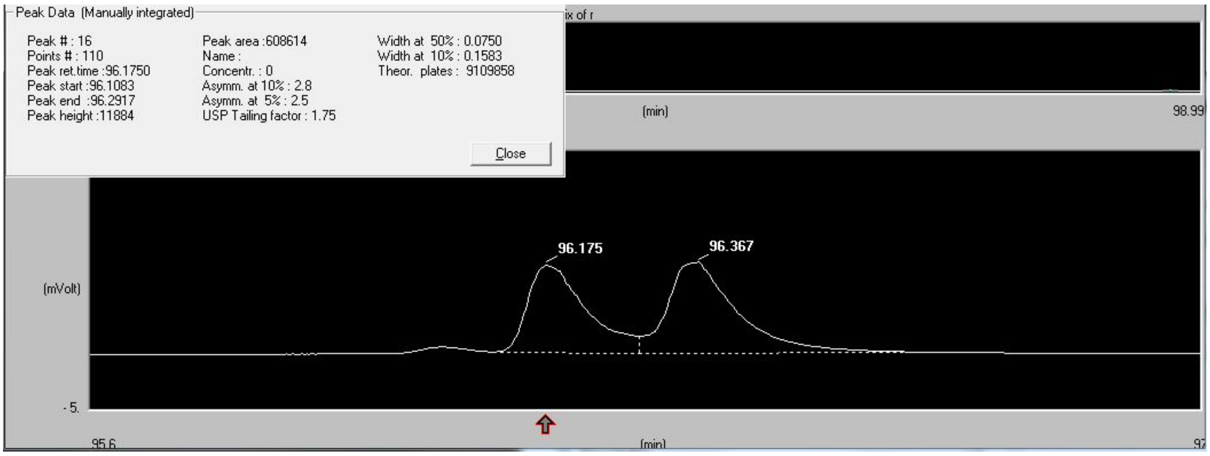

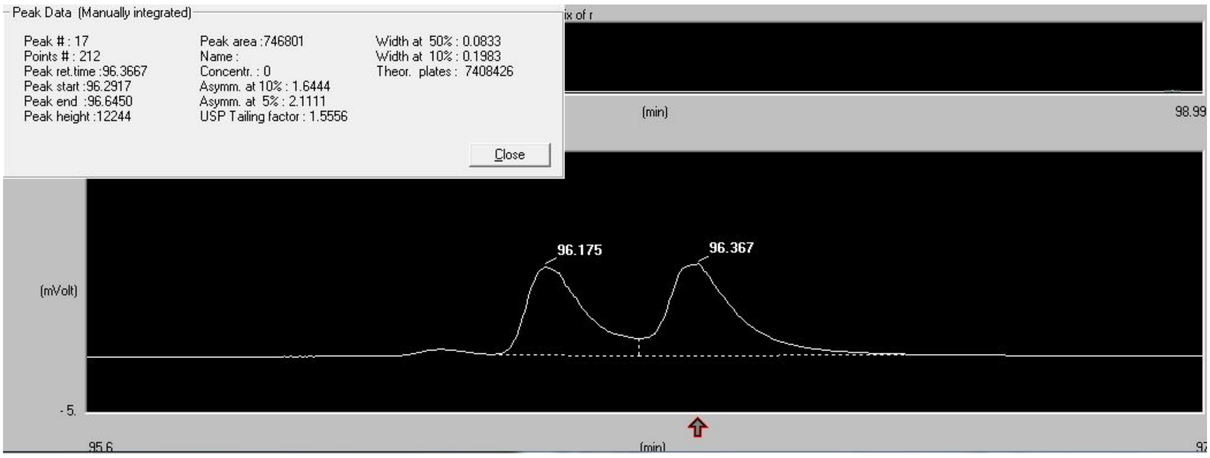

**RT**	**Peak Area**	**%**
96.175	608614	44.902
96.367	746801	55.098

S4.2.2 *tert*-Butyl [(1*R*,2*S*)-2-hydroxycyclobutyl]carbamate 16

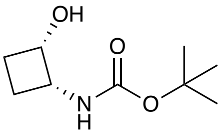

Column: Cyclosil-B [Length: 30 m, Diameter: 0.25 mm, Film thickness: 0.25 µm]

Ramp: t = 0 min: 90 °C; 1 °C/min between 90–180 °C; 10 °C/min between 180–200 °C; 200 °C hold = 1 min.

Enantioenriched (from RG/LLV): 93% *ee*

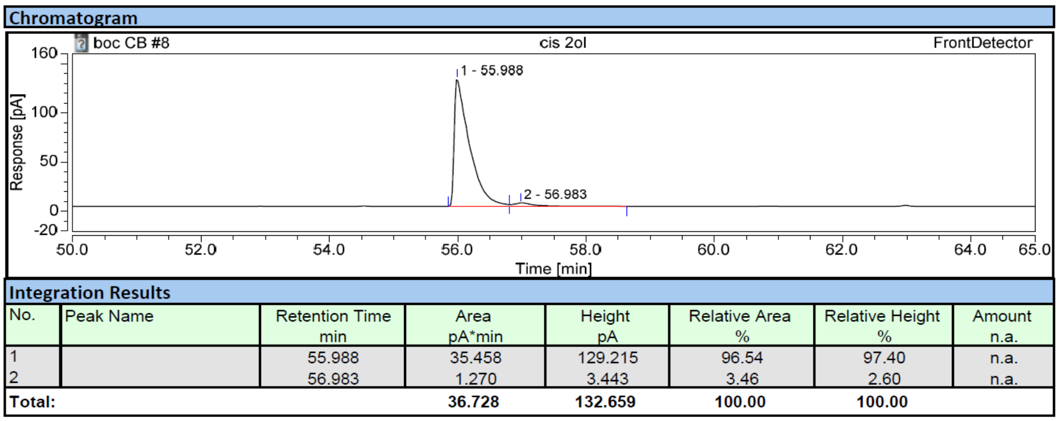

Integration Results

No.	Peak Name	Retention Time min	Area pA*min	Height pA	Relative Area %	Relative Height %	Amount n.a.
1		55.988	35.458	129.215	96.54	97.40	n.a.
2		56.983	1.270	3.443	3.46	2.60	n.a.
**Total:**			**36.728**	**132.659**	**100.00**	**100.00**	

Enantioenriched (from RK/AL): –27% *ee*

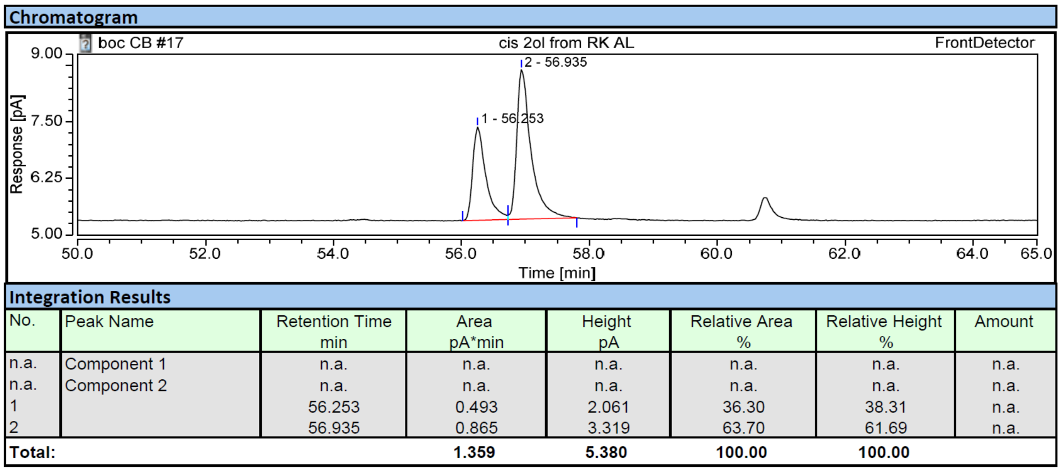

Integration Results

No.	Peak Name	Retention Time min	Area pA*min	Height pA	Relative Area %	Relative Height %	Amount
n.a.	Component 1	n.a.	n.a.	n.a.	n.a.	n.a.	n.a.
n.a.	Component 2	n.a.	n.a.	n.a.	n.a.	n.a.	n.a.
1		56.253	0.493	2.061	36.30	38.31	n.a.
2		56.935	0.865	3.319	63.70	61.69	n.a.
**Total:**			**1.359**	**5.380**	**100.00**	**100.00**	

Racemic (sample synthesis described in **S5.5**):

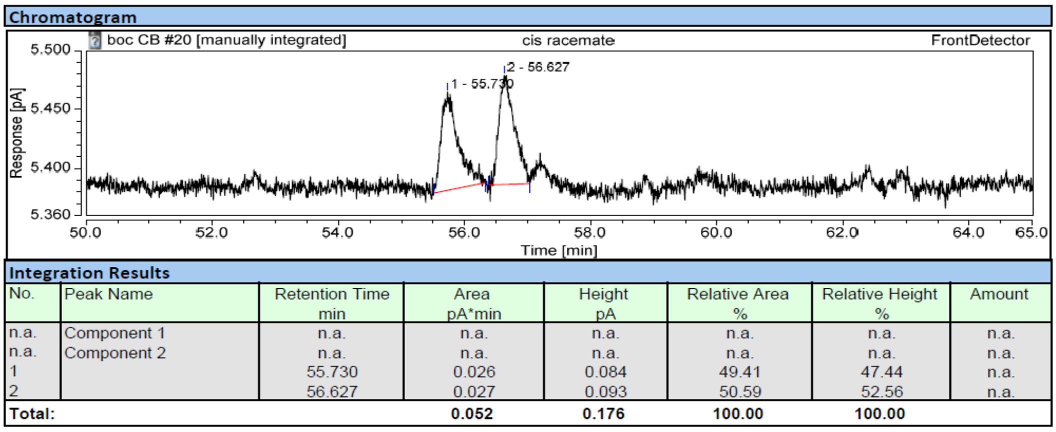

Integration Results

No.	Peak Name	Retention Time min	Area pA*min	Height pA	Relative Area %	Relative Height %	Amount
n.a.	Component 1	n.a.	n.a.	n.a.	n.a.	n.a.	n.a.
n.a.	Component 2	n.a.	n.a.	n.a.	n.a.	n.a.	n.a.
1		55.730	0.026	0.084	49.41	47.44	n.a.
2		56.627	0.027	0.093	50.59	52.56	n.a.
**Total:**			**0.052**	**0.176**	**100.00**	**100.00**	

S4.2.3 *tert*-Butyl [(*S*)-(2-oxocyclobutyl)]carbamate 31

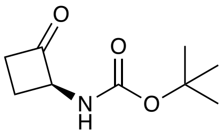

Column: Cyclosil-B [Length: 30 m, Diameter: 0.25 mm, Film thickness: 0.25 μm]

Ramp: t = 0 min: 90 °C; 0.5 °C/min between 90–140 °C; 140 °C hold = 10 min; 10 °C/min between 140–200 °C; 200 °C hold = 1 min.

Enantioenriched (from the reaction described in **S5.4**): 98% *ee*

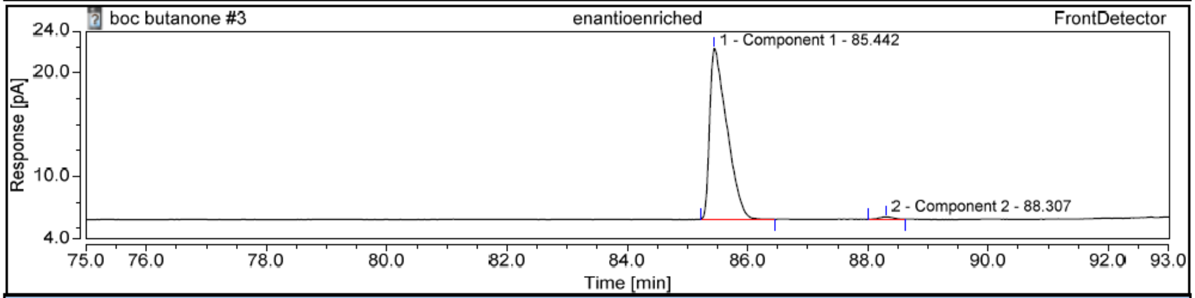

Integration Results

No.	Peak Name	Retention Time min	Area pA*min	Height pA	Relative Area %	Relative Height %	Amount
1	Component 1	85.442	5.426	16.446	98.84	98.59	n.a.
2	Component 2	88.307	0.064	0.236	1.16	1.41	n.a.
**Total:**			**5.489**	**16.682**	**100.00**	**100.00**	

Racemic (sample synthesis described in **S5.5**):

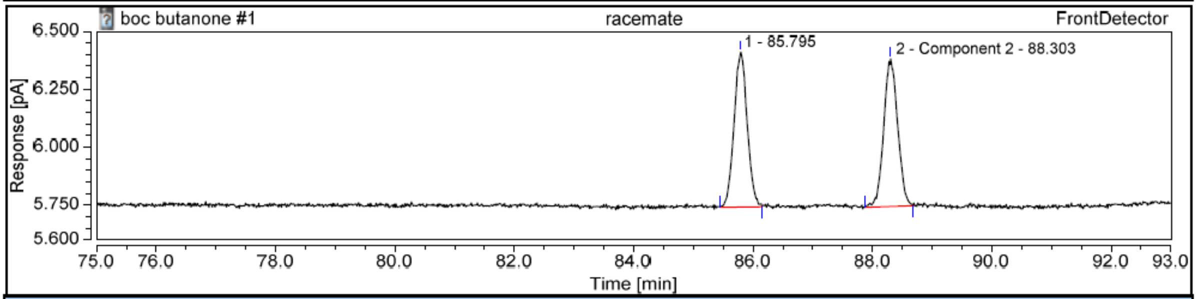

Integration Results

No.	Peak Name	Retention Time min	Area pA*min	Height pA	Relative Area %	Relative Height %	Amount
n.a.	Component 1	n.a.	n.a.	n.a.	n.a.	n.a.	n.a.
1		85.795	0.171	0.670	49.77	51.19	n.a.
2	Component 2	88.303	0.172	0.639	50.23	48.81	n.a.
**Total:**			**0.343**	**1.309**	**100.00**	**100.00**	

S4.2.4 *tert*-Butyl [(2*R*)-2-hydroxybicyclo[1.1.1]pent-1-yl]carbamate 26

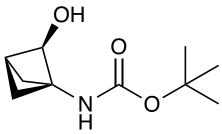

Column: Cyclosil-B [Length: 30 m, Diameter: 0.25 mm, Film thickness: 0.25 μm]

Ramp: t = 0 min: 60 °C; 1 °C/min between 60–180 °C; 180 °C hold = 10 min; 20 °C/min between 180–200 °C; 200 °C hold = 1 min.

Enantioenriched (from GV/AI): after purification by column chromatography: 67% *ee*

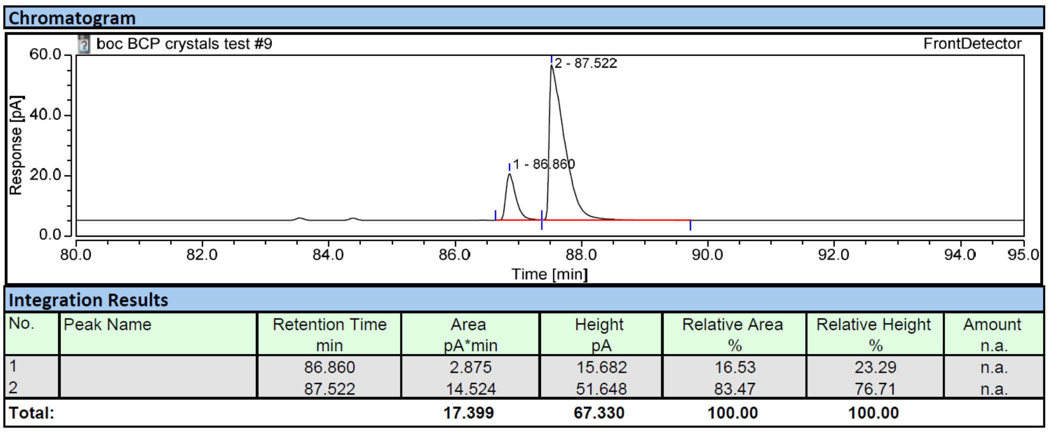

Integration Results

No.	Peak Name	Retention Time<br>min	Area<br>pA*min	Height<br>pA	Relative Area<br>%	Relative Height<br>%	Amount<br>n.a.
1		86.860	2.875	15.682	16.53	23.29	n.a.
2		87.522	14.524	51.648	83.47	76.71	n.a.
**Total:**			**17.399**	**67.330**	**100.00**	**100.00**	

After first recrystallization: 87% *ee*

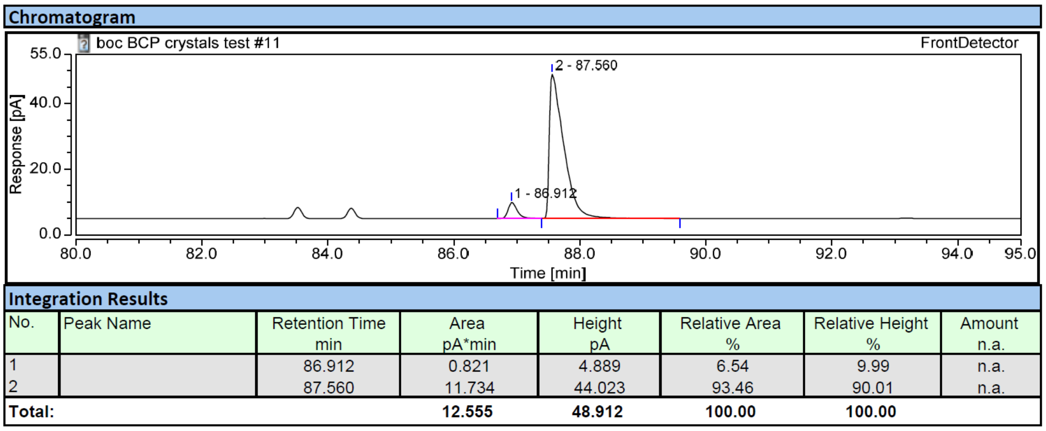

Integration Results

No.	Peak Name	Retention Time<br>min	Area<br>pA*min	Height<br>pA	Relative Area<br>%	Relative Height<br>%	Amount<br>n.a.
1		86.912	0.821	4.889	6.54	9.99	n.a.
2		87.560	11.734	44.023	93.46	90.01	n.a.
**Total:**			**12.555**	**48.912**	**100.00**	**100.00**	

After second recrystallization: 91% *ee*

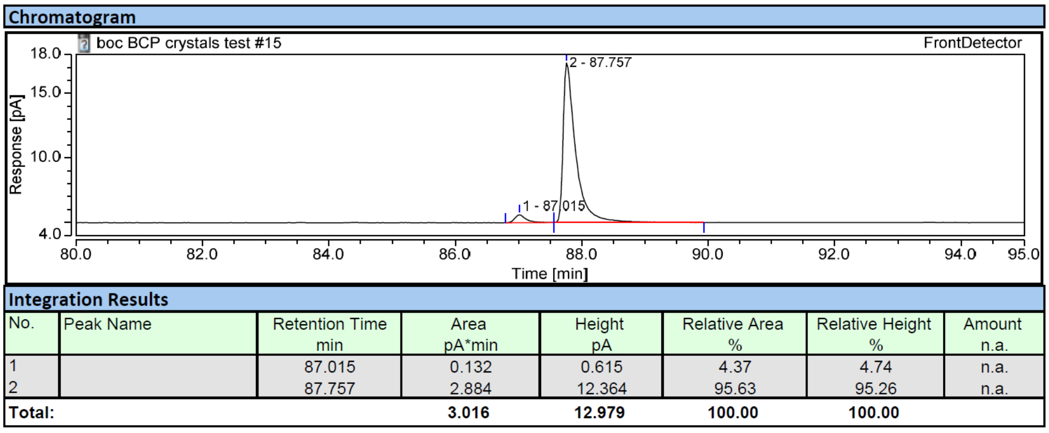

Integration Results

No.	Peak Name	Retention Time<br>min	Area<br>pA*min	Height<br>pA	Relative Area<br>%	Relative Height<br>%	Amount<br>n.a.
1		87.015	0.132	0.615	4.37	4.74	n.a.
2		87.757	2.884	12.364	95.63	95.26	n.a.
**Total:**			**3.016**	**12.979**	**100.00**	**100.00**	

S4.2.5 *tert*-Butyl [(2*S*)-2-hydroxybicyclo[1.1.1]pent-1-yl]carbamate 26

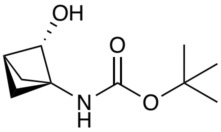

Column: Cyclosil-B [Length: 30 m, Diameter: 0.25 mm, Film thickness: 0.25 µm]

Ramp: t = 0 min: 60 °C; 1 °C/min between 60–180 °C; 180 °C hold = 10 min.

Enantioenriched (from K19/FV/IG/A330F): after purification by column chromatography: 82% *ee*

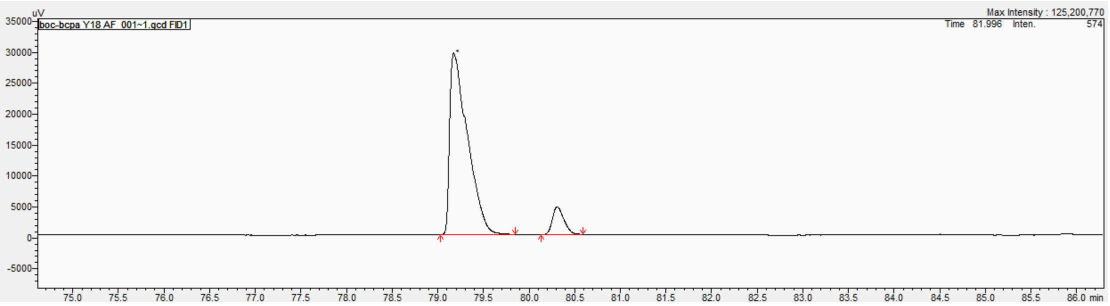

Peak#	Ret.	Area	Area%	Height	Height%
1	79.17	398200	90.888	29328	86.614
2	80.30	39922	9.112	4533	13.386
Total		438122	100.000	33860	100.000

S5 Synthetic chemistry

S5.1 Synthesis of substrates

tert-Butyl cyclobutylcarbamate 12a

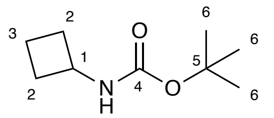

To a stirred solution of aminocyclobutane (100 mg, 1.41 mmol) in dichloromethane (3 mL) at 0 °C, was added a solution of di-*tert*-butyl dicarbonate (337 mg, 1.54 mmol) in dichloromethane (2 mL) dropwise. The reaction mixture was warmed to RT and stirred for 1 h. Upon completion, the solution was washed sequentially with water (2 × 10 mL) and brine (10 mL), then dried ($MgSO_4$), and filtered. The solvent was removed *in vacuo* yielding the title compound as a colorless crystalline solid (191 mg, 79%) which was used without further purification. **R_f** 0.23 (5% EtOAc in pentane). **^{1}H NMR** (400 MHz, $CDCl_3$) δ 1.42 (s, 9H, H6), 1.58–1.67 (m, 2H, H3), 1.75–1.84 (m, 2H, H2), 2.26–2.32 (m, 2H, H2), 4.00–4.15 (m, 1H, H1), 4.64 (br. s, 1H, NH). **^{13}C NMR** (101 MHz, $CDCl_3$) δ 14.9 (C3), 28.5 (C6), 31.6 (C2), 46.0 (C1), 79.2 (C5), 155.0 (C4). **HRMS** (ESI+) Found: 194.1154, $C_9H_{17}NO_2Na$ $[M+Na]^+$ requires: 194.1151. **MP** 72–74 °C. Data match those reported.[4]

N-Cyclobutyl-4-methylbenzenesulfonamide 12b

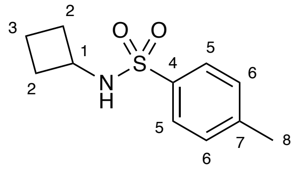

To a stirred solution of aminocyclobutane (100 mg, 1.41 mmol) in dichloromethane (9.5 mL) at 0 °C under argon, was added triethylamine (983 µL, 7.05 mmol) followed by *p*-toluenesulfonyl chloride (538 µL, 2.82 mmol) and the mixture was allowed to return to RT and stirred overnight. When complete, the reaction was quenched with sat. aq. $NaHCO_3$ solution (10 mL), extracted with dichloromethane (3 × 20 mL) and the combined organic portions were dried ($MgSO_4$), filtered, and concentrated *in vacuo*. The crude product was purified by column chromatography (30% EtOAc in pentane) to yield the sulfonamide as a yellow oil (251 mg, 79%). **R_f** 0.54 (50% EtOAc in pentane). **^{1}H NMR** (400 MHz, $CDCl_3$) δ 1.48–1.59 (m, 2H, H3), 1.72–1.81 (m, 2H, H2), 2.03–2.10 (m, 2H, H2), 2.40 (s, 3H, H8), 3.72–3.80 (m, 1H, H1), 5.20 (br. d, *J* = 9.0 Hz, 1H, NH), 7.28 (d, *J* = 8.5 Hz, 2H, H6), 7.75 (d, *J* = 8.5 Hz, 2H, H5). **^{13}C NMR** (101 MHz, $CDCl_3$) δ 15.1 (C3), 21.6 (C8), 31.8 (C2), 48.3 (C1), 127.2 (C5), 129.8 (C6), 138.1 (C7), 143.5 (C4). **HRMS** (ESI+) Found: 226.0897, $C_{11}H_{16}NO_2S$ $[M+H]^+$ requires: 226.0896. Data match those reported.[5]

N-Cyclobutylpropane-2-sulfonamide 12c

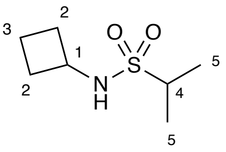

To a stirred solution of aminocyclobutane (100 mg, 1.41 mmol) in dichloromethane (9.5 mL) at 0 °C under argon was added triethylamine (983 μL, 7.05 mmol) followed by 2-propanesulfonyl chloride (174 μL, 1.55 mmol); the cold bath was removed and the mixture was stirred at RT for 14 h. The reaction was quenched with sat. aq. $NaHCO_3$ solution (10 mL), extracted with dichloromethane (3 × 20 mL) and the combined organic portions were dried ($MgSO_4$), filtered, and concentrated *in vacuo*. The crude product was purified by column chromatography (10–30% EtOAc in pentane) to yield the product as a yellow crystalline solid (111 mg, 45%). **R*f*** 0.60 (50% EtOAc in pentane). **^{1}H NMR** (400 MHz, $CDCl_3$) δ 1.35 (d, *J* = 7.0 Hz, 6H, H5), 1.58–1.69 (m, 2H, H3), 1.90–1.99 (m, 2H, H2), 2.32–2.39 (m, 2H, H2), 3.08 (sept, *J* = 7.0 Hz, 1H, H4), 3.86–3.96 (m, 1H, H1), 4.44 (br. d, *J* = 8.5 Hz, 1H, NH). **^{13}C NMR** (101 MHz, $CDCl_3$) δ 14.5 (C3), 16.8 (C5), 32.9 (C2), 48.7 (C1), 53.9 (C4). **HRMS** (ESI+) Found: 200.0718, $C_7H_{15}NO_2SNa$ $[M+Na]^+$ requires: 200.0716. **MP** 74–76 °C.

***tert*-Butyl bicyclo[1.1.1]pent-1-ylcarbamate 13a**[6]

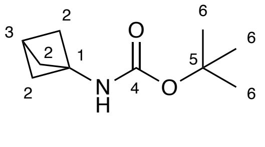

To a stirred suspension of 1-bicyclo[1.1.1]pentylamine hydrochloride (50.0 mg, 0.418 mmol) and NaH (42.0 mg, 1.05 mmol, 60% in mineral oil) in dry THF (2.1 mL) under argon at 0 °C, was added di-*tert*-butyl dicarbonate (137 mg, 0.628 mmol). The mixture was allowed to warm to RT and then stirred for 21 h. The reaction mixture was cooled to 0 °C, quenched slowly with water (2 mL), and then extracted with ethyl acetate (2 × 10 mL); the combined organic portions were dried ($MgSO_4$), filtered, and concentrated *in vacuo*. The crude product was purified by column chromatography (5–15% EtOAc in pentane) to yield the title compound as a colorless solid (54.5 mg, 71%). **R*f*** 0.52 (20% EtOAc in pentane). **^{1}H NMR** (400 MHz, $CDCl_3$) δ 1.44 (s, 9H, H6), 2.00 (s, 6H, H2), 2.38 (s, 1H, H3), 4.94 (br. s, 1H, NH). **^{13}C NMR** (126 MHz, $CDCl_3$) δ 24.2 (C3), 28.6 (C6), 48.7 (C1), 52.6 (C2), 79.5 (C5), 155.1 (C4). **HRMS** (ESI+) Found: 206.1155, $C_{10}H_{17}NO_2Na$ $[M+Na]^+$ requires: 206.1151. **IR** ν_{max} (thin film)/cm^{-1} 3340m, 2876w, 1690s, 1514s, 1367m, 1146m. **MP** 98–99 °C.

Benzyl bicyclo[1.1.1]pent-1-ylcarbamate 13d

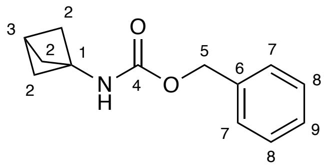

To a stirred suspension of 1-bicyclo[1.1.1]pentylamine hydrochloride (400 mg, 3.34 mmol) in dichloromethane (16 mL) under argon at 0 °C was added dropwise benzyl chloroformate (440 µL, 3.08 mmol) and triethylamine (1.03 mL, 7.46 mmol). The mixture was allowed to warm to RT and then stirred for 18 h. The solution was washed with sat. aq. $NaHCO_3$ solution (30 mL) and the combined organic portions were extracted with ethyl acetate (2 × 50 mL), dried ($MgSO_4$), filtered, and concentrated *in vacuo*. The crude product was purified by column chromatography (10–40% EtOAc in pentane) to yield the title compound as a yellow oil (329 mg, 49%). $\mathbf{R}_f$ 0.36 (50% EtOAc in pentane). **^{1}H NMR** (400 MHz, $CDCl_3$) δ 2.04 (s, 6H, H2), 2.41 (s, 1H, H3), 5.08 (s, 2H, H5), 5.22 (br. s, 1H, NH), 7.32–7.36 (m, 5H, H7,8,9). **^{13}C NMR** (101 MHz, $CDCl_3$) δ 24.1 (C3), 48.7 (C1), 52.6 (C2), 66.5 (C5), 128.2, 128.6 and 136.6 (C7,8,9) [C4, C6 not observed]. **HRMS** (ESI+) Found: 240.0996, $C_{13}H_{15}NO_2Na$ $[M+Na]^+$ requires: 240.0995. **IR** ν_{max} (thin film)/cm^{-1} 3320w, 2877w, 1699s, 1518m, 1454m, 1244s, 1202s, 1057s, 731s, 696s.

S5.2 Semi-preparative hydroxylations for product characterization

General procedure

The substrate (as a solution in the solvent specified) was added to a solution of the $P450_{BM3}$ variant (stored in phosphate buffer at the specified concentration), glucose (1.0 M solution in phosphate buffer), GDH (2.0 U/μL solution in phosphate buffer), and $NADP^+$ monosodium salt (4.0 mM solution in buffer) in phosphate buffer. After shaking at 120 rpm at 20 °C for the specified reaction time, ethyl acetate (2 × reaction volume) was added; the biphasic mixture was transferred into 50 mL centrifuge tubes (Falcon), shaken for 30 seconds and then centrifuged at 9,500 *g* for 4 min after which the organic layer was removed. This extraction process was repeated three further times. The combined organic extracts were dried ($MgSO_4$), filtered, and concentrated in vacuo to give the crude product which was then purified by column chromatography.

tert-Butyl [(1*S*,2*S*)-2-hydroxycyclobutyl]carbamate 14,[7] and *trans*- and *cis*-*tert*-Butyl (3-hydroxycyclobutyl)carbamate 15[8] and 17[8]

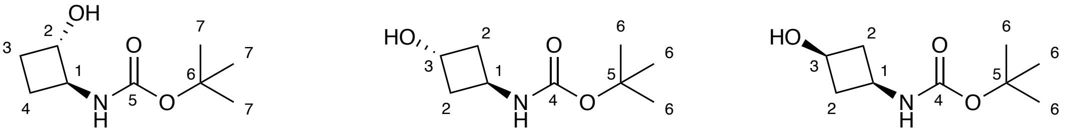

Prepared according to the general procedure, using variant RP/HL/IG (4.0 mL of 75 μM concentration in phosphate buffer, overall concentration of 2.0 μM), glucose (15 mL of 1.0 M stock in phosphate buffer), GDH (3.0 mL of 2.0 U/μL stock in phosphate buffer), $NADP^+$ monosodium salt (3.0 mL of 4.0 mM stock in phosphate buffer) in phosphate buffer (122 mL) and substrate **12a** (3.0 mL of 0.2 M stock in DMSO, 0.6 mmol, overall concentration of 4.0 mM) stirring for 48 h. Purification by column chromatography (20–60% EtOAc in pentane) yielded the 1,2-*trans* diastereomer **14** as a colorless crystalline solid (42.8 mg, 38%). $\mathbf{R}_f$ 0.34 (50% EtOAc in pentane). **^{1}H NMR** (400 MHz, $CDCl_3$) δ 1.23–1.33 (m, 1H, H4), 1.44 (s, 9H, H7), 1.56–1.66 (m, 1H, H3), 2.00–2.04 (m, 1H, H4), 2.07–2.13 (m, 1H, H3), 3.18 (br. s, 1H, OH), 3.66–3.72 (m, 1H, H1), 3.95 (app. q, *J* = 7.5 Hz, 1H, H2), 4.81 (br. s, 1H, NH). **^{13}C NMR** (101 MHz, $CDCl_3$) δ 19.4 (C4), 25.0 (C3), 28.5 (C7), 55.5 (C1), 73.6 (C2), 79.8 (C6), 156.0 (C5). **HRMS** (ESI+) Found: 210.1102, $C_9H_{17}NO_3Na$ $[M+Na]^+$ requires: 210.1101. **IR** ν_{max} (thin film)/cm^{-1} 3235br.w, 2988w, 1681s, 1531s, 1365m, 1277m, 1165s, 1106m, 1020m. $[\alpha]_D^{25}$ –11.3 (*c* = 1.0, $CHCl_3$) [lit.[7] $[\alpha]_D^{20}$ –21.2 (*c* = 0.5, $CHCl_3$)]. **MP** 101–103 °C [lit.[7] MP 118–119 °C]. See S4 for GC traces showing % *ee* and S8 for crystallographic data confirming absolute configuration.

From the same experiment a mixture of the 1,3-hydroxylated diastereomers was isolated as a colorless crystalline solid (18.4 mg, 16%, 5:1 *trans*/*cis*). $\mathbf{R}_f$ 0.29 (50% EtOAc in pentane). ***Trans* diastereomer 15**: **^{1}H NMR** (500 MHz, $CDCl_3$) δ 1.43 (s, 9H, H6), 2.17–2.25 (m, 2H, H2), 2.27–2.33 (m, 2H, H2), 4.21 (br. s, 1H, H1), 4.43–4.49 (m, 1H, H3), 4.71 (br. s, 1H, NH). **^{13}C NMR** (126 MHz, $CDCl_3$) δ 28.5 (C6), 40.3 (C2), 42.2 (C1), 65.0 (C3), 77.4 (C5), 155.4 (C4). ***Cis* diastereomer 17**: **^{1}H NMR** (500 MHz, $CDCl_3$) δ 1.43 (s, 9H, H6), 1.76–1.82 (m, 2H, H2), 2.71–2.78 (m, 2H, H2), 3.59–3.67 (m, 1H, H1), 4.00 (quin, *J* = 7.0 Hz, 1H, H3). **^{13}C**

NMR (126 MHz, $CDCl_3$) δ 28.5 (C6), 37.3 (C1), 41.9 (C2), 61.0 (C3), 79.6 (C5), 155.2 (C4). **HRMS** (ESI+) Found: 210.1103, $C_9H_{17}NO_3Na$ $[M+Na]^+$ requires: 210.1101.

***tert*-Butyl [(1*R*,2*S*)-2-hydroxycyclobutyl]carbamate 16**[7]

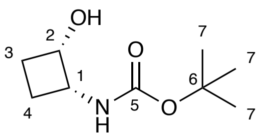

Isolated as a minor product from a preparation of (1*S*,2*S*)-**14** according to the general procedure, using variant RG/LLV (3.0 mL of 65 μM concentration in phosphate buffer, overall concentration of 2.0 μM), glucose (9.0 g), GDH (80 mg), $NADP^+$ monosodium salt (30 mg) in phosphate buffer (95 mL) and substrate **12a** (171 mg neat solid, 1.0 mmol, overall concentration of 10 mM) stirring for 24 h. Purification by column chromatography (30% EtOAc in pentane) yielded the title compound as a colorless oil (10.3 mg, 6%). $\mathbf{R_f}$ 0.41 (50% EtOAc in pentane). ^{1}**H NMR** (500 MHz, $CDCl_3$) δ 1.43 (s, 9H, H7), 1.81–1.86 (m, 1H, H3), 1.91–1.97 (m, 1H, H4), 2.09–2.14 (m, 2H, H3,4), 2.67 (br. s, 1H, OH), 4.08–4.11 (m, 1H, H1), 4.42 (br. s, 1H, H2), 5.18 (br. d, J = 4.5 Hz, 1H, NH). ^{13}**C NMR** (126 MHz, $CDCl_3$) δ 25.6 (C4), 27.3 (C3), 28.5 (C7), 50.0 (C1), 69.8 (C2), 79.7 (C6), 156.1 (C5). **HRMS** (ESI+) Found: 210.1104, $C_9H_{17}NO_3Na$ $[M+Na]^+$ requires: 210.1101. **IR** ν_{max} (thin film)/cm^{-1} 3421br.w, 2926w, 1686s, 1504m, 1391m, 1252m, 1168s. $[\alpha]_D^{25}$ +16.9 (c = 1.08, $CHCl_3$). See S4 for GC traces showing % *ee* and S6 for determination of absolute configuration by chemical correlation.

***N*-[(*trans*)-3-Hydroxycyclobutyl]-4-methylbenzenesulfonamide 18 and *N*-[(1*S*,2*R*)-2-hydroxycyclobutyl]-4-methylbenzenesulfonamide 21**

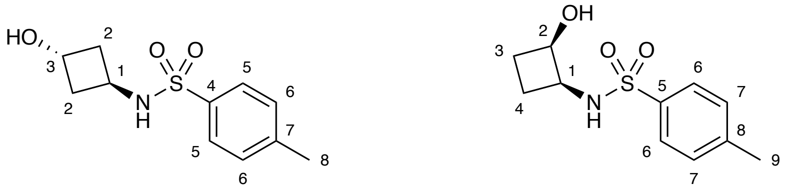

Prepared according to the general procedure, using variant RT2/AW (0.9 mL of 110 μM concentration in phosphate buffer, overall concentration of 2.0 μM), glucose (5.0 mL of 1.0 M stock in phosphate buffer), GDH (1.0 mL of 2.0 U/μL stock in phosphate buffer), $NADP^+$ monosodium salt (1.0 ml of 4.0 mM stock in phosphate buffer) in phosphate buffer (41 mL) and substrate **12b** (1.0 mL of 0.2 M stock in DMSO, 0.2 mmol, overall concentration of 4.0 mM) stirring for 48 h. Purification by column chromatography (20–70% EtOAc in pentane) yielded the 1,3-*trans* hydroxylated isomer **18** as a colorless crystalline solid (31.2 mg, 65%). $\mathbf{R_f}$ 0.18 (50% EtOAc in pentane). ^{1}**H NMR** (400 MHz, DMSO-d_6) δ 1.83–1.89 (m, 2H, H2), 1.92–1.96 (m, 2H, H2), 2.39 (s, 3H, H8), 3.65–3.74 (m, 1H, H1), 4.09–4.15 (m, 1H, H3), 4.90 (d, J = 4.5 Hz, 1H, OH), 7.39 (d, J = 8.0 Hz, 2H, H6), 7.65 (d, J = 8.0 Hz, 2H, H5), 7.80 (d, J = 7.5 Hz, 1H, NH). ^{13}**C NMR** (101 MHz, DMSO-d_6) δ 20.9 (C8), 39.4 (C2), 44.4 (C1), 62.6 (C3), 126.5 (C6), 129.5 (C5), 138.3 (C7), 142.5 (C4). **HRMS** (ESI+)

Found: 242.0842, $C_{11}H_{16}NO_3S$ $[M+H]^+$ requires: 242.0845. **IR** ν_{max} (thin film)/cm^{-1} 3267br.w, 2936w, 1305m, 1157s, 1092s, 988m, 815m, 665m. **MP** 124–126 °C.

From a separate experiment (0.6 mmol of substrate), a sample of the 1,2-*cis* regioisomer **21** was obtained, as a crystalline solid (1.2 mg, 6%). $\mathbf{R}_f$ 0.19 (50% EtOAc in pentane). ^{1}**H NMR** (500 MHz, $CDCl_3$) δ 1.75–1.79 (m, 1H, H3), 1.99–2.10 (m, 3H, H3,4), 2.43 (s, 3H, H9), 3.73–3.80 (m, 1H, H1), 4.27–4.30 (m, 1H, H2), 5.12 (br. s, 1H, NH), 7.31 (d, *J* = 8.0 Hz, 2H, H7), 7.76 (d, *J* = 8.0 Hz, 2H, H6). ^{13}**C NMR** (126 MHz, $CDCl_3$) δ 21.7 (C9), 26.5 (C3), 26.9 (C4), 51.2 (C1), 70.3 (C2), 127.3 (C6), 129.9 (C7), 137.4 (C8), 143.8 (C5). **HRMS** (ESI+) Found: 264.0666, $C_{11}H_{15}NO_3SNa$ $[M+Na]^+$ requires: 264.0665. **IR** ν_{max} (thin film)/cm^{-1} 3499br.w, 3279br.w, 1332m, 1159s, 1093m, 668m. $[\alpha]_D^{25}$ +1.96 (*c* = 0.51, $CHCl_3$). **MP** 101–103 °C. % *ee* not measured but see S8 for crystallographic data confirming absolute configuration.

N-Cyclobutyl-4-(hydroxymethyl)benzenesulfonamide 19[9]

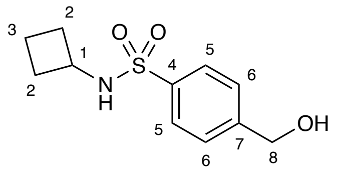

Prepared according to the general procedure, using variant R19/FI/LLV (3.40 mL of 149 µM concentration in phosphate buffer, overall concentration of 2.0 µM), glucose (25 mL of 1.0 M stock in phosphate buffer), GDH (5.0 ml of 2.0 U/µL stock in phosphate buffer), $NADP^+$ monosodium salt (5.0 ml of 4.0 mM stock in phosphate buffer) in phosphate buffer (211 mL) and substrate **12c** (1.0 mL of 0.5 M stock in DMSO, 0.50 mmol, overall concentration of 2.0 mM) stirring for 48 h. Purification by column chromatography (10% EtOAc in pentane) afforded the benzylic alcohol **19** as a clear, colorless oil (61.5 mg, 51%). $\mathbf{R}_f$ 0.26 (50% EtOAc in pentane). ^{1}**H NMR** (400 MHz, $CDCl_3$) δ 1.50–1.66 (m, 2H, H3), 1.70–1.82 (m, 2H, H2), 2.05–2.16 (m, 3H, H2,OH), 3.79 (app. sext, *J* = 8.0 Hz, H1), 4.78 (s, 2H, H8), 4.83 (d, *J* = 8.5 Hz, 1H, NH), 7.48 (d, *J* = 8.5 Hz, 2H, H6), 7.82 (d, *J* = 8.5 Hz, 2H, H5). **HRMS** (ESI+) Found: 264.0665, $C_{11}H_{15}NO_3SNa$ $[M+Na]^+$ requires: 264.0665.

N-[(1*S*,2*R*)-2-Hydroxycyclobutyl]propane-2-sulfonamide 22, *N*-[(1*S**,2*S**)-2-hydroxycyclobutyl]propane-2-sulfonamide 23, and *cis*- and *trans*-*N*-(3-hydroxycyclobutyl)propane-2-sulfonamide 24 and 25

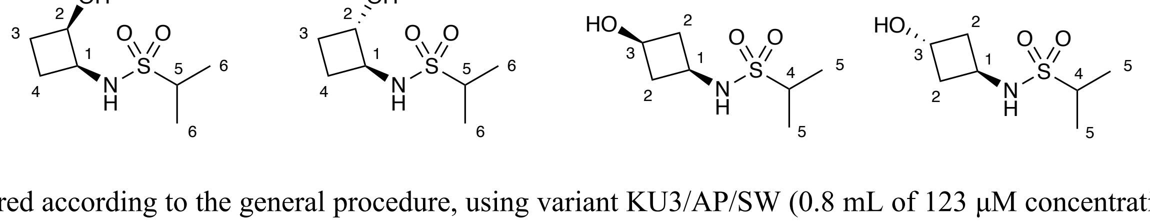

Prepared according to the general procedure, using variant KU3/AP/SW (0.8 mL of 123 µM concentration in phosphate buffer, overall concentration of 2.0 µM), glucose (10 mL of 1.0 M stock in phosphate buffer), GDH (2.0 ml of 2.0 U/µL stock in phosphate buffer), $NADP^+$ monosodium salt (2.0 ml of 4.0 mM stock in phosphate

buffer) in phosphate buffer (35 mL) and substrate **12c** (17.7 mg neat, 0.10 mmol, overall concentration of 2.0 mM) stirring for 48 h. Purification by column chromatography (10–50% EtOAc in pentane) afforded the 1,2-*cis* diastereomer **22** as a colorless oil (4.2 mg, 22%). $\mathbf{R}_f$ 0.12 (40% EtOAc in pentane). ^{1}**H NMR** (400 MHz, $CDCl_3$) δ 1.37 (d, J = 7.0 Hz, 3H, H6), 1.38 (d, J = 7.0 Hz, 3H, H6), 1.77–1.82 (m, 1H, H3), 2.06–2.16 (m, 2H, H3,4), 2.26–2.31 (m, 1H, H4), 3.14 (sept, J = 7.0 Hz, 1H, H5), 3.92–3.99 (m, 1H, H1), 4.45–4.49 (m, 1H, H2), 4.84 (br. d, J = 8.5 Hz, 1H, NH). ^{13}**C NMR** (101 MHz, $CDCl_3$) δ 16.6 and 16.8 (C6), 26.2 (C3), 28.0 (C4), 51.7 (C1), 54.0 (C5), 71.1 (C2). **HRMS** (ESI+) Found: 216.0666, $C_7H_{15}NO_3SNa$ $[M+Na]^+$ requires: 216.0665. **IR** ν_{max} (thin film)/cm^{-1} 3282br.w, 2981w, 2946w, 1269m, 1132s, 695m. $[\alpha]_D^{25}$ –12.7 (c = 0.4, $CHCl_3$). % *ee* not measured but see S6 for determination of absolute configuration by Moshers ester analysis.

Further elution yielded the 1,2-*trans* diastereomer **23** as a colorless oil (5.1 mg, 26% yield). $\mathbf{R}_f$ 0.08 (40% EtOAc in pentane). ^{1}**H NMR** (400 MHz, $CDCl_3$) δ 1.33–1.43 (m, 1H, H4) overlaying 1.38 (d, J = 6.5 Hz, 3H, H6), 1.39 (d, J = 6.5 Hz, 3H, H6), 1.54–1.59 (m, 1H, H3), 2.09–2.16 (m, 2H, H3,H), 3.22 (sept, J = 6.5 Hz, 1H, H5), 3.56 (quin, J = 8.0 Hz, 1H, H1), 4.00–4.06 (m, 1H, H2), 4.61 (d, J = 8.0 Hz, 1H, NH). ^{13}**C NMR** (101 MHz, $CDCl_3$) δ 16.6 and 16.8 (C6), 21.0 (C4), 24.8 (C3), 53.9 (C5), 57.7 (C1), 74.4 (C2). **HRMS** (ESI+) Found: 216.0666, $C_7H_{15}NO_3SNa$ $[M+Na]^+$ requires: 216.0665. **IR** ν_{max} (thin film)/cm^{-1} 3278br.w, 2921m, 2851w, 1307m, 1135s, 1095m, 1060m, 694m. $[\alpha]_D^{25}$ +8.16 (c = 0.49, $CHCl_3$). The Moshers ester synthesis was unsuccessful; therefore, the % *ee* and absolute configuration were not determined.

Further elution yielded a mixture of the 3-hydroxylated metabolites as a colorless oil (1.6 mg, 8.5%, 1.5:1, *cis*/*trans*). $\mathbf{R}_f$ 0.05 (50% EtOAc in pentane). ***Cis* diastereomer 24**: ^{1}**H NMR** (500 MHz, $CDCl_3$) δ 1.35 (d, J = 7.0 Hz, 6H, H5), 1.86–1.94 (m, 2H, H2), 2.79–2.86 (m, 2H, H2), 3.09 (sept, J = 7.0 Hz, 1H, H4), 3.46–3.57 (m, 1H, H1), 3.99–4.05 (m, 1H, H3), 4.29 (br. s, 1H, OH). ^{13}**C NMR** (126 MHz, $CDCl_3$) δ 16.7 (C5), 40.3 (C1), 43.0 (C2), 54.0 (C4), 60.5 (C3). ***Trans* diastereomer 25**: ^{1}**H NMR** (500 MHz, $CDCl_3$) δ 1.36 (d, J = 7.0 Hz, 6H, H5) 2.23–2.30 (m, 1H, H2), 2.36–2.42 (m, 1H, H2), 3.11 (sept, J = 7.0 Hz, 1H, H4), 4.20–4.29 (m, 1H, H1), 4.48 (m, 1H, H3). ^{13}**C NMR** (126 MHz, $CDCl_3$) δ 16.7 (C5), 41.4 (C2), 46.0 (C1), 53.8 (C4), 64.4 (C3). **HRMS** (ESI–) Found: 192.0690, $C_7H_{14}NO_3S$ $[M–H]^-$ requires: 192.0700. **IR** ν_{max} (thin film)/cm^{-1} 3458br.w, 3272br.w, 2983w, 2936w, 1307m, 1133s, 1056m.

***tert*-Butyl [(2*R*)-2-hydroxybicyclo[1.1.1]pent-1-yl]carbamate 26**

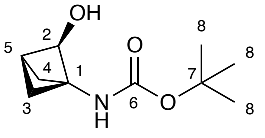

Prepared according to the general procedure, using variant GV/AI (6.0 mL of 50 μM concentration in phosphate buffer, overall concentration of 2.0 μM), glucose (15 mL of 1.0 M stock in phosphate buffer), GDH (30 mL of 2.0 U/μL stock in phosphate buffer), $NADP^+$ monosodium salt (3.0 ml of 4.0 mM stock in phosphate buffer) in phosphate buffer (122 mL) and substrate **13a** (1.5 mL of 0.1 M stock in ethanol, 0.15 mmol, overall

concentration of 1.0 mM) stirring for 42 h. Purification by column chromatography (0–35% EtOAc in pentane) afforded the title compound as a colorless solid (7.6 mg, 25%). **R*f*** 0.16 (30% EtOAc in pentane). **^{1}H NMR** (400 MHz, $CDCl_3$) δ 1.39 (dd, *J* = 9.5, 2.5 Hz, 1H, H4), 1.42 (s, 9H, H8), 1.74 (d, *J* = 2.5 Hz, 1H, H4), 1.87 (dd, *J* = 6.0, 2.0 Hz, 1H, H3), 2.41 (s, 1H, H5), 2.99 (br. d, *J* = 9.5 Hz, 1H, H3), 4.17 (br. dd, *J* = 6.0, 1.5 Hz, 1H, H2), 4.95 (br. s, 1H) and 5.19 (br. s, 1H, OH,NH). **^{13}C NMR** (101 MHz, $CDCl_3$) δ 28.4 (C8), 29.8 (C5), 41.6 (C4), 47.5 (C3), 53.3 (C1), 80.5 (C7), 84.6 (C2), 156.6 (C6). **HRMS** (ESI+) Found: 222.1103, $C_{10}H_{17}NO_3Na$ $[M+Na]^+$ requires: 222.1101. **IR** ν_{max} (thin film)/cm^{-1} 3317br.w, 2978w, 1683s, 1515m, 1367m, 1281m, 1168s, 1122m, 1064m. **MP** 87–90 °C. **$[\alpha]_D^{25}$** −6.16 (*c* = 0.76, $CHCl_3$). See S4 for GC traces showing % *ee* and S8 for crystallographic data confirming absolute configuration.

1-Hydroxy-2-methylprop-2-yl bicyclo[1.1.1]pent-1-ylcarbamate 27 and (±)-3-(bicyclo[1.1.1]pent-1-yl)-4-hydroxy-5,5-dimethyloxazolidin-2-one 28

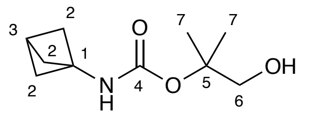

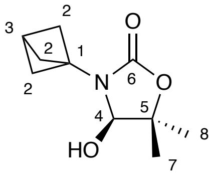

Prepared according to the general procedure, using variant R19/FI/AP (4.0 mL of 50 μM concentration in phosphate buffer, overall concentration of 2.0 μM), glucose (10 mL of 1.0 M stock in phosphate buffer), GDH (2.0 ml of 2.0 U/μL stock in phosphate buffer), $NADP^+$ monosodium salt (2.0 ml of 4.0 mM stock in phosphate buffer) in phosphate buffer (81 mL) and substrate **13a** (1.0 mL of 0.1 M stock in DMSO, 0.1 mmol, overall concentration of 1.0 mM) stirring for 48 h. Purification by column chromatography (0–50% EtOAc in pentane) afforded the title compound **27** as a colorless oil (4.1 mg, 21%). **R*f*** 0.08 (30% EtOAc in pentane). **^{1}H NMR** (400 MHz, $CDCl_3$) – resonances show substantial rotameric broadening – δ 1.38 (br. s, 6H, H7), 2.02 (br. s, 6H, H2), 2.41 (br. s, 1H, H3), 3.63 (br. s, 2H, H6), 4.26 (br. s, 1H) and 5.14 (br. s, 1H, OH,NH). **^{13}C NMR** (101 MHz, $CDCl_3$) δ 23.9 (C7), 24.2 (C3), 48.6 (C1), 52.6 (C2), 70.1 (C6), 77.4 (C5), 156.2 (C4). **HRMS** (ESI+) Found: 222.1102, $C_{10}H_{17}NO_3Na$ [M+Na]+ requires: 222.1101. **IR** ν_{max} (thin film)/cm^{-1} 3317br.w, 2977m, 2916w, 2878w, 1688s, 1520m, 1285m, 1204m, 1144m, 1056m.

Oxazolidinone **28** was also isolated, as a colorless crystalline solid (1.7 mg, 9%). **R*f*** 0.30 (60% EtOAc in pentane). **^{1}H NMR** (400 MHz, $CDCl_3$) δ 1.37 (s, 3H) and 1.40 (s, 3H, H7,8), 2.15 (dd, *J* = 9.5, 1.5 Hz, 3H) and 2.21 (dd, *J* = 9.5, 1.5 Hz, 3H, H2), 2.29 (d, *J* = 9.0 Hz, 1H, OH), 2.51 (s, 1H, H3), 4.79 (d, *J* = 9.0 Hz, 1H, H4). **^{13}C NMR** (151 MHz, $CDCl_3$) δ 20.9 (C7 or C8), 24.7 (C3), 26.5 (C7 or C8), 49.3 (C1), 52.6 (C2), 81.5 (C5), 86.4 (C4), 155.1 (C6). **HRMS** (APCI+) Found 198.1128, $C_{10}H_{16}O_3N$ $[M+H]^+$ requires: 198.1125. **IR** ν_{max} (thin film)/cm^{-1} 3343br.w, 2920s, 2851m, 1725s, 1419m, 1284w. **MP** 93–96 °C.

***tert*-Butyl [(2*S*)-2-hydroxybicyclo[1.1.1]pent-1-yl]carbamate 26**

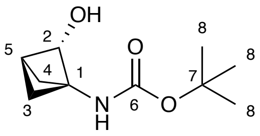

Substrate **13a** (27.5 mg, 0.150 mmol, as a solution in 750 μL methanol, overall concentration of 1.0 mM) was added to a solution of variant K19/FV/IG/AF (3.0 mL of 100 μM concentration in phosphate buffer, overall concentration of 2.0 μM), glucose (15 mL of 1.0 M stock in phosphate buffer), GDH (1.5 mL of 2.5 U/μL stock in phosphate buffer), $NADP^+$ monosodium salt (1.5 mL of 40 mM stock in phosphate buffer) in phosphate buffer (129 mL). The mixture was stirred at 120 rpm at 20 °C for 18 h then extracted with ethyl acetate (4 × 100 mL), separating the biphasic gel by centrifuge as necessary. The combined organic layers were dried ($MgSO_4$), filtered, and concentrated under vacuum. Purification of the crude residue by column chromatography (10–20% EtOAc in petroleum ether) afforded the title compound as a colorless crystalline solid (3.0 mg, 10%, *ee* = 82% in favor of the 2*S*-enantiomer). $[\alpha]_D^{25}$ +10.4 (*c* = 0.048, $CHCl_3$); other data as for the 2*R*-enantiomer. See S4 for GC traces showing % *ee*.

Benzyl [(2*R*)-2-hydroxybicyclo[1.1.1]pent-1-yl]carbamate 29

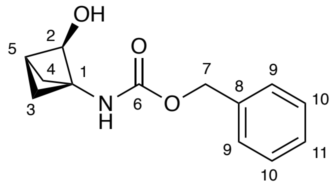

Prepared according to the general procedure, using variant K19/FV/QP (1.2 mL of 164 μM concentration in phosphate buffer, overall concentration of 2.0 μM), glucose (10 mL of 1.0 M stock in phosphate buffer), GDH (2.0 ml of 2.0 U/μL stock in phosphate buffer), $NADP^+$ monosodium salt (2.0 ml of 4.0 mM stock in phosphate buffer) in phosphate buffer (84 mL) and substrate **13d** (1.0 mL of 0.1 M stock in DMSO, 0.1 mmol, overall concentration of 1.0 mM) stirring for 4 h. Purification by column chromatography (10–20% EtOAc in pentane) afforded the title compound as a yellow oil (4.8 mg, contaminated with 2-(hydroxymethyl)phenol and a second benzyl-containing impurity as shown in S7;* yield not quantifiable). $\mathbf{R}_f$ 0.59 (80% EtOAc in pentane). **^{1}H NMR** (400 MHz, $CDCl_3$) δ 1.44 (dd, *J* = 9.5, 2.5 Hz, 1H) and 1.78 (d, *J* = 2.5 Hz, 1H, H4), 1.92 (dd, *J* = 6.0, 2.0 Hz, 1H, H3), 2.45 (s, 1H, H5), 3.02 (d, *J* = 9.5 Hz, 1H, H4), 4.23 (d, *J* = 6.0 Hz, 1H, H2), 4.58 (br. s, 1H, OH), 5.07 (d, *J* = 12.0 Hz, 1H) and 5.10 (d, *J* = 12.0 Hz, 1H, H7), 5.30 (br. s, 1H, NH), 7.32–7.38 (m, 5H, H9,10,11). **^{13}C NMR** (101 MHz, $CDCl_3$) δ 29.8 (C5), 41.7 (C4), 47.6 (C3), 53.2 (C1), 67.3 (C7), 84.6 (C2), 128.4, 128.5, and 128.8 (C9,10,11), 136.1 (C8), 156.9 (C6). **HRMS** (ESI+) Found: 256.0945, $C_{13}H_{15}NO_3Na$ $[M+Na]^+$ requires: 256.0944. **IR** ν_{max} (thin film)/cm^{-1} 3336br.w, 2924w, 1700s, 1521m, 1456m, 1402m,

1339m, 1261m, 1207m, 1061m, 697m. $[\alpha]_D^{25}$ −5.29 (*c* = 1.53, $CHCl_3$). See S4 for GC traces showing % *ee* and S6 for determination of absolute configuration by chemical correlation.

* Diagnostic resonances in the NMR spectra (400/101 MHz, $CDCl_3$) are attributable to 2-(hydroxymethyl)phenol [δ_H 4.88 (s, 2H), 6.85 (td, *J* = 7.5, 1.5 Hz, 1H), 6.89 (dd, *J* = 7.5, 1.5 Hz, 1H), 7.04 (dd, *J* = 7.5, 1.5 Hz, 1H), 7.20 (td, *J* = 7.5, 1.5 Hz, 1H); δ_C 64.9, 116.8, 120.2, 127.9, 129.7, 156.4 – one resonance not resolved]; a second benzyl-containing impurity is assigned tentatively as benzyl carbamate [δ_H 5.10 (s, 2H); δ_C 67.1 (CH_2)].

S5.3 Reaction optimization and preparative scale hydroxylations

S5.3.1 Effect of aeration during reaction

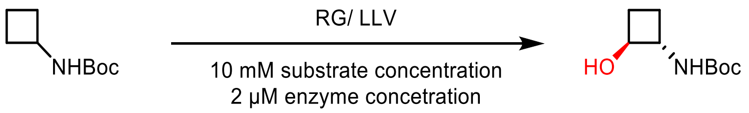

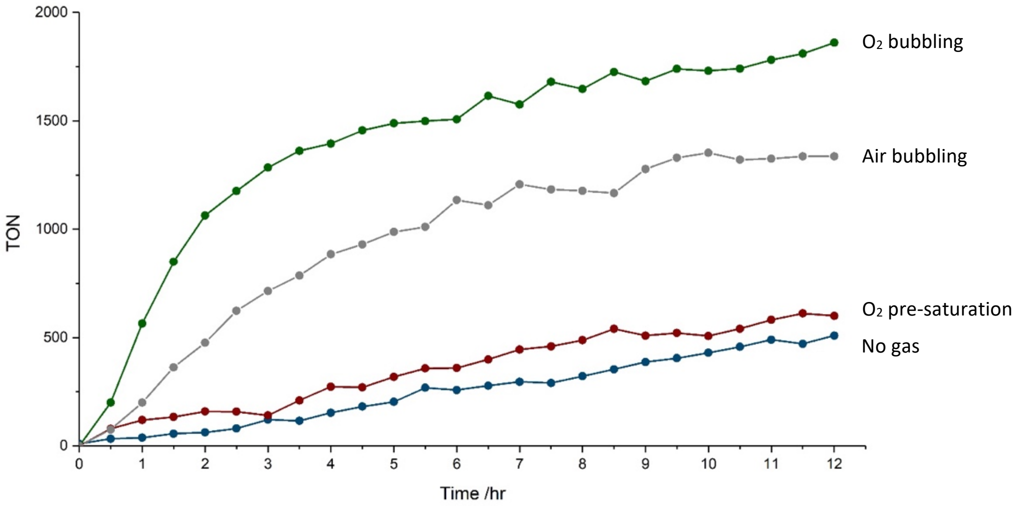

Reaction conditions: A solution of *tert*-butyl cyclobutylcarbamate (**12a**, 171 mg, 1.0 mmol) in DMSO (2.0 mL, 10 mM overall substrate concentration) was added to a stirred solution of P450$_{BM3}$ RG/LLV (3.0 mL of 65 µM stock in phosphate buffer), GDH (80 mg), NADP$^+$ monosodium salt (30 mg) and glucose (9.0 g) in phosphate buffer (97 mL). The reaction mixture was stirred and air (or oxygen) was bubbled into the reaction medium (Image S1, below) at a rate of approximately three bubbles/s. Every 30 min, a 50 µL aliquot was transferred to a 1.5 mL microcentrifuge tube and ethyl acetate (300 µL) was added; the mixture was vortexed for 30 s and then centrifuged at 13,300 *g* for 1 min after which the organic layer was transferred into GC vials for GC analysis.

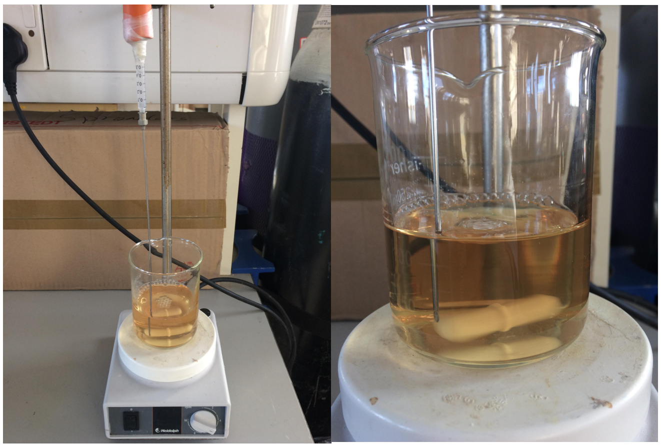

Image S1 – Reaction set-up for preparative scale biocatalytic transformation of 1.0 mmol *tert*-butyl cyclobutylcarbamate (**12a**) with gas bubbling into 100 mL reaction volume.

S5.3.2 Effect of substrate stock solvent

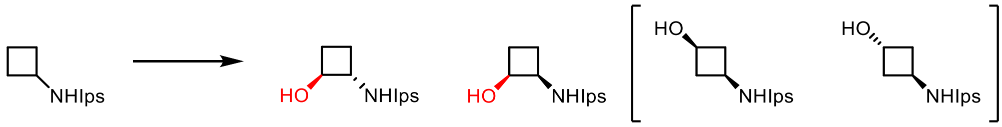

Reaction conditions: Substrate **12c** (17.7 mg, 0.10 mmol) was added either neat or as a solution in the specified solvent (1.0 mL, 2.0 mM final substrate concentration) to a stirred solution of $P450_{BM3}$ KU3/AP/SW (0.8 mL of 123 µM stock in phosphate buffer), GDH (2.0 ml of 2.0 U/µL stock in phosphate buffer), $NADP^+$ monosodium salt (2.0 ml of 4.0 mM stock in phosphate buffer) and glucose (10 mL of 1.0 M stock in phosphate buffer) in phosphate buffer (34 mL, or 35 mL when substrate added neat). After 48 h, a 50 µL aliquot was transferred to a 1.5 mL microcentrifuge tube and ethyl acetate (300 µL) was added; the mixture was vortexed for 30 s and then centrifuged at 13,300 *g* for 1 min after which the organic layer was transferred into GC vials for GC analysis.

Entry	Solvent used for substrate	GC conversion
**1**	Ethanol	30%
**2**	DMSO	45%
**3**	Neat (no solvent)	83%

S5.3.3 Hydroxylation of *tert*-butyl cyclobutylcarbamate 12a (20 mmol)

See Image S2, below. A solution of *tert*-butyl cyclobutylcarbamate (**12a**, 3.42 g, 20.0 mmol) in DMSO (40 mL, 10 mM overall substrate concentration) was added to a stirred solution of $P450_{BM3}$ variant RG/LLV (71 mL of 56 µM stock in phosphate buffer, overall concentration of 2.0 µM), GDH (1.6 g), $NADP^+$ monosodium salt (600 mg) and glucose (180 g) in phosphate buffer (1890 mL). The reaction mixture was stirred with O_2 bubbling into the reaction medium at a rate of approximately three bubbles/s. The pH was monitored throughout the reaction and maintained at pH 7.9 by periodic addition of aq. 1.0 M KOH solution. After 3.5 days ethyl acetate (500 mL) was added to quench the reaction and the organic layer was separated. The aqueous layer was then concentrated *in vacuo* to approximately 400 mL, after which it was saturated with solid NaCl and portioned into 4 × 250 mL centrifuge bottles. The bottles were then shaken for 30 seconds, centrifuged at 9500 *g* for 4 min after which the organic layer was removed and the extraction process was repeated (×5). The combined organic extracts were dried ($MgSO_4$), filtered, and concentrated *in vacuo*. The residue was purified by column chromatography (20–50% EtOAc in pentane) to yield, as major product, *tert*-butyl [(1*S*,2*S*)-2-hydroxycyclobutyl]carbamate **14** as a colorless solid (1.81 g, 48%). Data as above. See S4 for GC traces showing % *ee*.

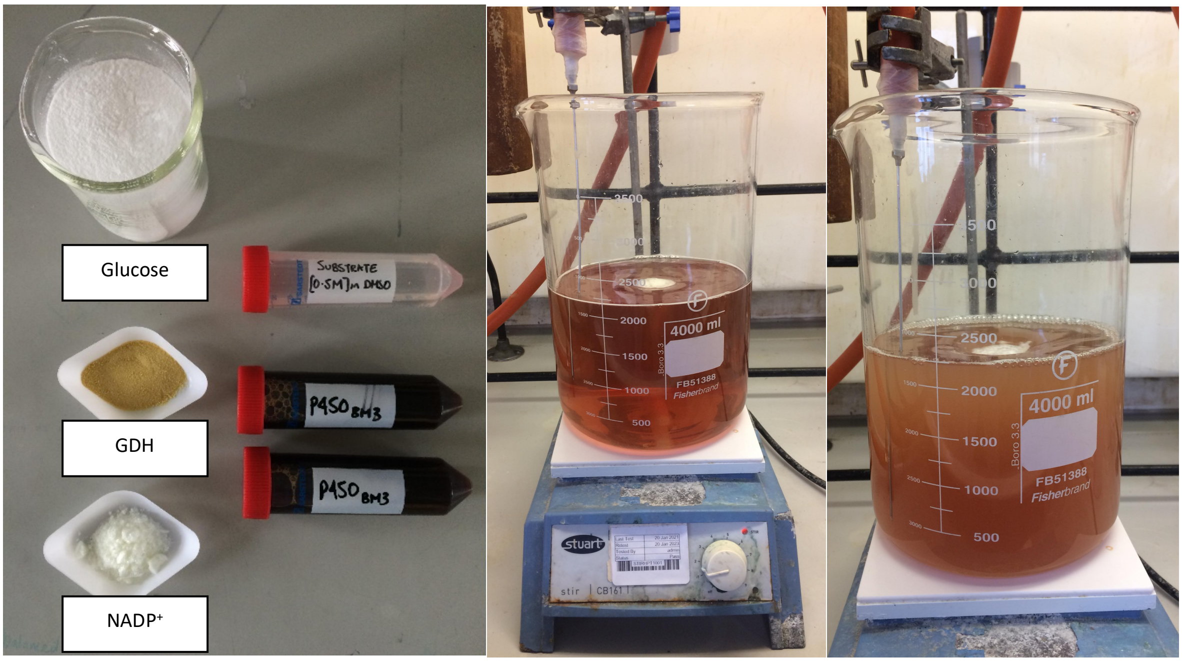

Image S2 – Left: Reaction components required for large-scale biotransformation (enzyme used in reaction immediately after purification); Middle and Right: Reaction mixture before and after addition of substrate.

S5.3.4 Hydroxylation of *tert*-butyl bicyclo[1.1.1]pent-1-ylcarbamate 13a (1.5 mmol)

This was carried out according to the general procedure (S5.2), using variant GV/AI (24 mL of 126 µM concentration in phosphate buffer, overall concentration of 6.0 µM), glucose (18 g), GDH (160 mg), $NADP^+$ monosodium salt (60 mg) in phosphate buffer (471 mL) and substrate **13a** (5.0 mL of 0.3 M stock in DMSO, 1.5 mmol, overall concentration of 3.0 mM) stirring for 48 h. Purification by column chromatography (10–40% EtOAc in pentane) afforded *tert*-butyl [(2*R*)-2-hydroxybicyclo[1.1.1]pent-1-yl]carbamate **26** as a colorless crystalline solid (132 mg, 44%; 51% brsm). Data as above. See S4 for GC traces showing % *ee.*

S5.3.5 Hydroxylation of benzyl bicyclo[1.1.1]pent-1-ylcarbamate 13d (1.4 mmol)

This was carried out according to the general procedure (S5.2) using variant K19/FV/QP (21 mL of 143 µM concentration in phosphate buffer, overall concentration of 6.0 µM), glucose (18 g), GDH (160 mg), $NADP^+$ monosodium salt (60 mg) in phosphate buffer (474 mL) and substrate **13d** (4.6 mL of 0.3 M stock in DMSO, 1.38 mmol, overall concentration of 3.0 mM) stirring for 48 h. Purification by column chromatography (30–60% EtOAc in pentane) afforded benzyl [(2*R*)-hydroxybicyclo[1.1.1]pent-1-yl]carbamate **29** as a yellow oil (74.2 mg, 23%; 39% brsm). Data as above. See S4 for GC traces showing % *ee*.

S5.4 Metabolite derivatization

N-[(*1S,2S*)-2-Hydroxycyclobutyl]-2-iodobenzamide 30

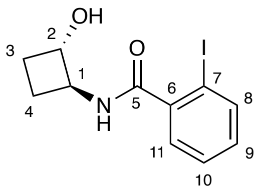

Hydrochloric acid (100 µL, conc.) was added to a stirred solution of *tert*-butyl [(1*S*,2*S*)-2-hydroxycyclobutyl]carbamate **14** (20.0 mg, 0.107 mmol) in methanol (500 µL) and the mixture was stirred at RT for 2 h. Methanol was removed *in vacuo* and the crude material was redissolved in acetonitrile (1 mL). To this solution was added DIPEA (61 µL, 0.35 mmol), HATU (49.0 mg, 0.129 mmol) and 2-iodobenzoic acid (32 mg, 0.129 mmol) and the mixture was stirred for 16 h. After this time, the reaction was quenched with hydrochloric acid (1.0 mL, 1.0 M) and extracted with ethyl acetate (3 × 2 mL); the combined extracts were dried ($MgSO_4$), filtered, and concentrated *in vacuo*. The residue was purified by column chromatography (40–50% EtOAc in pentane) to yield the title compound as a yellow oil (31.2 mg, 92%). **R*f*** 0.22 (60% EtOAc in pentane). **^{1}H NMR** (400 MHz, $CDCl_3$) δ 1.43–1.53 (m, 1H, H3 or H4), 1.73–1.81 (m, 1H, H3 or H4), 2.14–2.25 (m, 2H, H3,4), 3.43 (br. s, 1H, NH), 4.00–4.07 (m, 1H, H1), 4.20 (app. q, *J* = 8.0 Hz, 1H, H2), 6.05 (br. s, 1H, OH), 7.11 (ddd, *J* = 8.0, 7.5, 2.0 Hz, 1H, H9), 7.38 (td, *J* = 7.5, 1.0 Hz, H10), 7.42 (dd, *J* = 7.5, 2.0 Hz, 1H, H11), 7.87 (dd, *J* = 8.0, 1.0 Hz, 1H, H8). **^{13}C NMR** (126 MHz, $CDCl_3$) δ 18.8 (C4), 25.1 (C3), 55.8 (C1), 73.2 (C2), 92.6 (C7), 128.4 and 128.5 (C10,11), 131.5 (C9), 140.1 (C8), 141.6 (C6), 170.2 (C5). **HRMS** (ESI+) Found: 317.9987, $C_{11}H_{12}INO_2Na$ $[M+H]^+$ requires: 317.9984. **IR** ν_{max} (thin film)/cm^{-1} 3265 br.w, 2917w, 1646m, 1540m, 846m. **$[\alpha]_D^{25}$** +0.61 (*c* = 1.20, $CHCl_3$).

tert-Butyl (*S*)-(2-oxocyclobutyl)carbamate 31

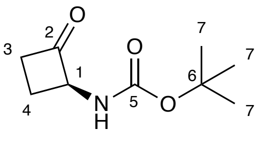

Dess–Martin periodinane (136 mg, 0.321 mmol) was added to a stirred solution of *tert*-butyl [(1*S*,2*S*)-2-hydroxycyclobutyl]carbamate **14** (40.0 mg, 0.214 mmol) in dichloromethane (2 mL). The mixture was stirred at RT for 20 h, quenched with sat. aq. $NaHCO_3$ solution (5 mL) and extracted with ethyl acetate (3 × 10 mL). The combined organic extracts were dried ($MgSO_4$), filtered, and concentrated *in vacuo* to give a residue that was purified by column chromatography (20–40% EtOAc in pentane), affording the title compound as a white powder (31.5 mg, 81%). **R*f*** 0.53 (50% EtOAc in pentane). **^{1}H NMR** (400 MHz, $CDCl_3$) δ 1.42 (s, 9H, H7), 1.94–2.03 (m, 1H, H4), 2.40–2.44 (m, 1H, H4), 2.81–2.90 (m, 2H, H3), 4.82 (q, *J* = 8.5 Hz, 1H, H1), 5.10 (br. s, 1H, NH). **^{13}C NMR** (101 MHz, $CDCl_3$) δ 20.1 (C4), 28.4 (C7), 41.6 (C3), 65.2 (C1), 80.4 (C6), 154.8 (C5), 205.9 (C2). **HRMS** (ESI+) Found: 208.0947, $C_9H_{15}NO_3Na$ $[M+Na]^+$ requires: 208.0944. **IR** ν_{max} (thin

film)/cm^{-1} 3349br.w, 2978w, 2935w, 1791s, 1693s, 1515m, 1330m, 1254m, 1162s, 992m. **MP** 72–75 °C. **[α]$_D^{25}$** –1.98 (*c* = 1.02, $CHCl_3$).

(1*S*,2*S*)-[2-(*tert*-Butoxycarbonyl)amino]cyclobutyl 4-(*N*,*N*-dipropylsulfamoyl)benzoate 32

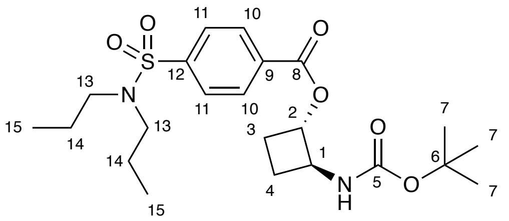

N,*N*′-Dicyclohexylcarbodiimide (18.0 mg, 0.0872 mmol) was added to a stirred solution of *tert*-butyl [(1*S*,2*S*)-2-hydroxycyclobutyl]carbamate **14** (11.0 mg, 0.0587 mmol), probenecid (25.0 mg, 0.0876 mmol) and DMAP (2.10 mg, 0.017 mmol) in dry dichloromethane (0.5 mL) at 0 °C under argon. The mixture was stirred at 0 °C for 30 min and then at RT for 14 h. The mixture was then filtered through Celite, washed with sat. aq. $NaHCO_3$ solution (1 mL), and the aqueous extracted with ethyl acetate (3 × 2 mL); the combined extracts were dried ($MgSO_4$), filtered, and concentrated *in vacuo*. The crude product was purified by column chromatography (0–30% EtOAc in pentane) to yield the title compound as a white powder (19.9 mg, 75%). **R*f*** 0.63 (50% EtOAc in pentane). **^{1}H NMR** (600 MHz, $CDCl_3$) δ 0.86 (t, *J* = 7.4 Hz, 6H, H15), 1.43 (s, 9H, H7), 1.51–1.56 (m, 4H, H14), 1.56–1.61 (m, 1H, H4), 1.79–1.89 (m, 1H, H3), 2.25–2.36 (m, 2H, H3,4), 3.08–3.11 (m, 4H, H13), 4.11–3.21 (m, 1H, H1), 5.04 (app. q, *J* = 7.5 Hz, 1H, H2), 5.11 (br. s, 1H, NH), 7.87 (d, *J* = 8.5 Hz, 2H, H11), 8.15 (d, *J* = 8.5 Hz, 2H, H10). **^{13}C NMR** (101 MHz, $CDCl_3$) δ 11.3 (C15), 22.0 (C14), 28.5 (C7), 29.9 (C3,4), 50.0 (C13), 52.8 (C1), 74.4 (C2), 79.8 (C6), 127.1 (C11), 130.5 (C10), 133.3 (C9), 144.6 (C12), 155.0 (C5), 165.0 (C8). **HRMS** (ESI+) Found: 477.2029, $C_{22}H_{34}NO_6SNa$ $[M+Na]^+$ requires: 477.2030. **IR** ν_{max} (thin film)/cm^{-1} 3381w, 2924m, 1718s, 1343m, 1276s, 1162s, 1106m, 996m. **MP** 92–94 °C. **[α]$_D^{25}$** +40.6 (*c* = 1.22, $CHCl_3$).

***N*-{(*trans*)-3-[(5-Bromopyridin-2-yl)oxy]cyclobutyl}-4-methylbenzenesulfonamide 33**

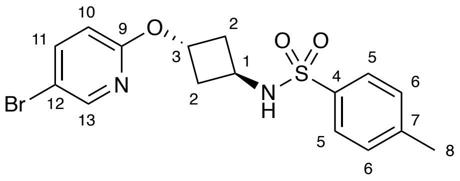

NaH (6.5 mg, 0.16 mmol, 60% in mineral oil) was added to a stirred solution of *N*-[(*trans*)-3-hydroxycyclobutyl]-4-methylbenzenesulfonamide **18** (20.0 mg, 0.0829 mmol) in DMF (0.5 mL) at RT. After 10 min, 5-bromo-2-fluoropyridine (8.5 μL, 0.083 mmol) was added and the mixture was stirred at RT for a further 6 h and then at 90 °C for 18 h. The mixture was then cooled to RT, diluted with water (1 mL), extracted with ethyl acetate (3 x 1 mL), and the combined extracts were dried ($MgSO_4$), filtered, and concentrated *in vacuo*. The crude product was purified by preparative TLC (50% EtOAc in pentane) to yield the title compound

as a yellow oil (15.9 mg, 48%, 61% brsm). **R_f** 0.7 (50% EtOAc in pentane). **^{1}H NMR** (600 MHz, $CDCl_3$) δ 2.29–2.34 (m, 2H, H2), 2.35–2.40 (m, 2H, H2), 2.42 (s, 3H, H8), 4.01–4.07 (m, 1H, H1), 4.92 (d, *J* = 7.5 Hz, 1H, NH), 5.15 (tt, *J* = 7.0, 3.5 Hz, 1H, H3), 6.59 (d, *J* = 9.0 Hz, 1H, H10), 7.30 (d, *J* = 8.0 Hz, 2H, H6), 7.62 (dd, *J* = 9.0, 2.5 Hz, 1H, H11), 7.75 (d, *J* = 8.0 Hz, 2H, H5), 8.10 (d, *J* = 2.5 Hz, 1H, H13). **^{13}C NMR** (151 MHz, $CDCl_3$) δ 21.7 (C8), 38.0 (C2), 45.5 (C1), 68.5 (C3), 112.1 (C12), 112.9 (C10), 127.2 (C5), 129.9 (C6), 137.4 (C7), 141.4 (C11), 143.8 (C4), 147.8 (C13), 161.8 (C9). **HRMS** (ESI+) Found: 399.0202, $C_{16}H_{18}BrN_2O_3S$ $[M+H]^+$ requires 399.0195. **IR** ν_{max} (thin film)/cm^{-1} 3267w, 1483s, 1341m, 1280s, 1160s,1139m, 1091m, 666m.

S5.5 Synthesis of racemic samples and other compounds used for establishing *ee*

tert-Butyl (*R*)-(2-oxocyclobutyl)carbamate (*R*)-31

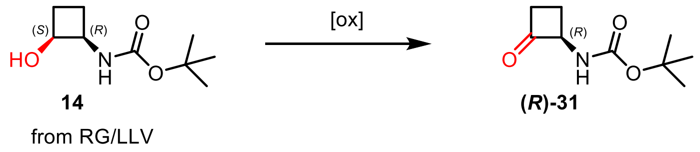

Dess–Martin periodinane (374 mg, 0.882 mmol) was added to a stirred solution of carbamate **16** (110 mg, 0.587 mmol) in dichloromethane (7 mL). The mixture was stirred at RT for 24 h, quenched with sat. aq. $NaHCO_3$ solution (20 mL) and extracted with ethyl acetate (3 × 30 mL). The combined organic extracts were dried ($MgSO_4$), filtered, and concentrated *in vacuo* to give a residue that was purified by column chromatography (10–30% EtOAc in pentane), affording cyclobutanone (*R*)-**31** (37.1 mg, 34%). The spectroscopic data match those for (*S*)-cyclobutanone **31**.

(±)-*tert*-Butyl-(2-oxocyclobutyl)carbamate 31

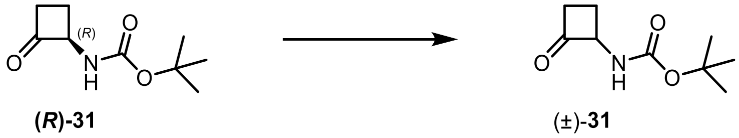

To a stirred solution of (*R*)-**31** (10.0 mg, 0.054 mmol) in ethanol (0.3 mL) was added Cs_2CO_3 (17.0 mg, 0.052 mmol) and stirring was continued at RT for 18 h. The reaction was neutralised with dilute hydrochloric acid (1.0 M) and extracted with ethyl acetate (3 × 1 mL); the organic extracts were dried ($MgSO_4$), filtered, and concentrated *in vacuo*. The crude product (7.0 mg, 70%) was used for GC analysis without further purification.

(±)-*trans*- and *cis*-*tert*-Butyl [2-hydroxycyclobutyl]carbamate 14 and 16

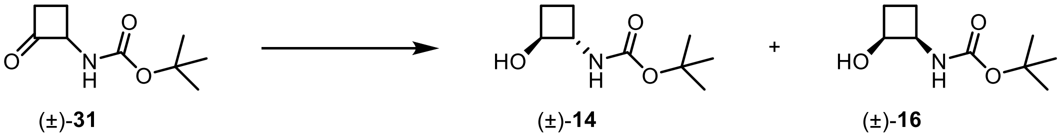

To a stirred solution of (±)-**31** (~7 mg crude product from the previous procedure, ~0.04 mmol) in methanol (0.2 mL) at 0 °C under N_2 was added $NaBH_4$ (2.0 mg, 0.053 mmol). The reaction mixture was warmed to RT, stirred for 16 h, then quenched with water and extracted with ethyl acetate (3 × 1 mL); the combined extracts were filtered through a pipette of $MgSO_4$ and concentrated *in vacuo*. The crude mixture was purified by preparative TLC (30% EtOAc in pentane), to yield small samples of (±)-**14** and (±)-**16** which were used as racemic standards for GC correlations with enantioenriched samples.

Benzyl [(2*R*)-hydroxybicyclo[1.1.1]pent-1-yl]carbamate 29

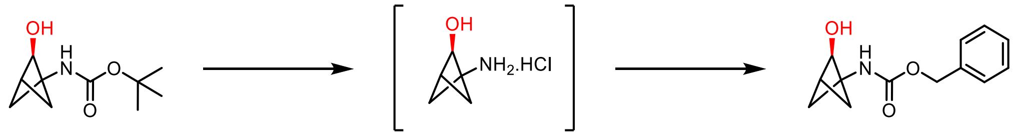

26
absolute configuration
known from X-Ray crystal structure

29

Hydrochloric acid (100 µL, conc.) was added to a stirred solution of carbamate **26** (10.0 mg, 0.0502 mmol) in dichloromethane (1 mL). After 2 h, the solvent was removed *in vacuo*, the residue was redissolved in dichloromethane (1 mL), and triethylamine (45 µL, 0.32 mmol) and benzyl chloroformate (20 µL, 0.13 mmol) were added at 0 °C under Ar. The cold bath was removed and the mixture was stirred at RT for 17 h. The mixture was concentrated *in vacuo* and the residue purified by column chromatography (10–50% EtOAc in pentane) to yield the Cbz-protected BCPA (*R*)-**29** as a white powder (3.1 mg, 27%). The spectroscopic data match those for **29** prepared from the enzymatic hydroxylation of substrate **13d** with K19/FV/QP.

S6 Determination of stereochemical configuration

S6.1 NOESY correlations for the assigned *cis* and *trans* hydroxycyclobutylamines **16** and **14**; the structure of *trans*-**14** was confirmed by single crystal X-Ray data (see S8 for details).

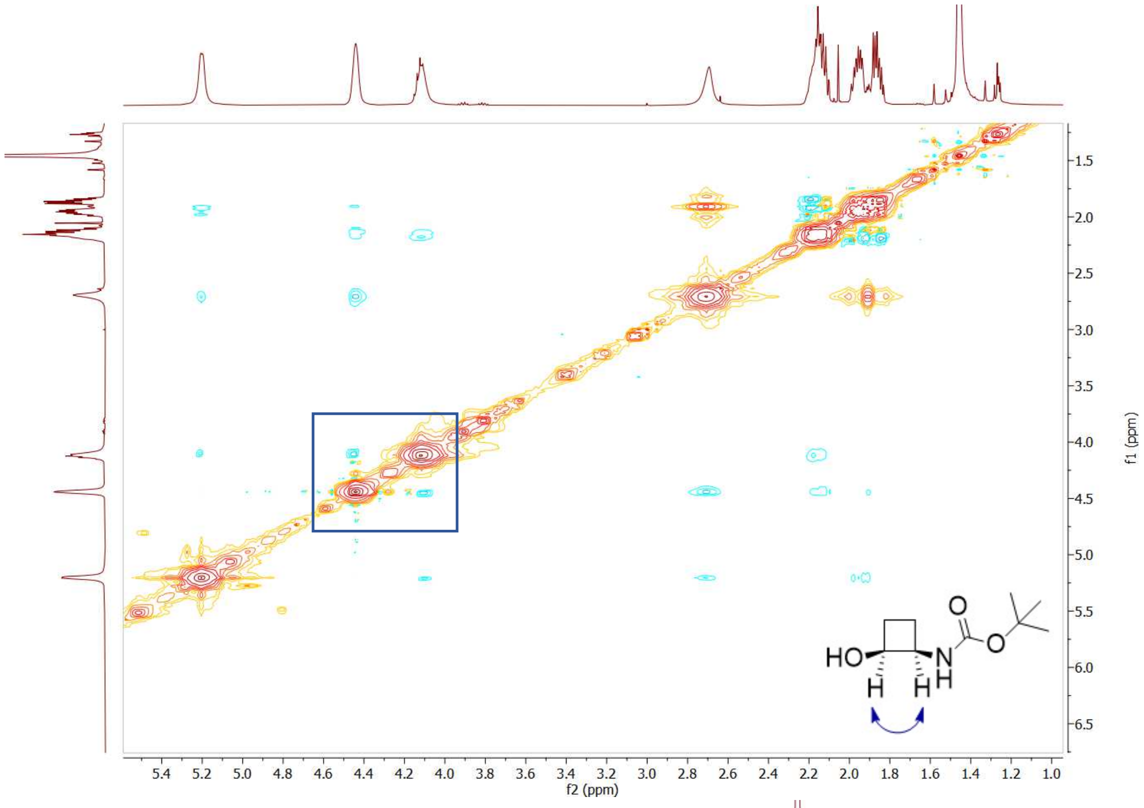

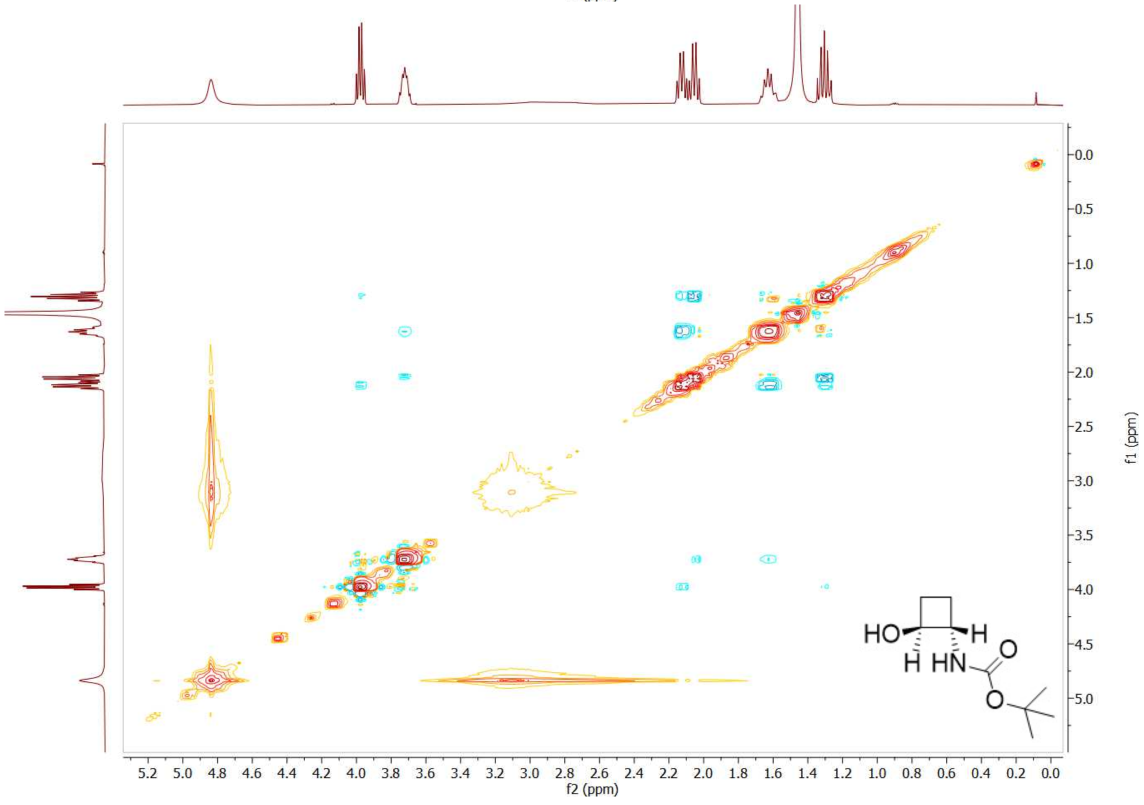

Figure S6.1 – Expanded region of NOESY spectrum of *cis*-**16** (top) and *trans*-**14** (bottom).

S6.2 The absolute configuration of *cis*-hydroxycyclobutylamine **16** from RP/HL/IG/AI was assigned by comparing the chiral GC data for derived ketone (*R*)-**31** with the data for the ketone (*S*)-**31** derived from the *trans* diastereomer **14** (whose absolute configuration was, in turn, established from crystallographic data).

Column: Cyclosil-B [Length: 30 m, Diameter: 0.25 mm, Film thickness: 0.25 µm]

Ramp: t = 0 min: 90 °C; 0.5 °C/min between 90–140 °C; 140 °C hold = 10 min; 10 °C/min between 140–200 °C; 200 °C hold = 1 min.

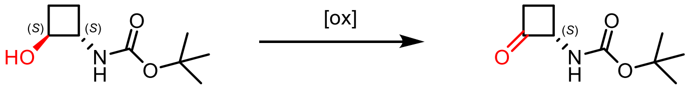

14

absolute configuration known from X-Ray

(*S*)-31

Procedure for oxidation of **14** described in **S5.4**.

Chromatogram

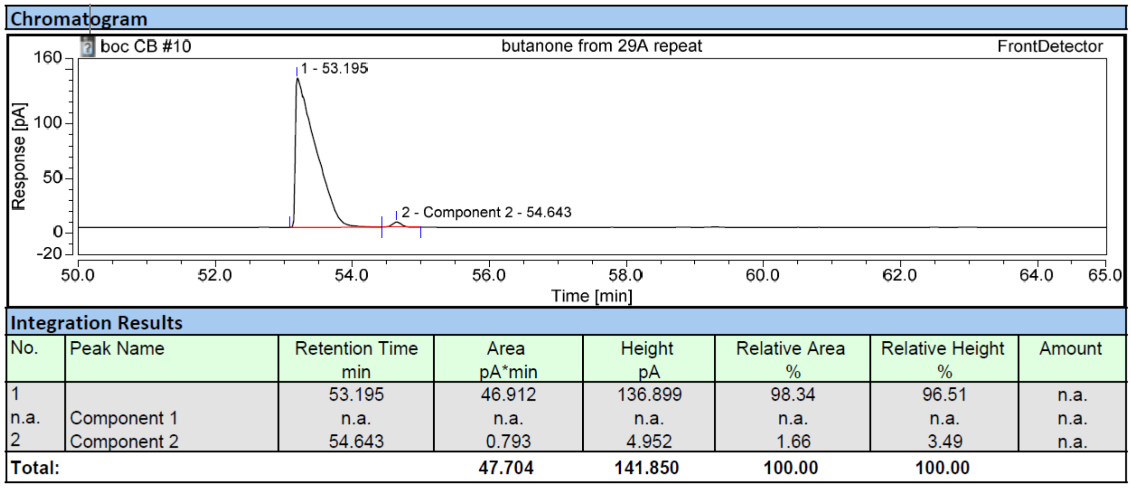

Integration Results

No.	Peak Name	Retention Time min	Area pA*min	Height pA	Relative Area %	Relative Height %	Amount
1		53.195	46.912	136.899	98.34	96.51	n.a.
n.a.	Component 1	n.a.	n.a.	n.a.	n.a.	n.a.	n.a.
2	Component 2	54.643	0.793	4.952	1.66	3.49	n.a.
**Total:**			**47.704**	**141.850**	**100.00**	**100.00**	

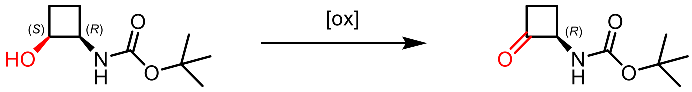

16

from RG/LLV

(*R*)-31

Procedure for oxidation of **16** described in **S5.5**.

Chromatogram

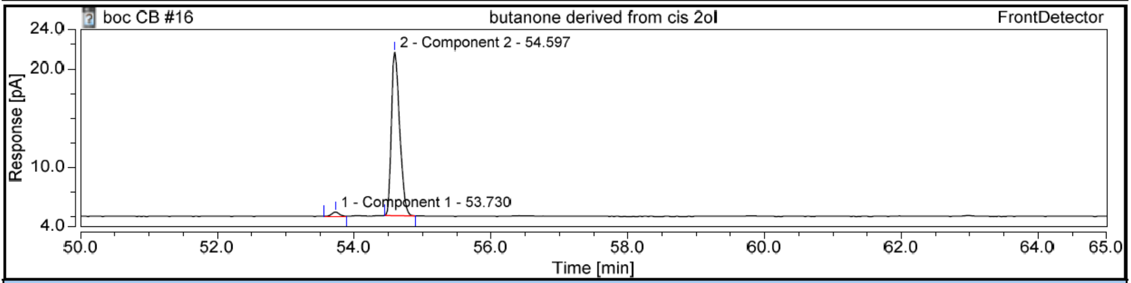

Integration Results

No.	Peak Name	Retention Time min	Area pA*min	Height pA	Relative Area %	Relative Height %	Amount
1	Component 1	53.730	0.055	0.427	2.33	2.51	n.a.
2	Component 2	54.597	2.316	16.588	97.67	97.49	n.a.
**Total:**			**2.371**	**17.014**	**100.00**	**100.00**	

Figure S6.2 – Chiral GC traces of Boc-2-aminocyclobutanones **31**.

S6.3 Determination of the absolute configuration of BCPA derivative (*R*)-**29**.

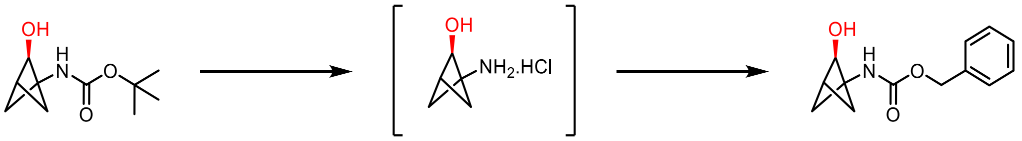

26
absolute configuration
known from X-Ray crystal structure

29

Procedure for synthesis of **29** from **26** described in **S5.5**.

Column: Cyclosil-B [Length: 30 m, Diameter: 0.25 mm, Film thickness: 0.25 µm]

Ramp: t = 0 min: 140 °C; 140 °C hold = 10 min; 1 °C/min between 140–200 °C; 200 °C hold = 40 min; 20 °C/min between 200–220 °C; 220 °C hold = 1 min.

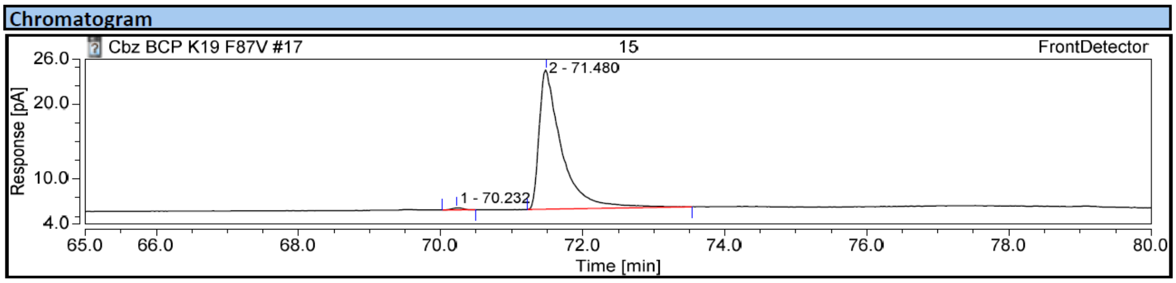

Integration Results

No.	Peak Name	Retention Time min	Area pA*min	Height pA	Relative Area %	Relative Height %	Amount n.a.
1		70.232	0.054	0.290	0.78	1.53	n.a.
2		71.480	6.800	18.634	99.22	98.47	n.a.
**Total:**			**6.854**	**18.924**	**100.00**	**100.00**	

Figure S6.3a – GC trace of Cbz-**29** derived from Boc-**26**.

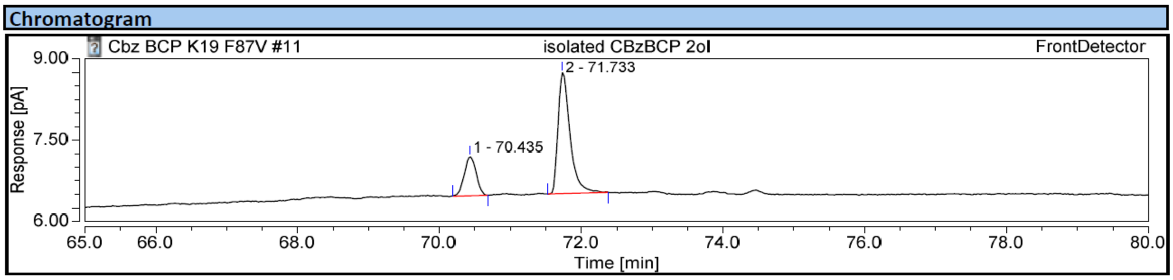

Integration Results

No.	Peak Name	Retention Time min	Area pA*min	Height pA	Relative Area %	Relative Height %	Amount n.a.
1		70.435	0.142	0.722	24.41	24.46	n.a.
2		71.733	0.438	2.230	75.59	75.54	n.a.
**Total:**			**0.580**	**2.952**	**100.00**	**100.00**	

Figure S6.3b – GC trace of Cbz-**29** from substrate **13d** and K19/FA/IA (51% *ee* in favor of (2*R*)-).

S6.4 Determination of absolute configuration of **22** by Mosher ester analysis.

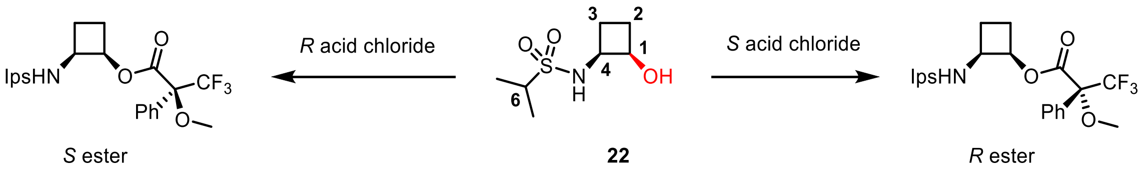

22
isolated from reaction
with KU3/AP/SW

Mosher esters prepared according to Hoye protocol,[10] in an NMR tube using 5.0 mg **22**.

Proton	δ (*S*)-ester (ppm)	δ (*R*)-ester (ppm)	$\Delta\delta^{SR}$ (ppm)	×600 (MHz)
**2**	2.01	1.89	+0.12	+72
**3**	2.37	2.34	+0.03	+18
**4**	4.24	4.32	–0.08	–48
**6**	2.94	3.07	–0.13	–78

By comparison of the chemical shifts of diagnostic protons of the Mosher derived (*S*)-ester and (*R*)-ester, the absolute configuration of the C(OH) stereocentre of **22** was determined to be (*R*).

S6.5 Chiral GC traces of samples used to obtain crystal structures.

(1) Chiral GC trace of **14** used to obtain crystal data (*er* >99:1; Table S8.1):
Column: Cyclosil-B [Length: 30 m, Diameter: 0.25 mm, Film thickness: 0.25 µm]
Ramp: t = 0 min: 90 °C; 0.5 °C/min between 90–140 °C; 140 °C hold = 10 min; 10 °C/min between 140–200 °C; 200 °C hold = 1 min.

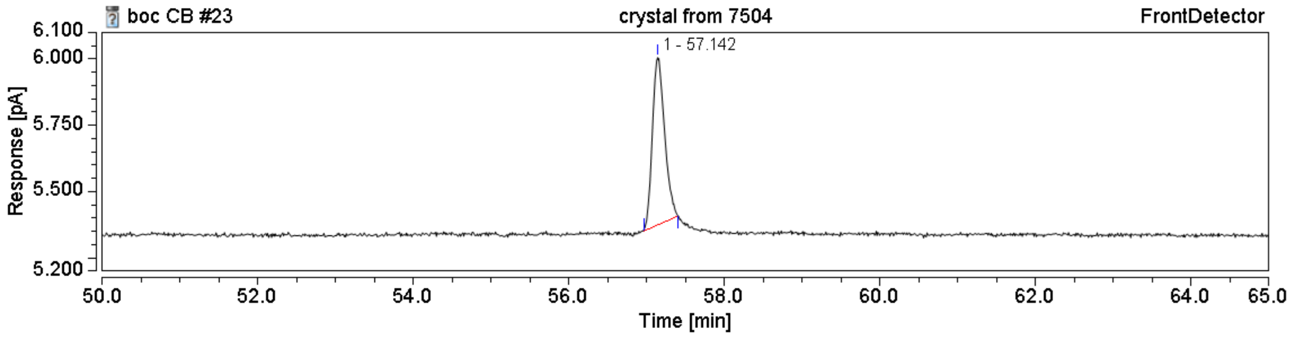

(2a) Chiral GC trace of exact crystal of **26** used to obtain crystal data (*er* ~70:30, 2*R*-major; Table S8.6):
Column: Cyclosil-B [Length: 30 m, Diameter: 0.25 mm, Film thickness: 0.25 µm]
Ramp: t = 0 min: 60 °C; 1 °C/min between 60–180 °C; 180 °C hold = 10 min

Peaks at 81–82.5 min are artefacts of extraction from the grease used to mount the crystal.

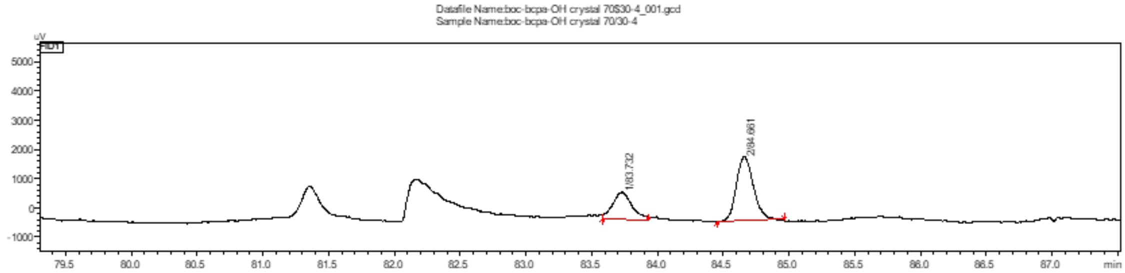

Peak#	Ret. Time	Area	Height	Area%	Height%
1	83.732	9556	929	32.813	29.759
2	84.661	19567	2193	67.187	70.241
Total		29124	3122	100.000	100.000

(2b) Chiral GC trace from bulk sample of **26** (*er* ~70:30) from which the above crystal was picked.

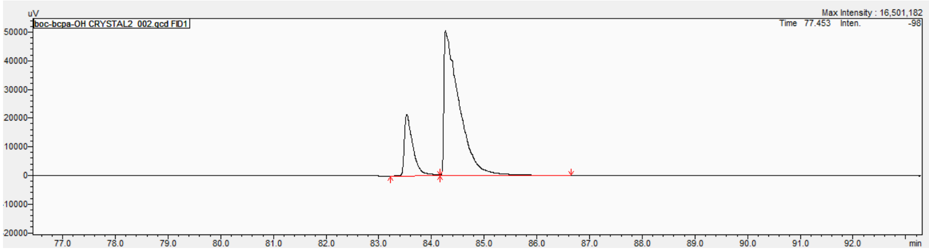

Peak#	Ret.	Area	Height	Area%	Height%
1	83.53	243786	21408	19.371	29.774
2	84.26	1014715	50494	80.629	70.226
Total		1258501	71903	100.000	100.000

(2c) A second crystal of **26** was picked from the same crystallization; this was shown to be the major (*R*)-enantiomer (Table S8.7) although insufficient material was extracted from the mounting grease for chiral GC.

S7 NMR spectra

12a ^{1}H NMR ($CDCl_3$, 400 MHz)

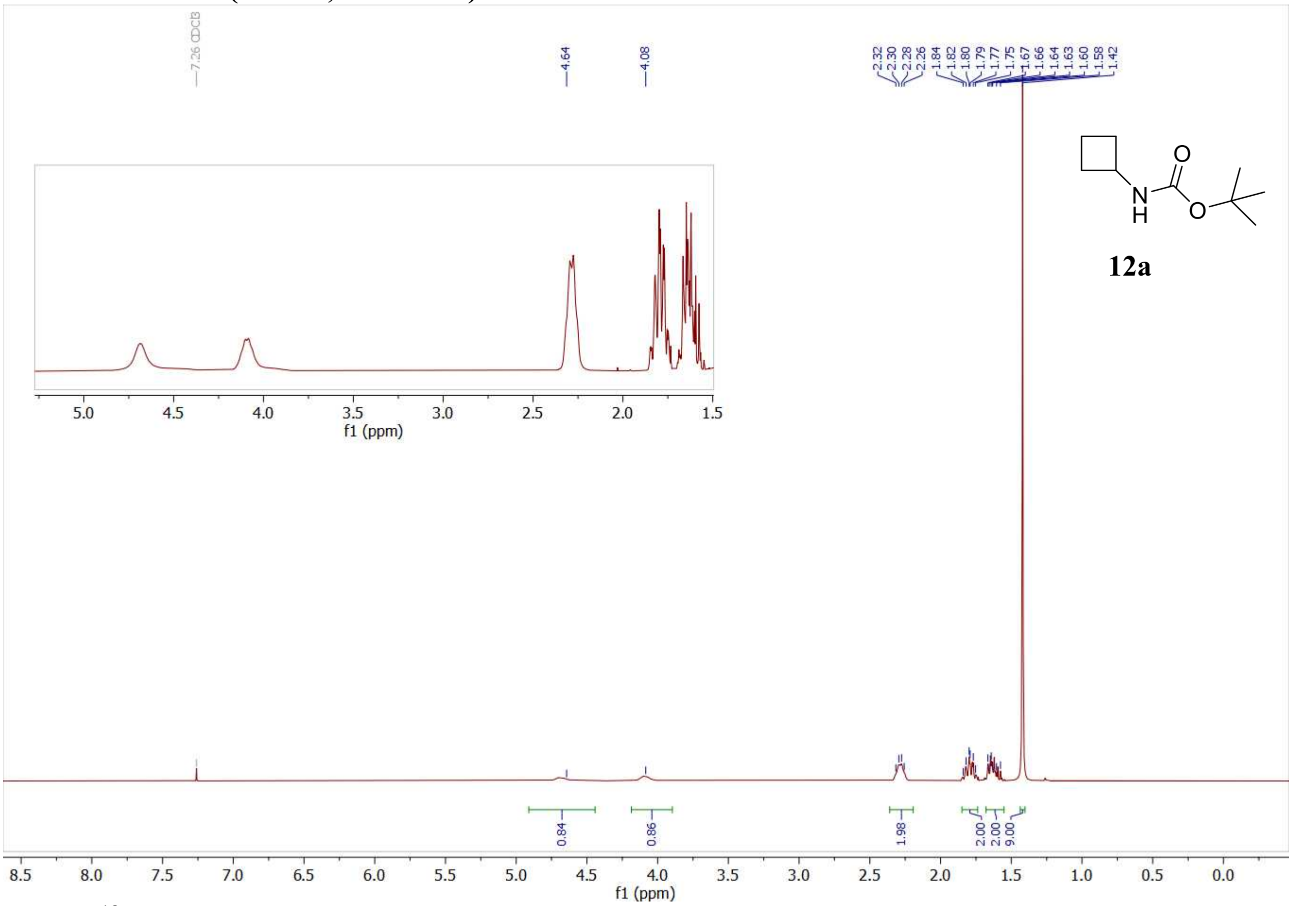

12a ^{13}C NMR ($CDCl_3$, 101 MHz)

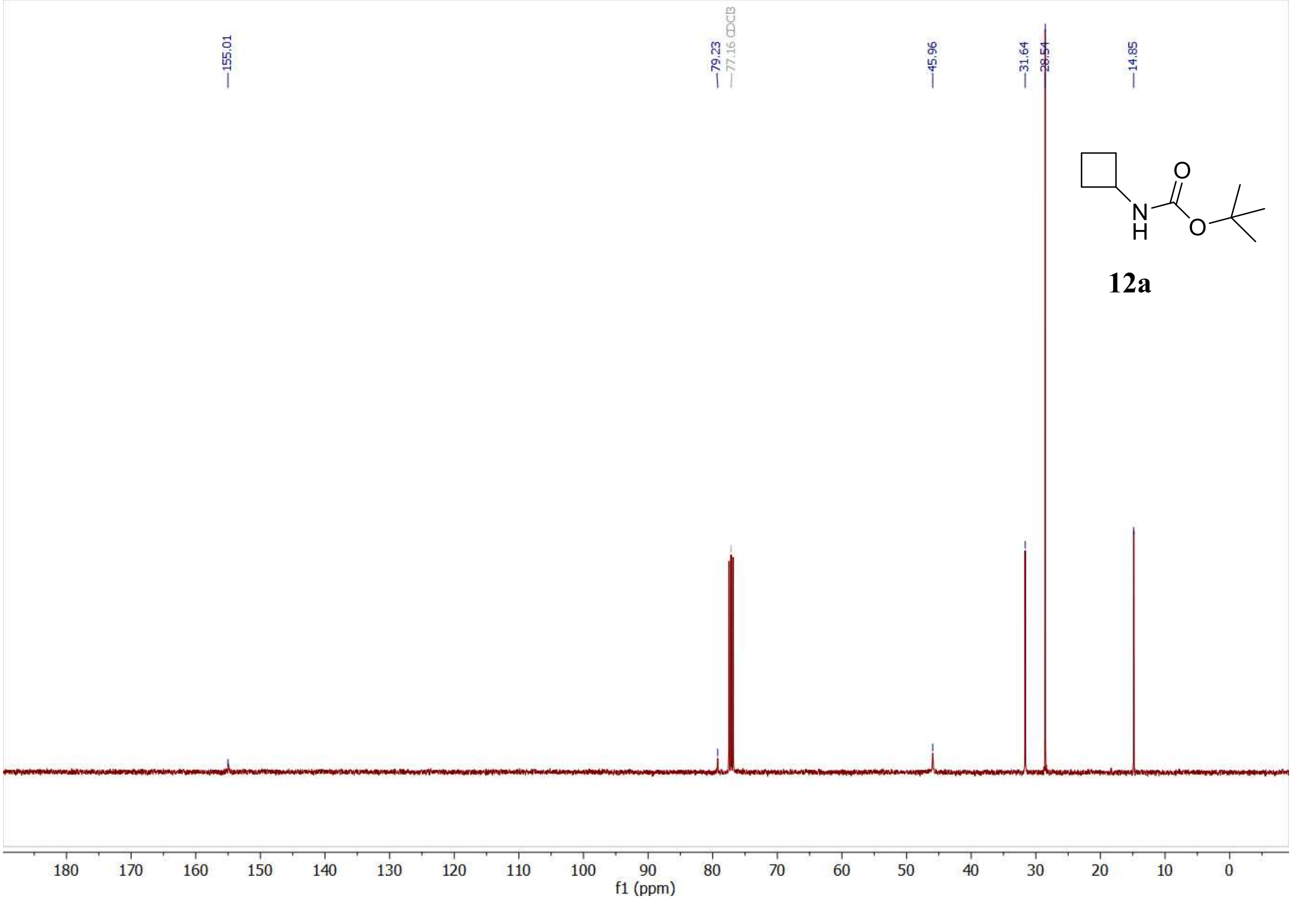

12b **^{1}H NMR ($CDCl_3$, 400 MHz)**

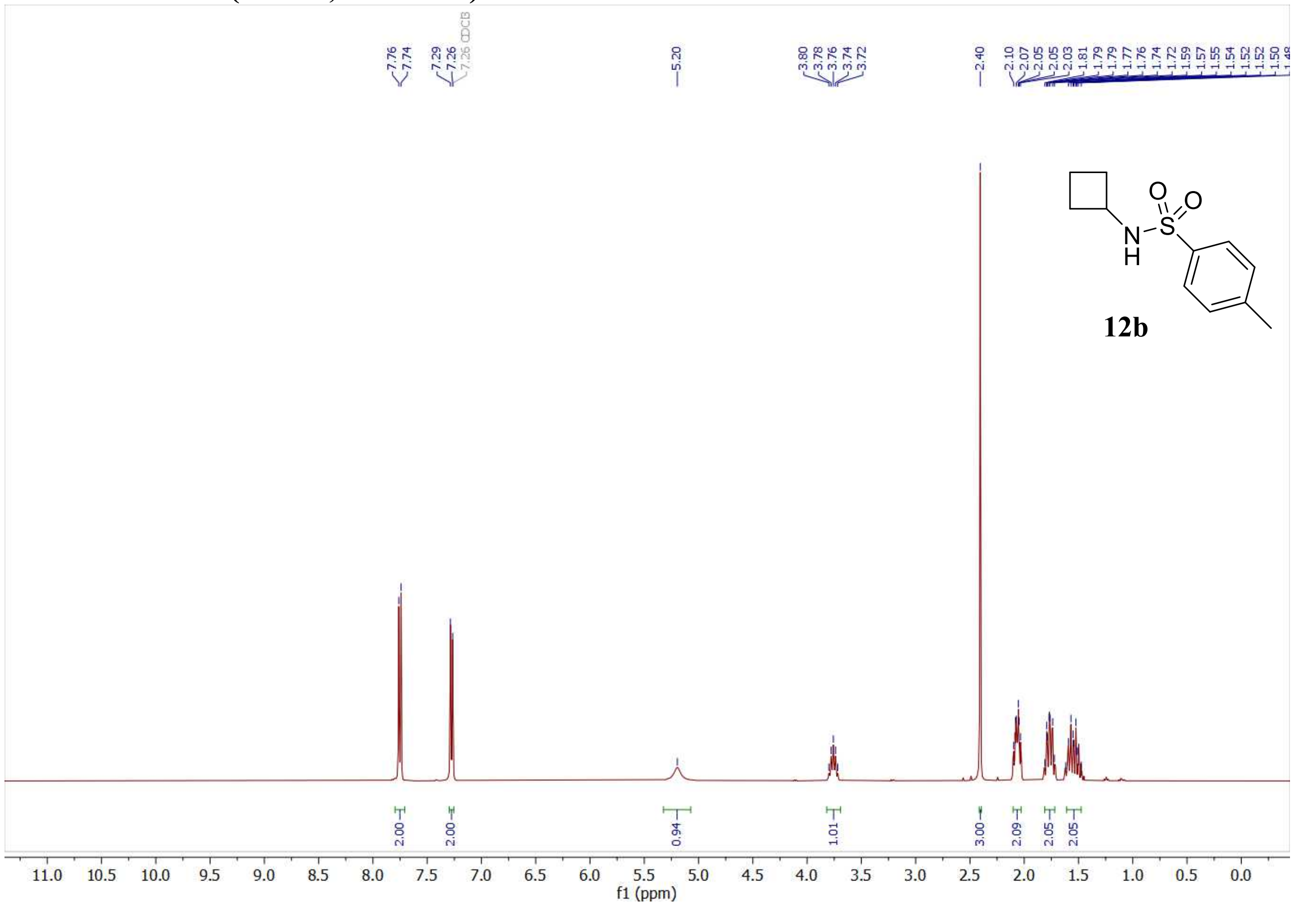

12b **^{13}C NMR ($CDCl_3$, 101 MHz)**

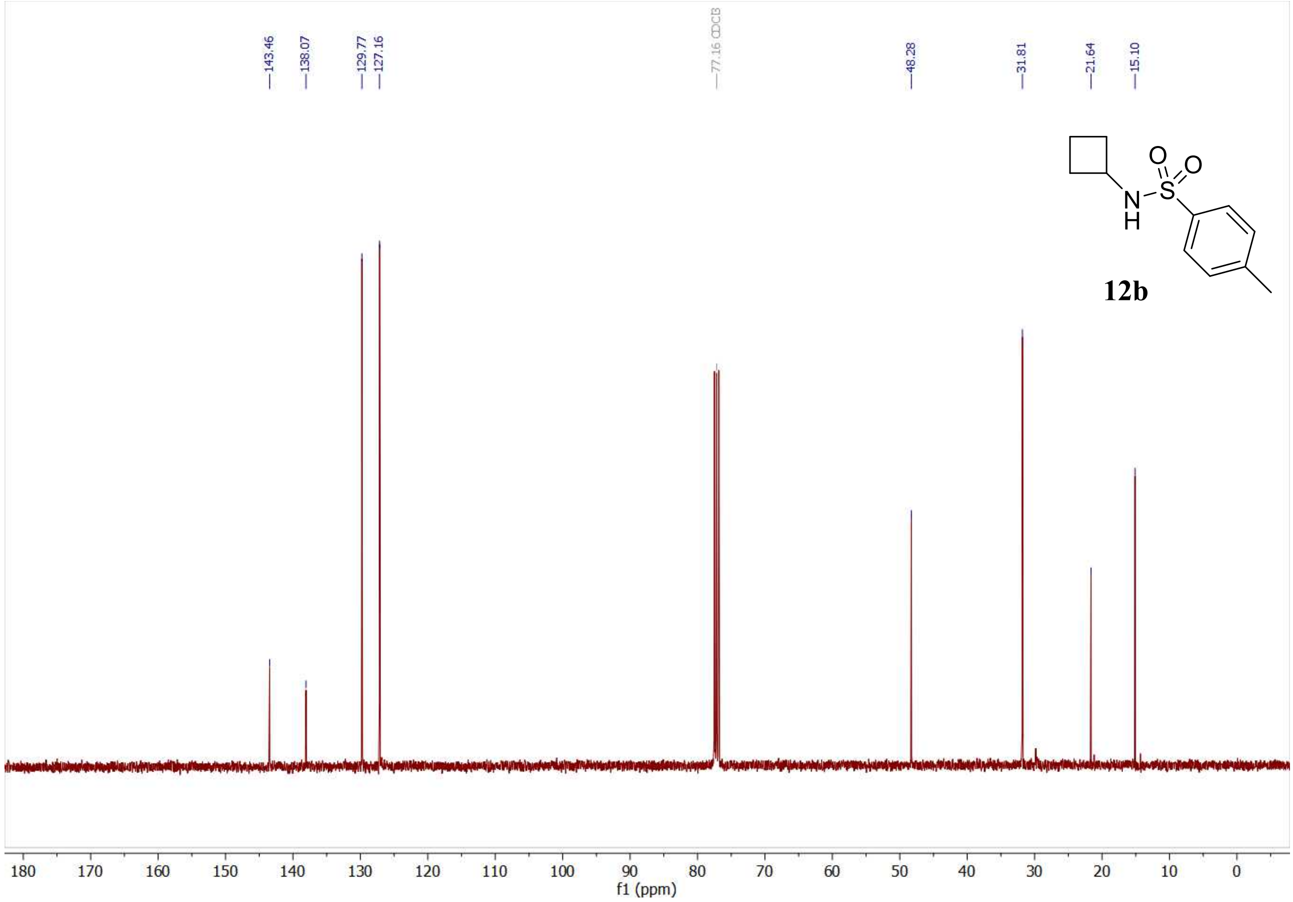

12c ^{1}H NMR ($CDCl_3$, 400 MHz)

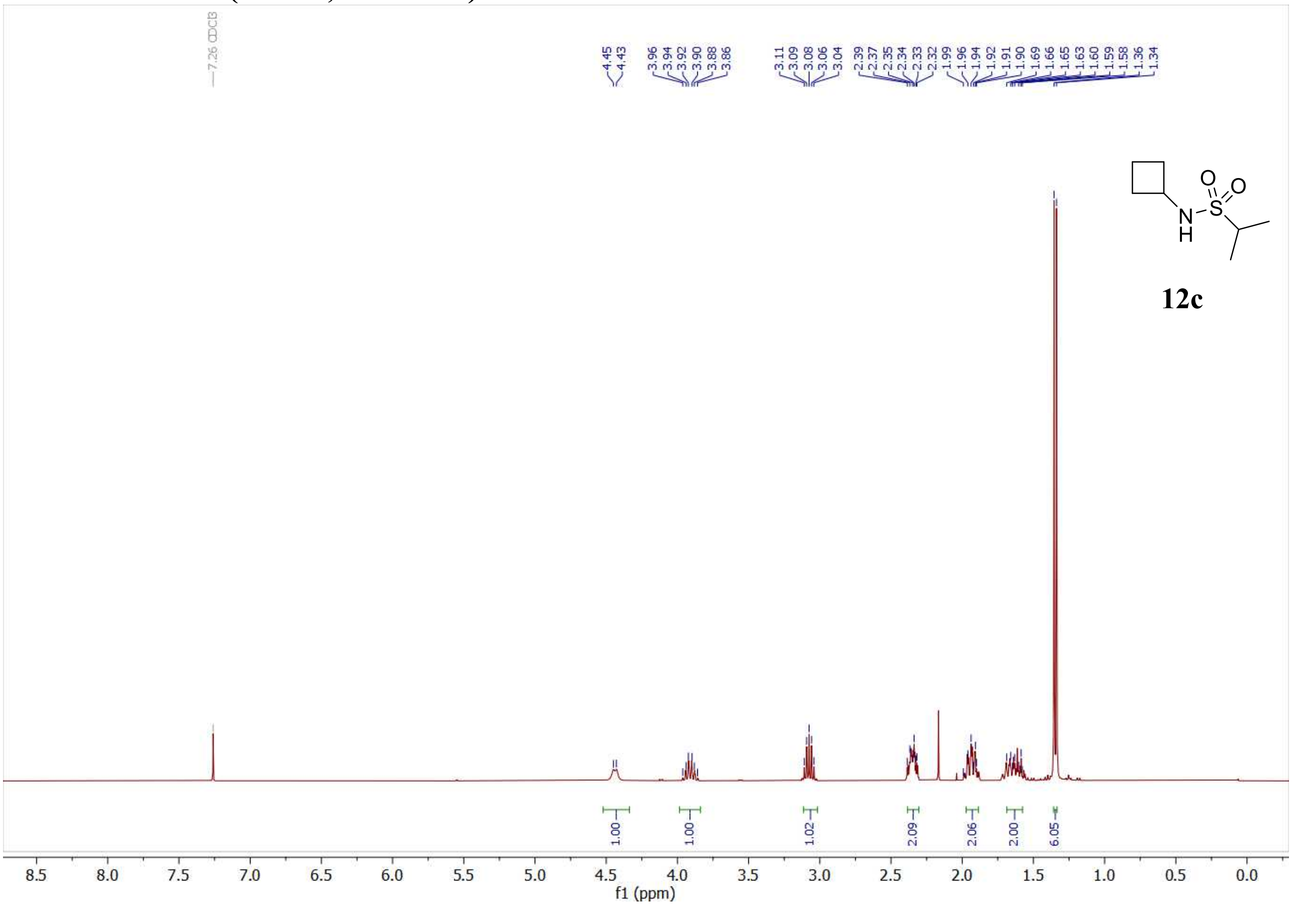

12c ^{13}C NMR ($CDCl_3$, 101 MHz)

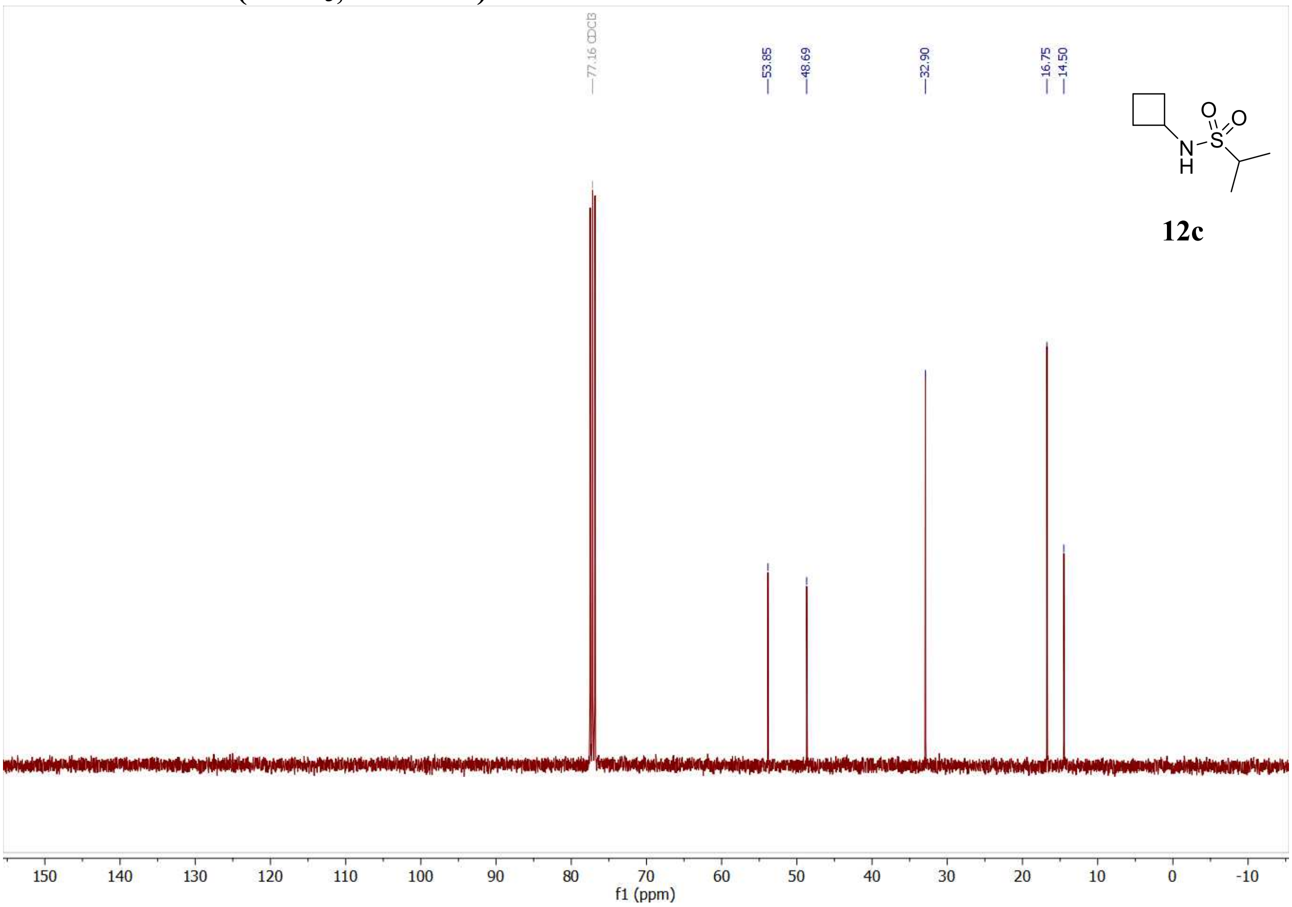

13a ^{1}H NMR ($CDCl_3$, 400 MHz)

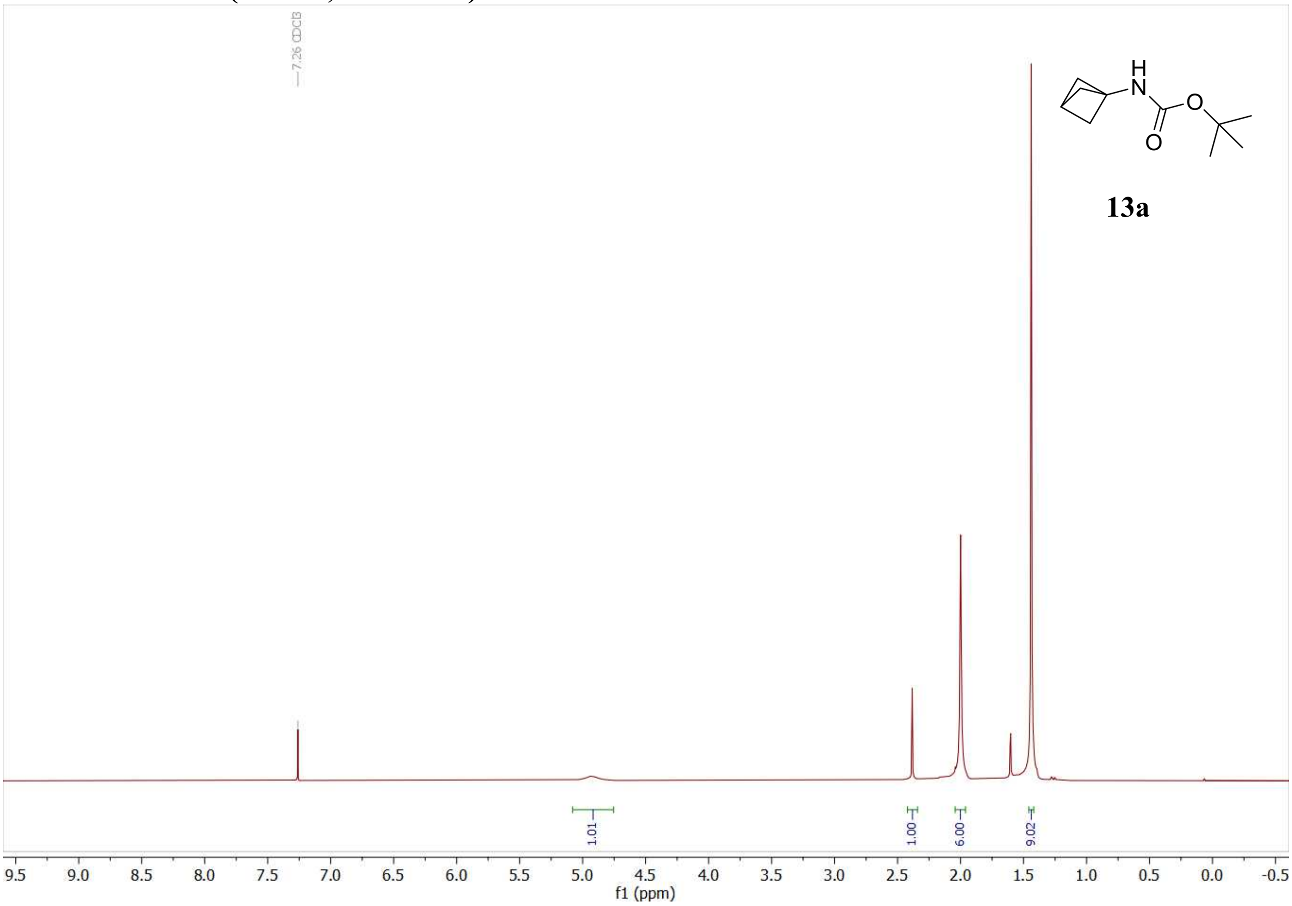

13a ^{13}C NMR ($CDCl_3$, 126 MHz)

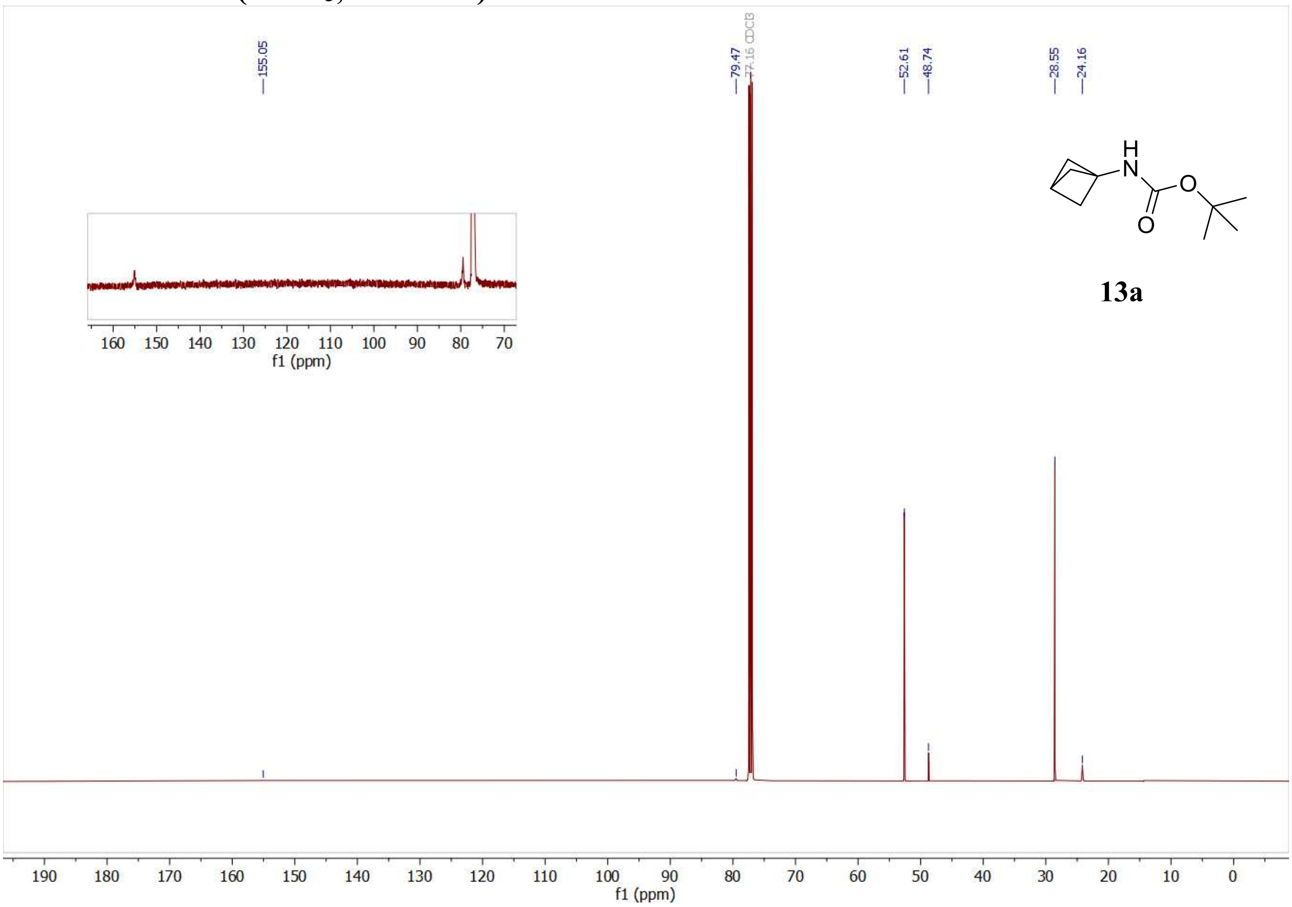

13d ^{1}H NMR ($CDCl_3$, 400 MHz)

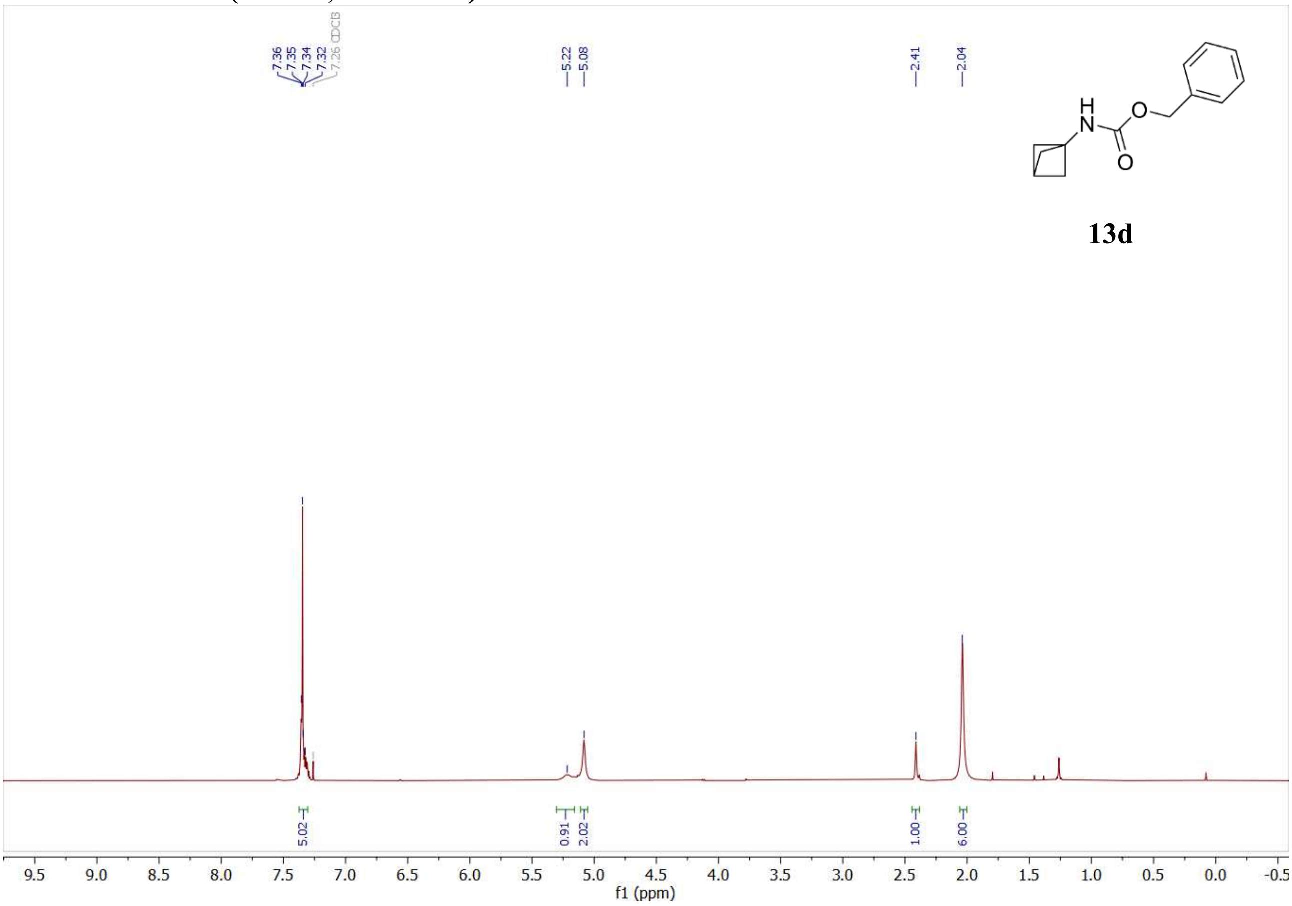

13d ^{13}C NMR ($CDCl_3$, 101 MHz)

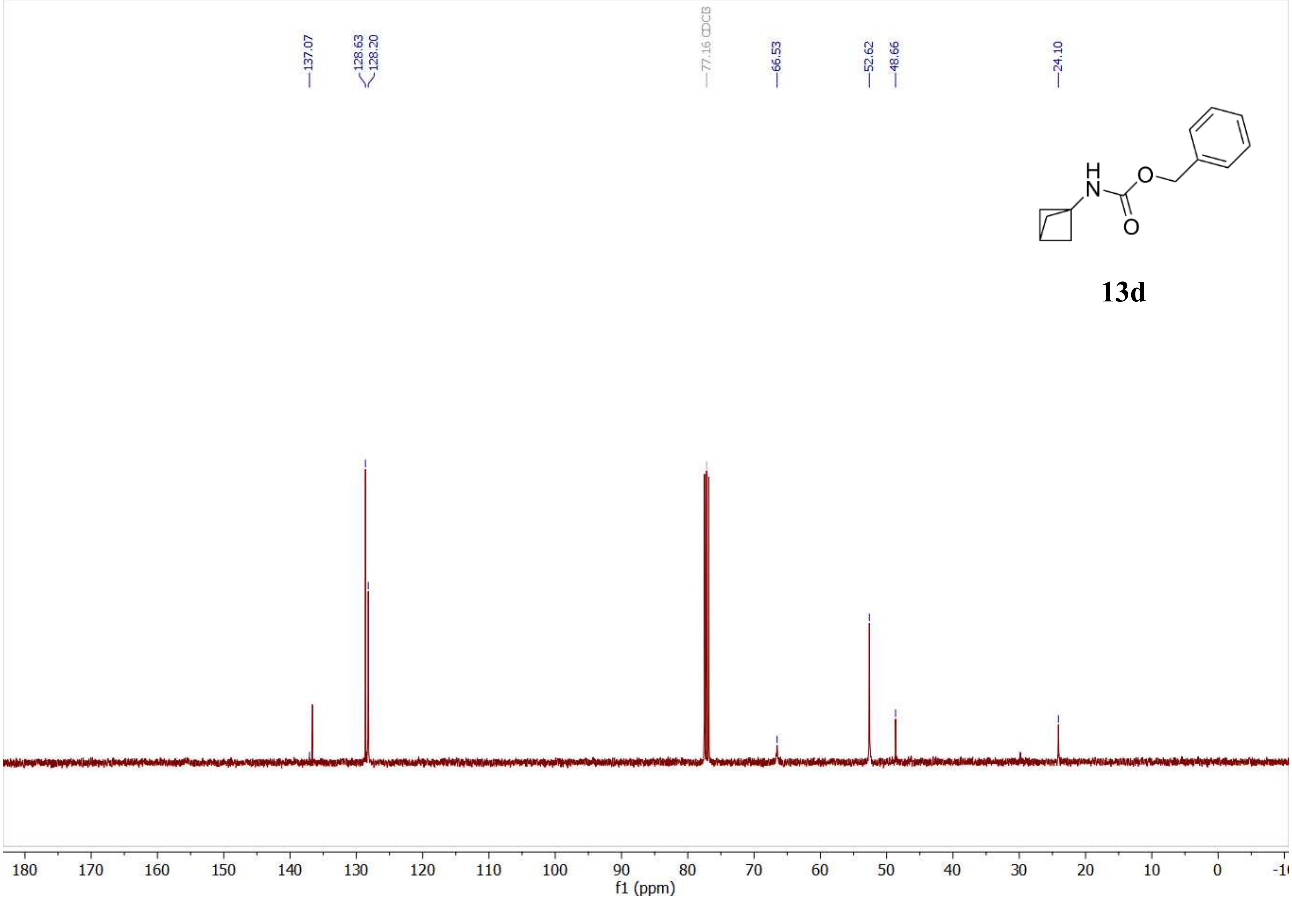

14 ^{1}H NMR ($CDCl_3$, 400 MHz)

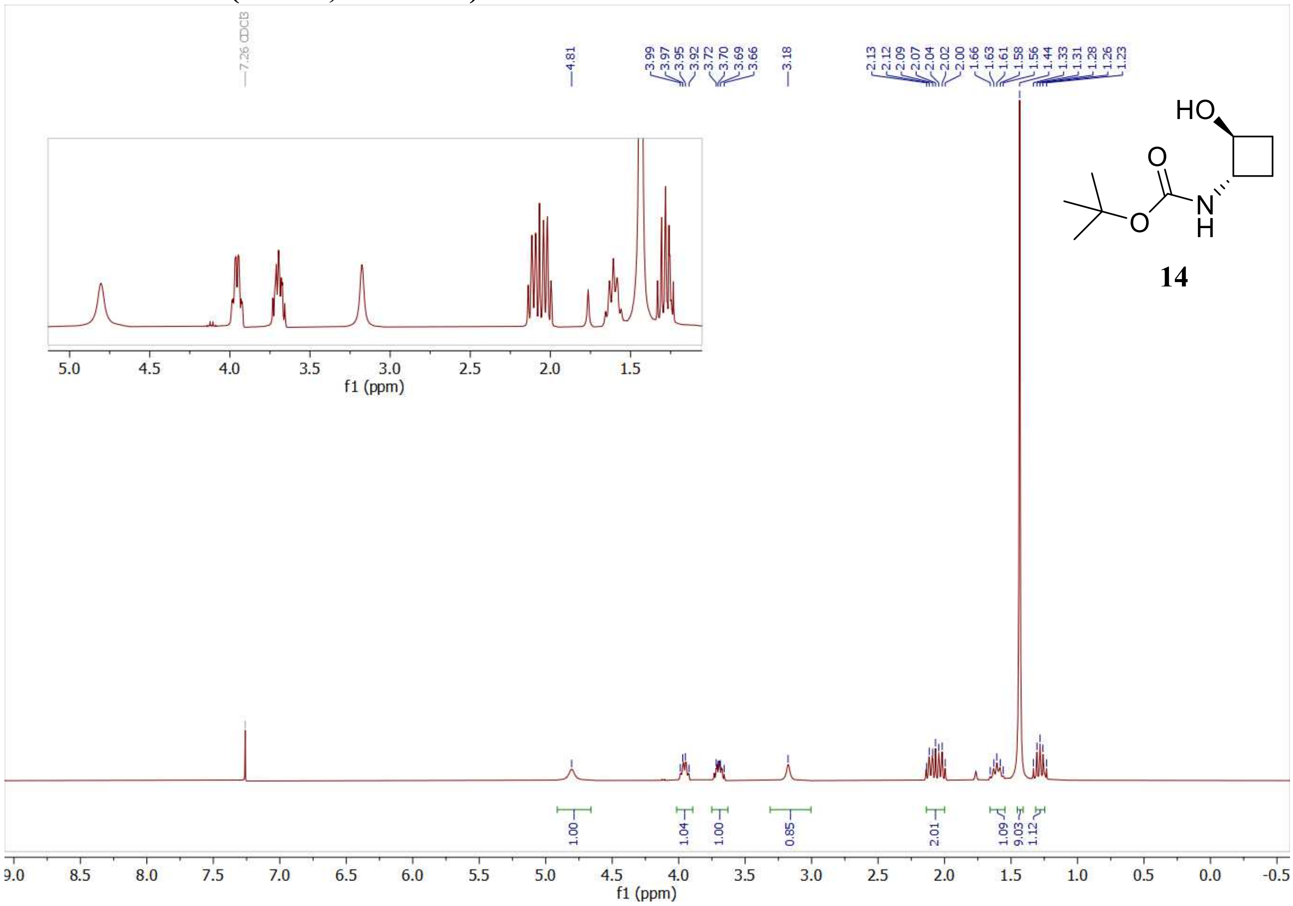

14 ^{13}C NMR ($CDCl_3$, 101 MHz)

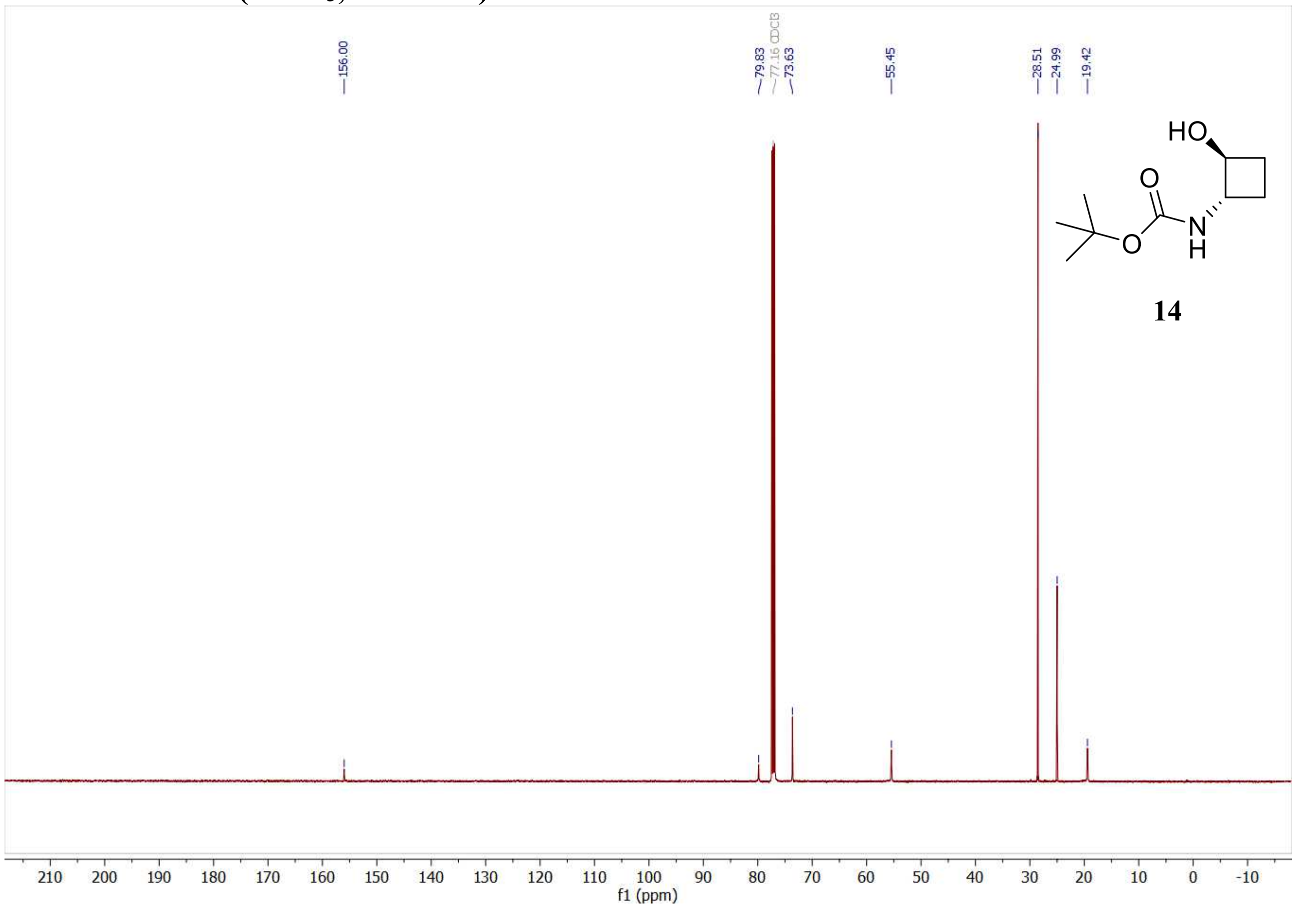

15 & 17 ^{1}H NMR ($CDCl_3$, 500 MHz)

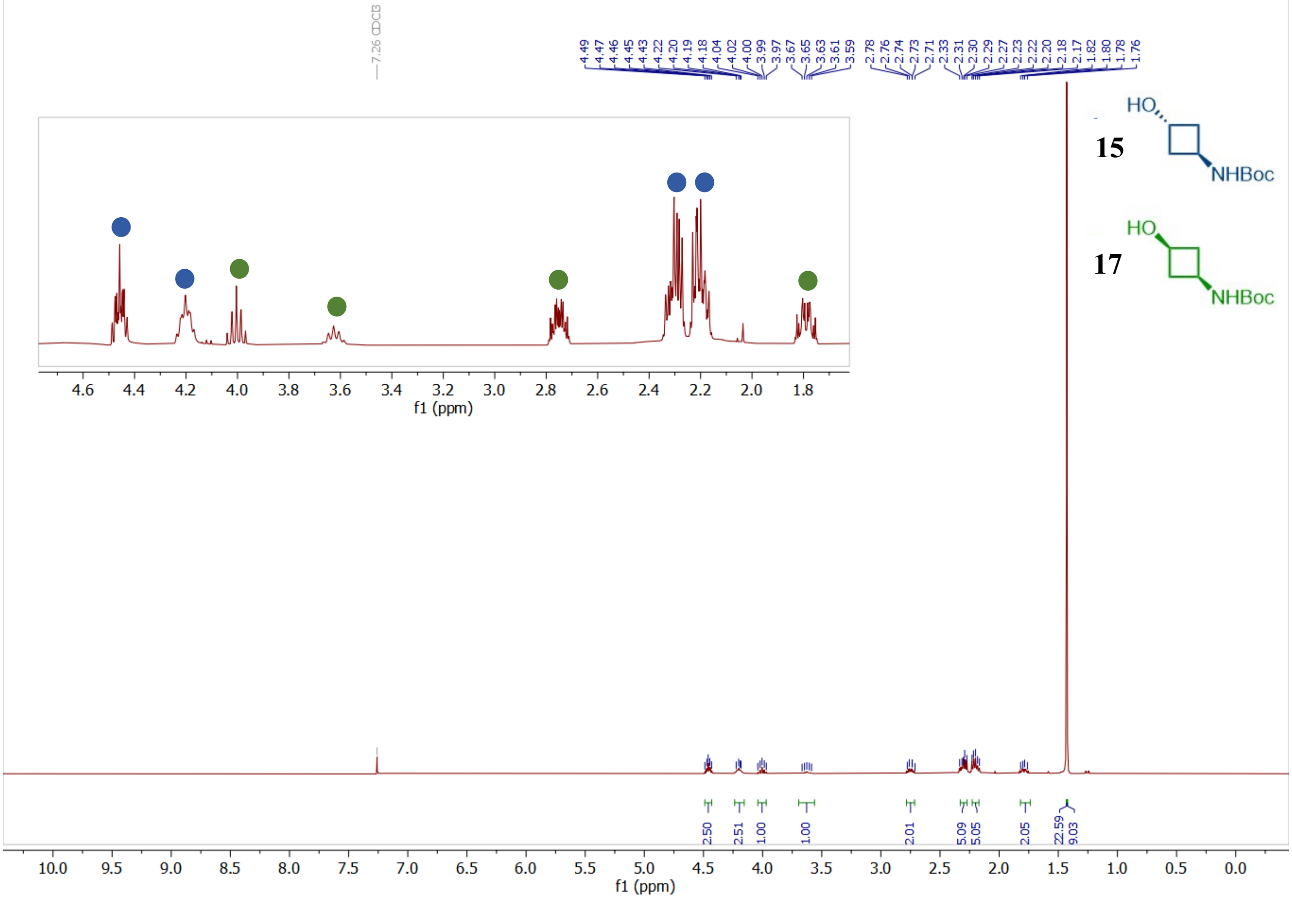

15 & 17 ^{13}C NMR ($CDCl_3$, 126 MHz)

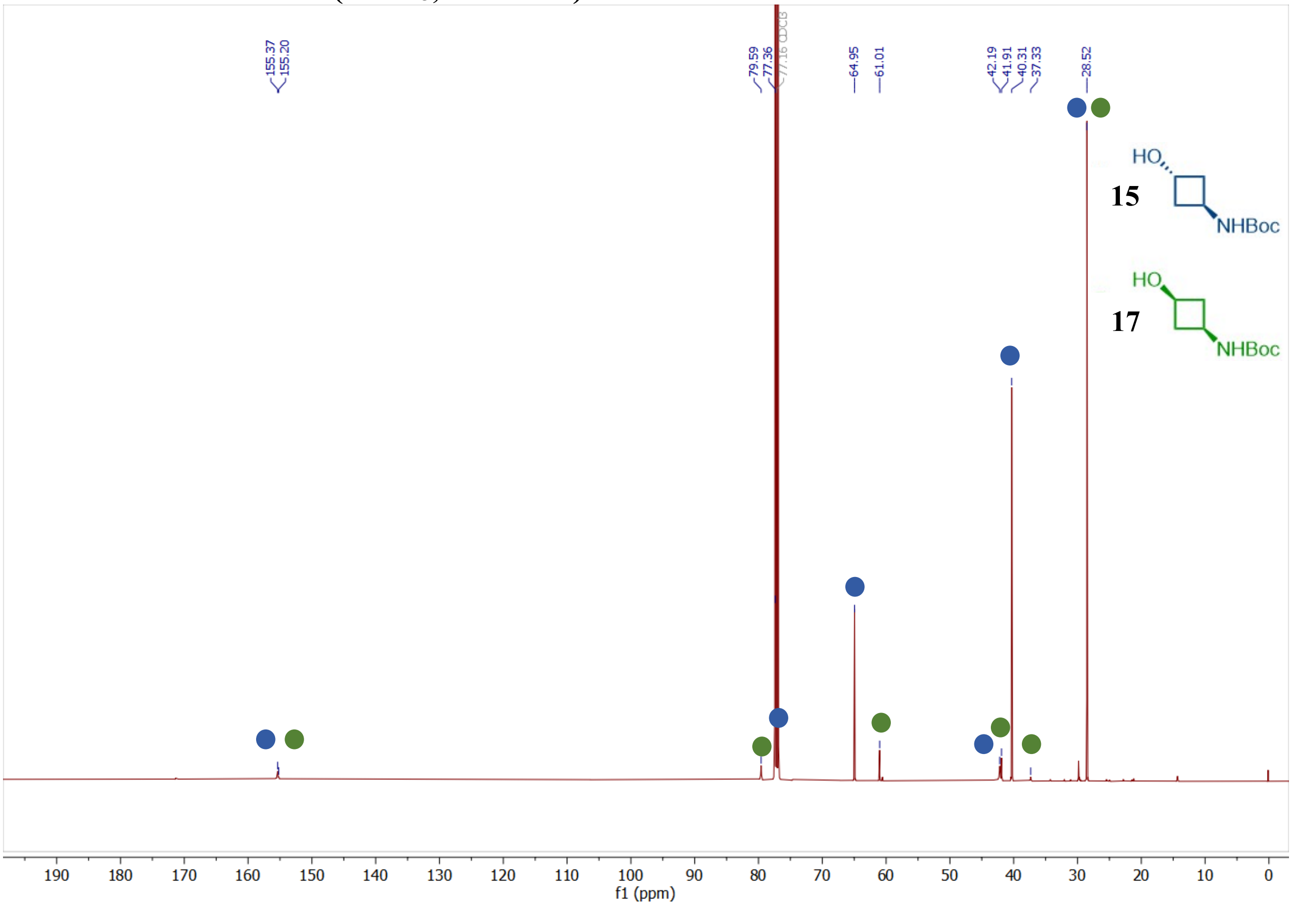

16 ^{1}H NMR ($CDCl_3$, 500 MHz)

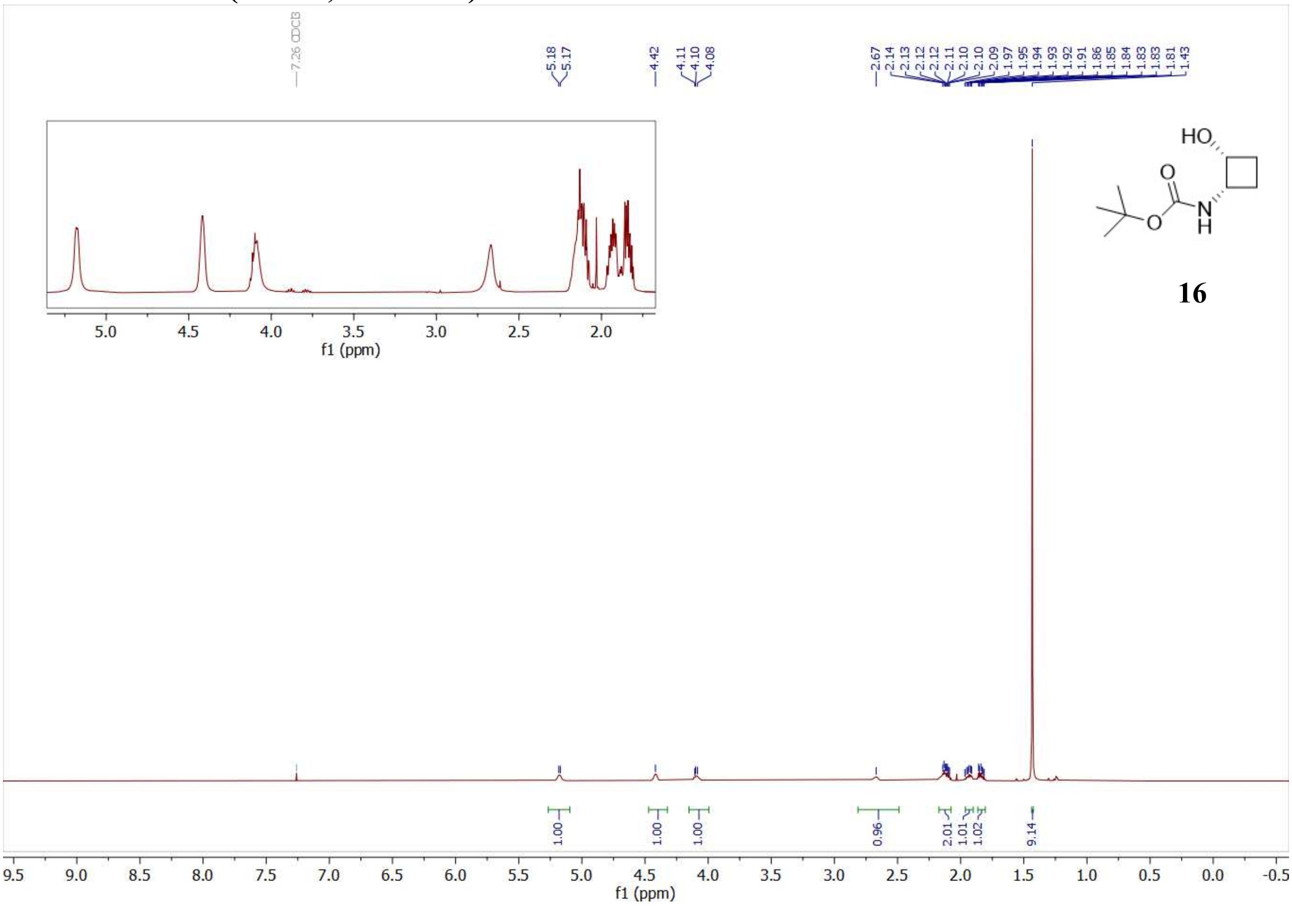

16 ^{13}C NMR ($CDCl_3$, 126 MHz)

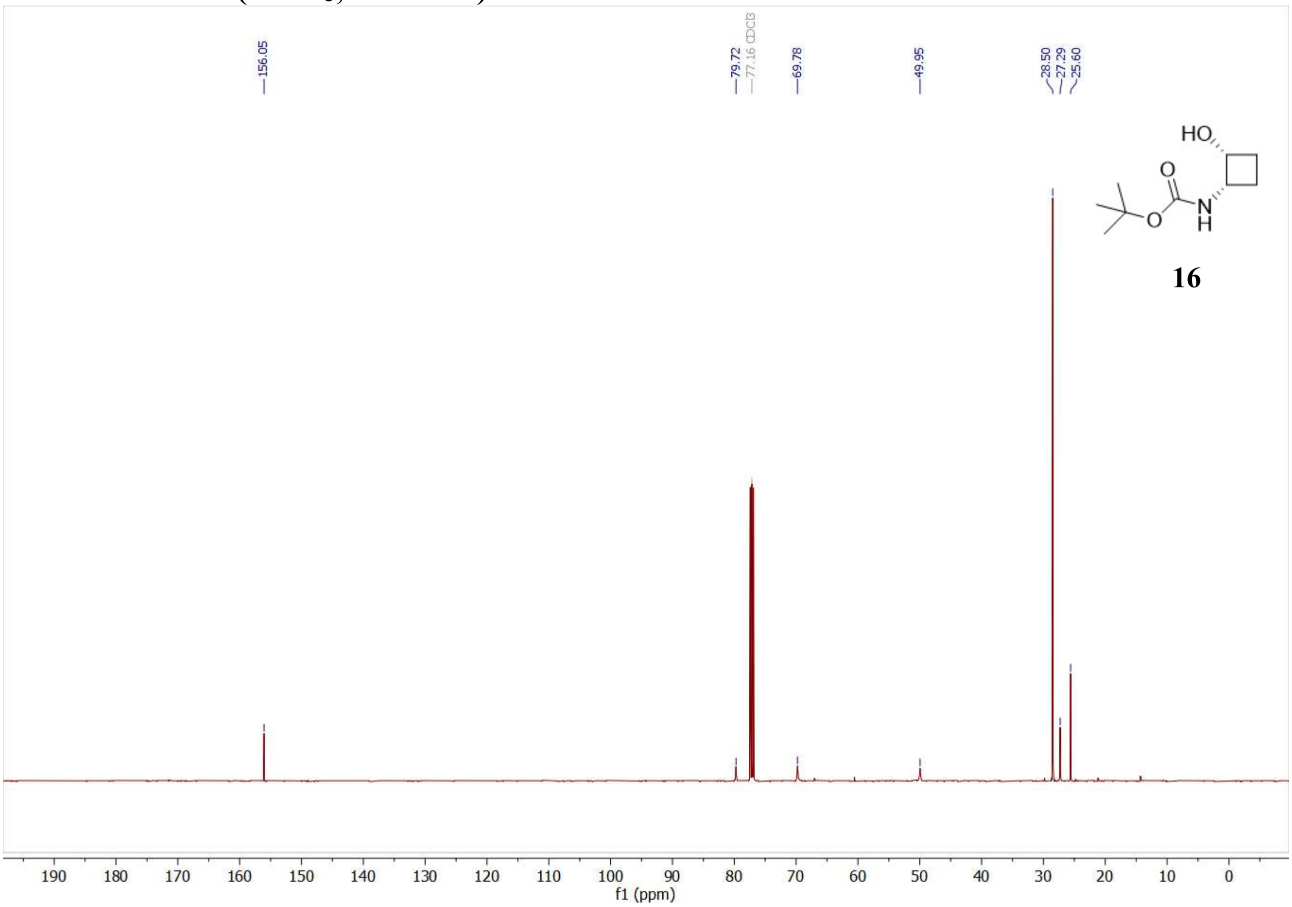

18 ^{1}H NMR (DMSO-d_6, 400 MHz)

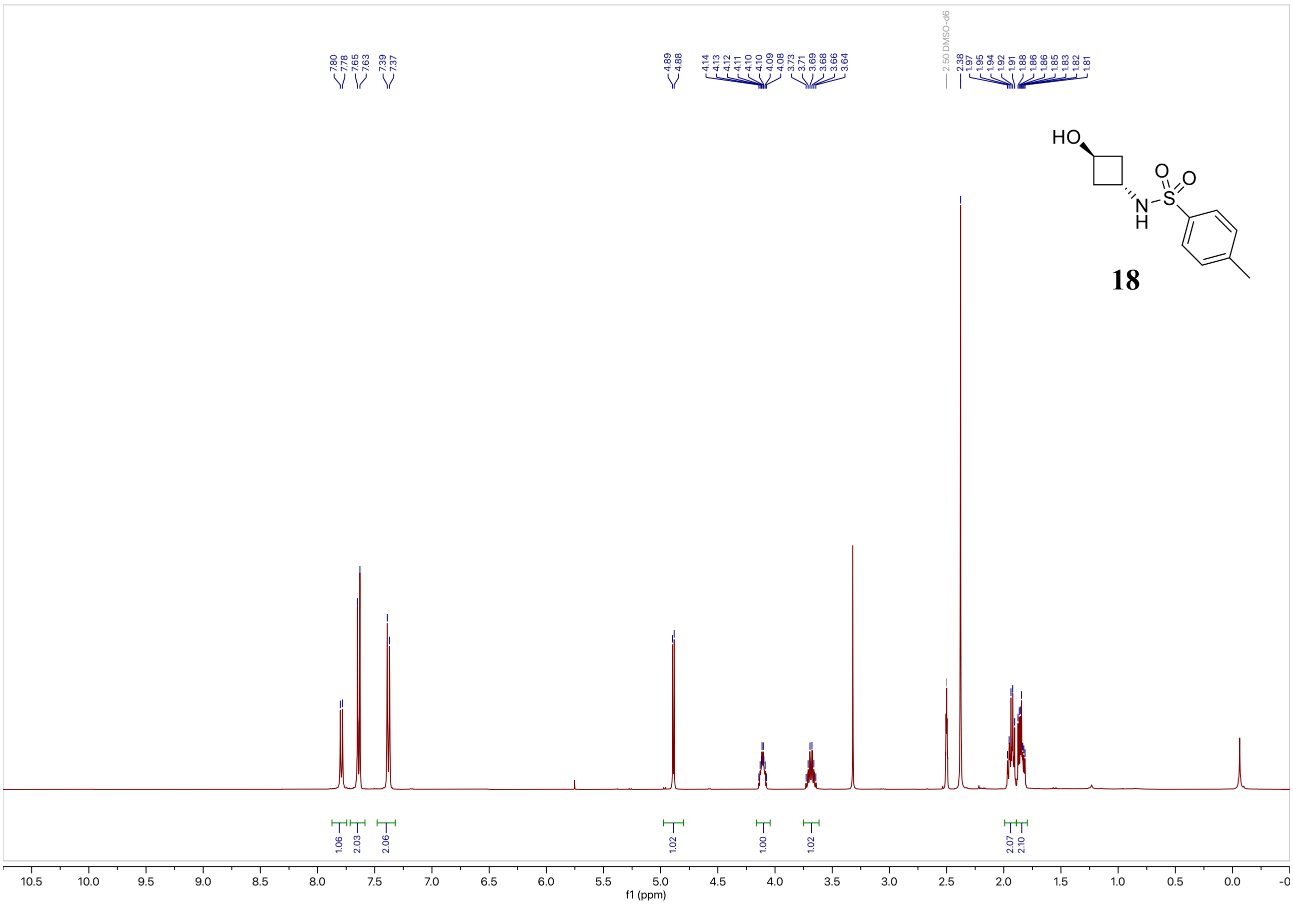

18 ^{13}C NMR (DMSO-d_6, 101 MHz)

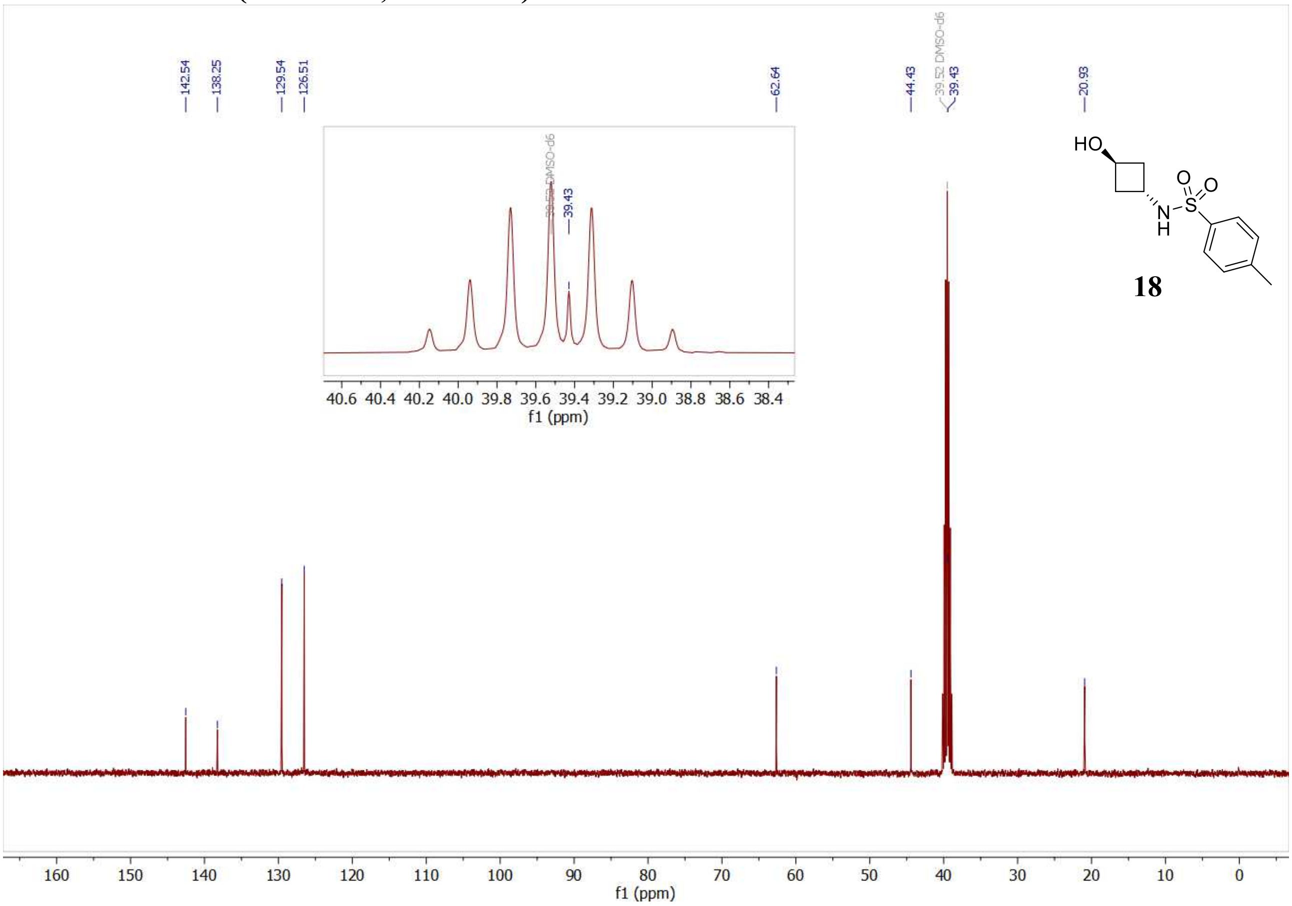

19 **^{1}H NMR ($CDCl_3$, 400 MHz)**

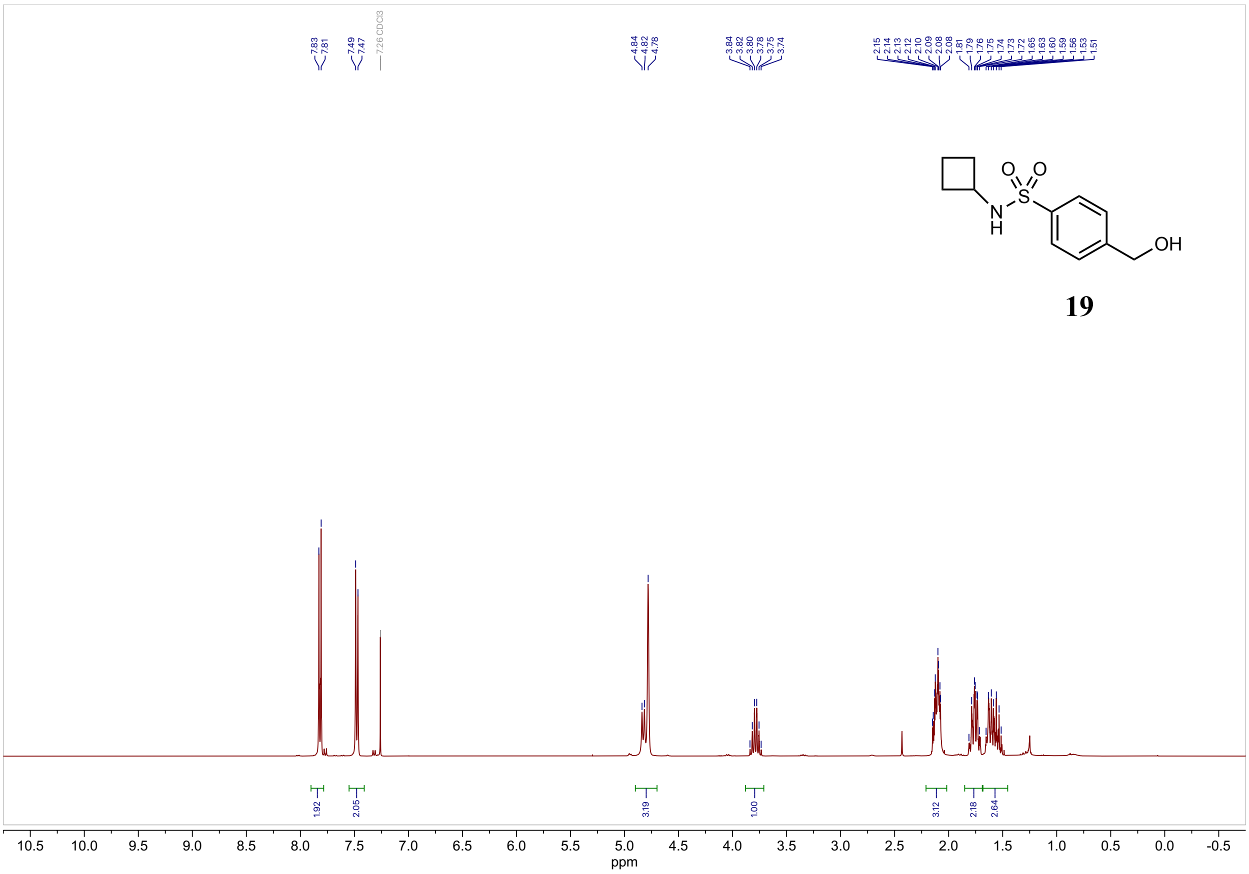

21 ^{1}H NMR ($CDCl_3$, 500 MHz)

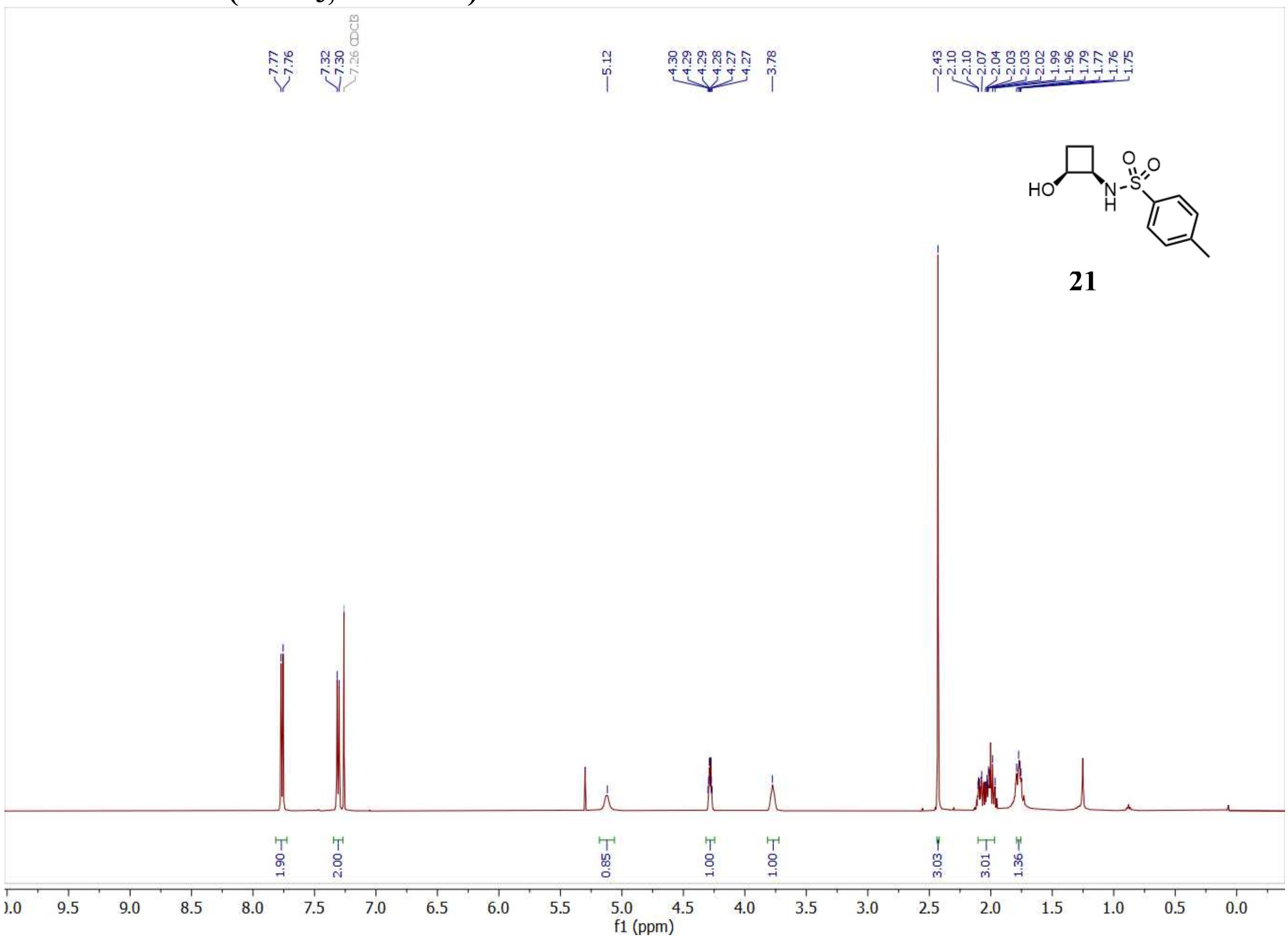

21

21 ^{13}C NMR ($CDCl_3$, 126 MHz)

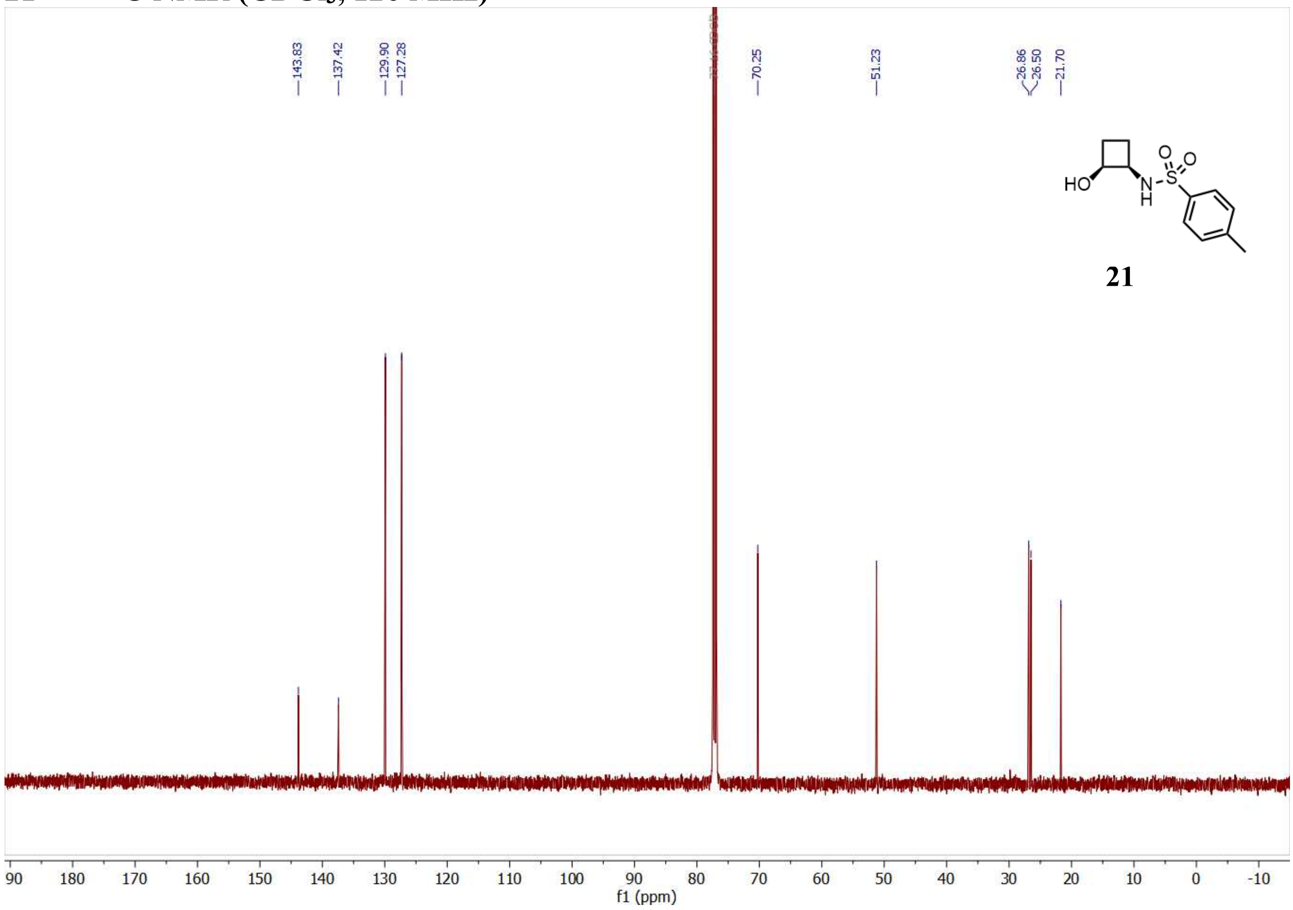

21

22 **^{1}H NMR ($CDCl_3$, 400 MHz)**

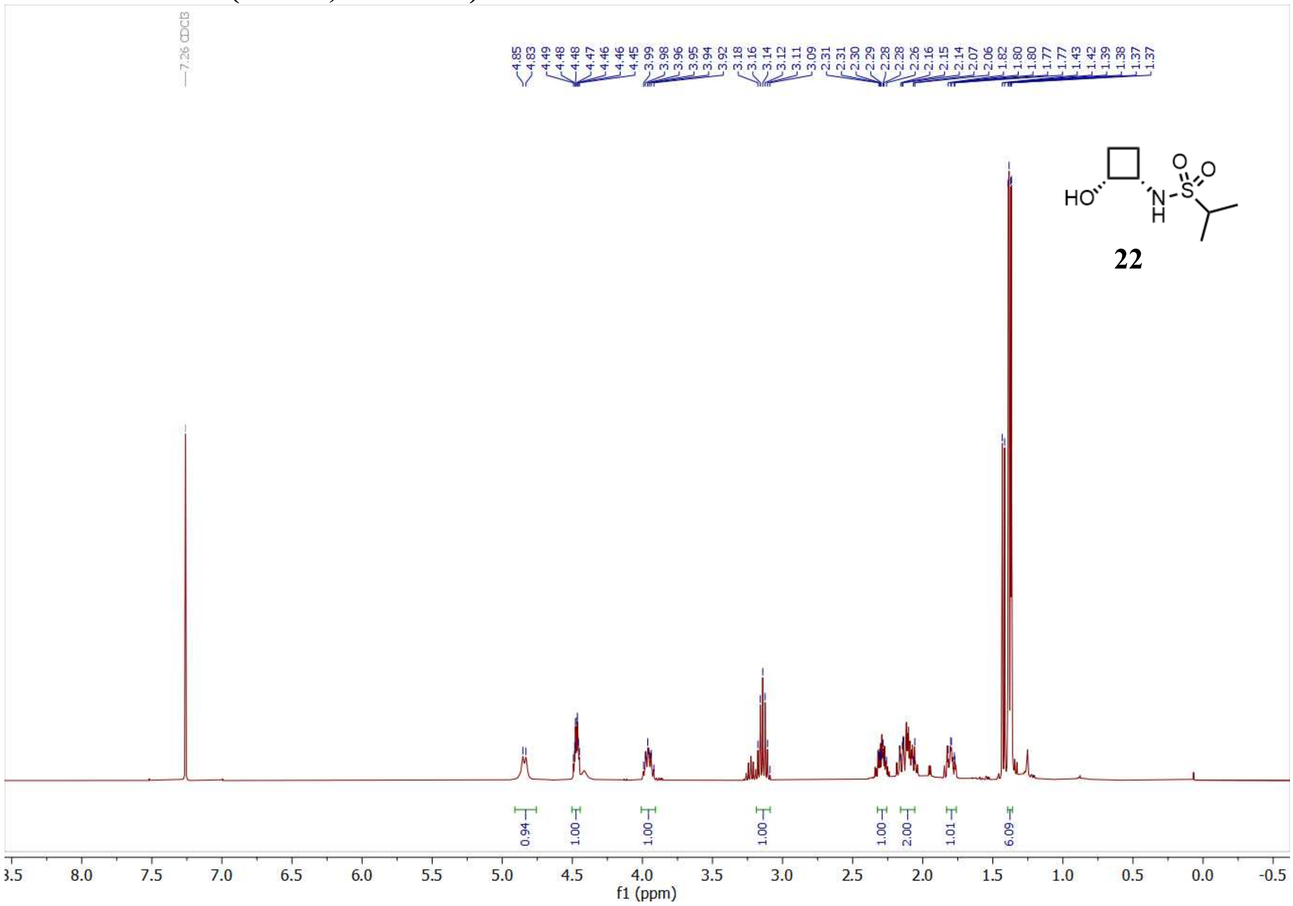

22 **^{13}C NMR ($CDCl_3$, 101 MHz)**

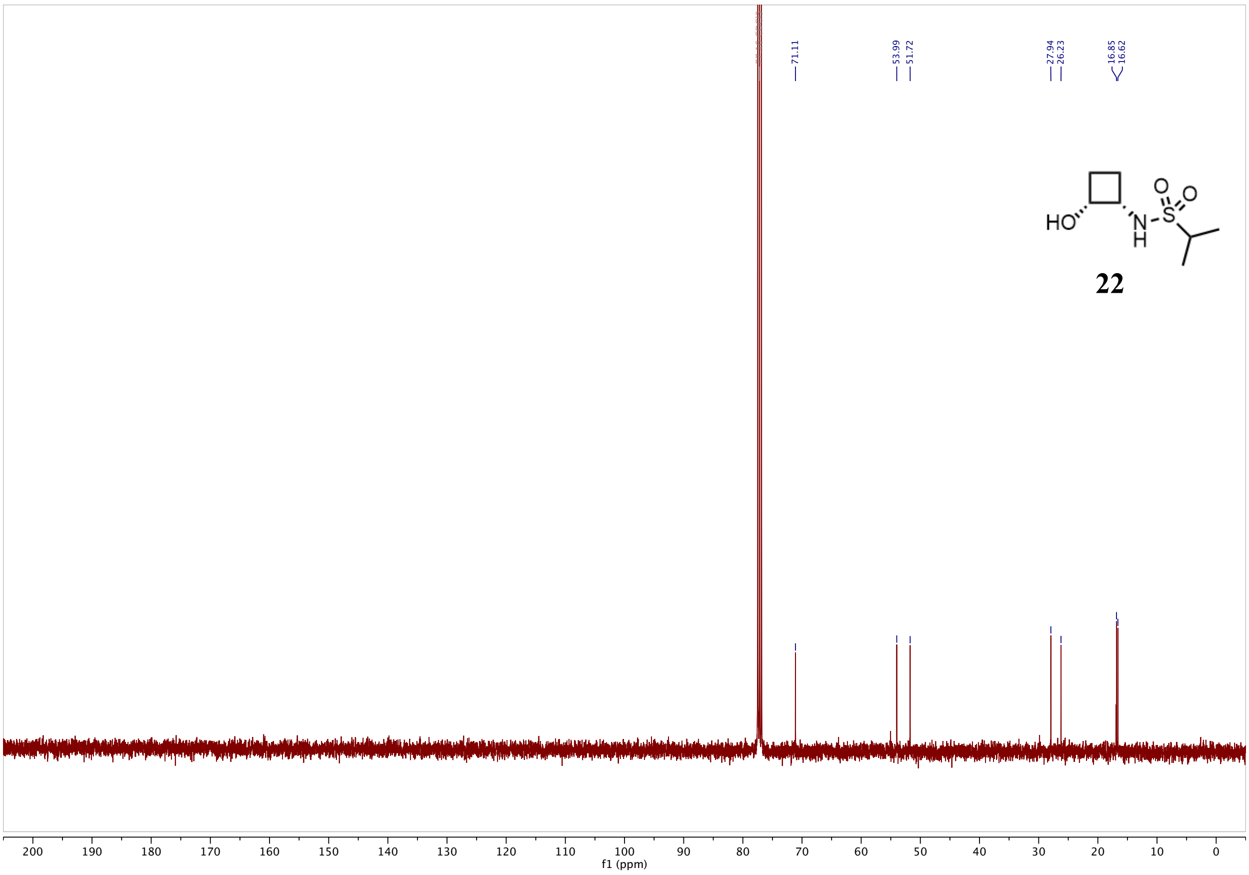

23 ^{1}H NMR (CDCl$_3$, 400 MHz)

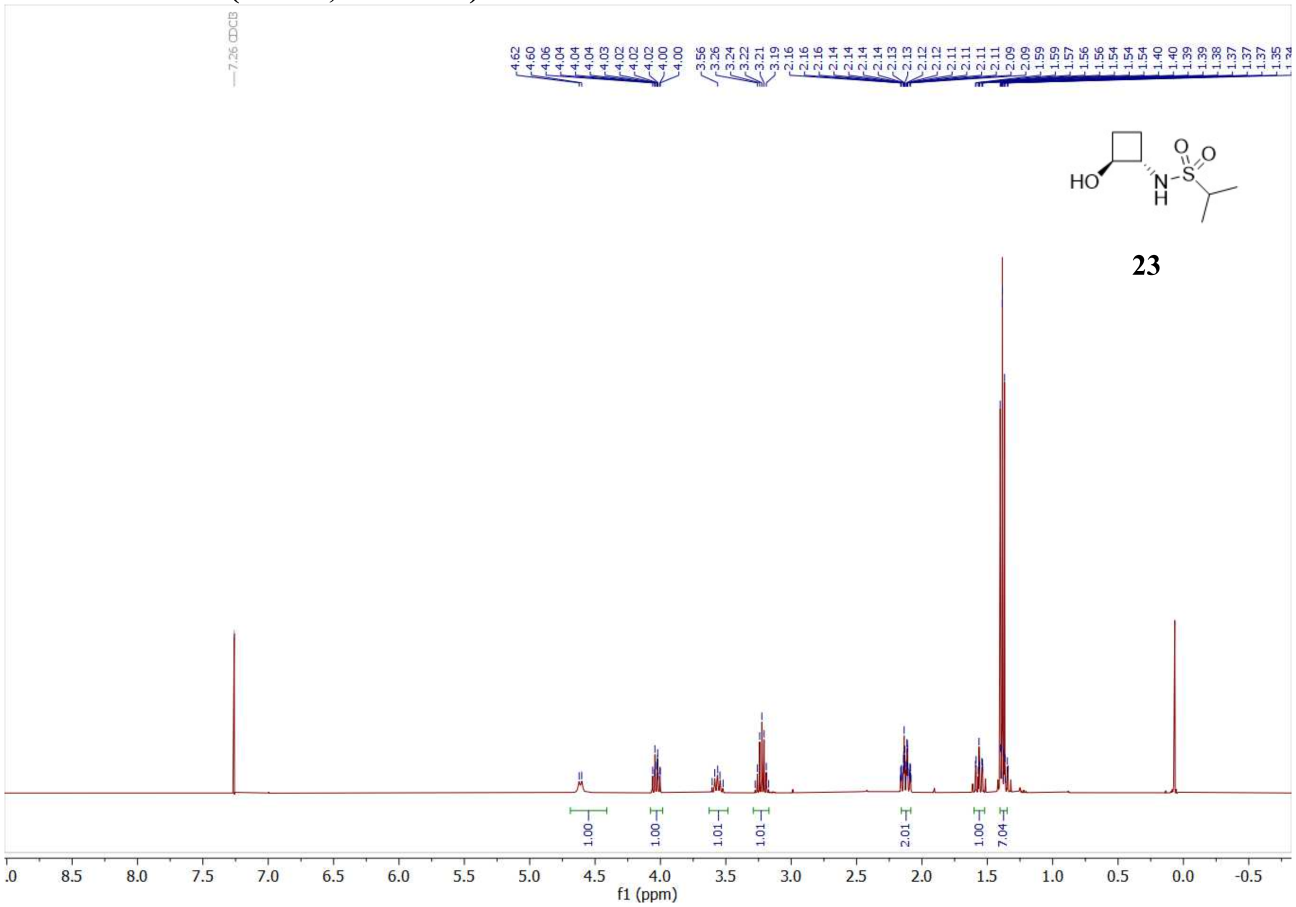

23 ^{13}C NMR (CDCl$_3$, 101 MHz)

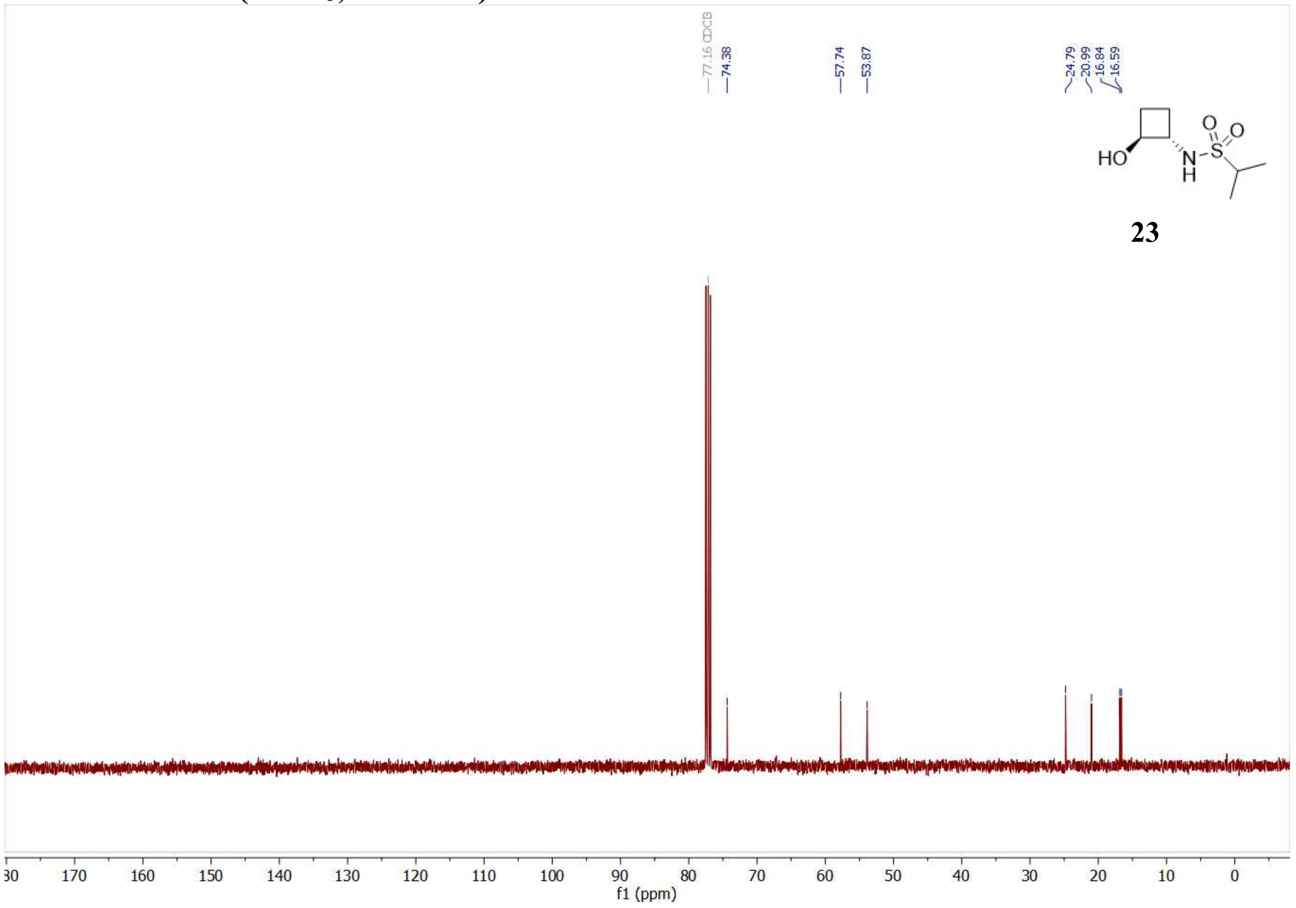

24 & 25 **^{1}H NMR ($CDCl_3$, 500 MHz)**

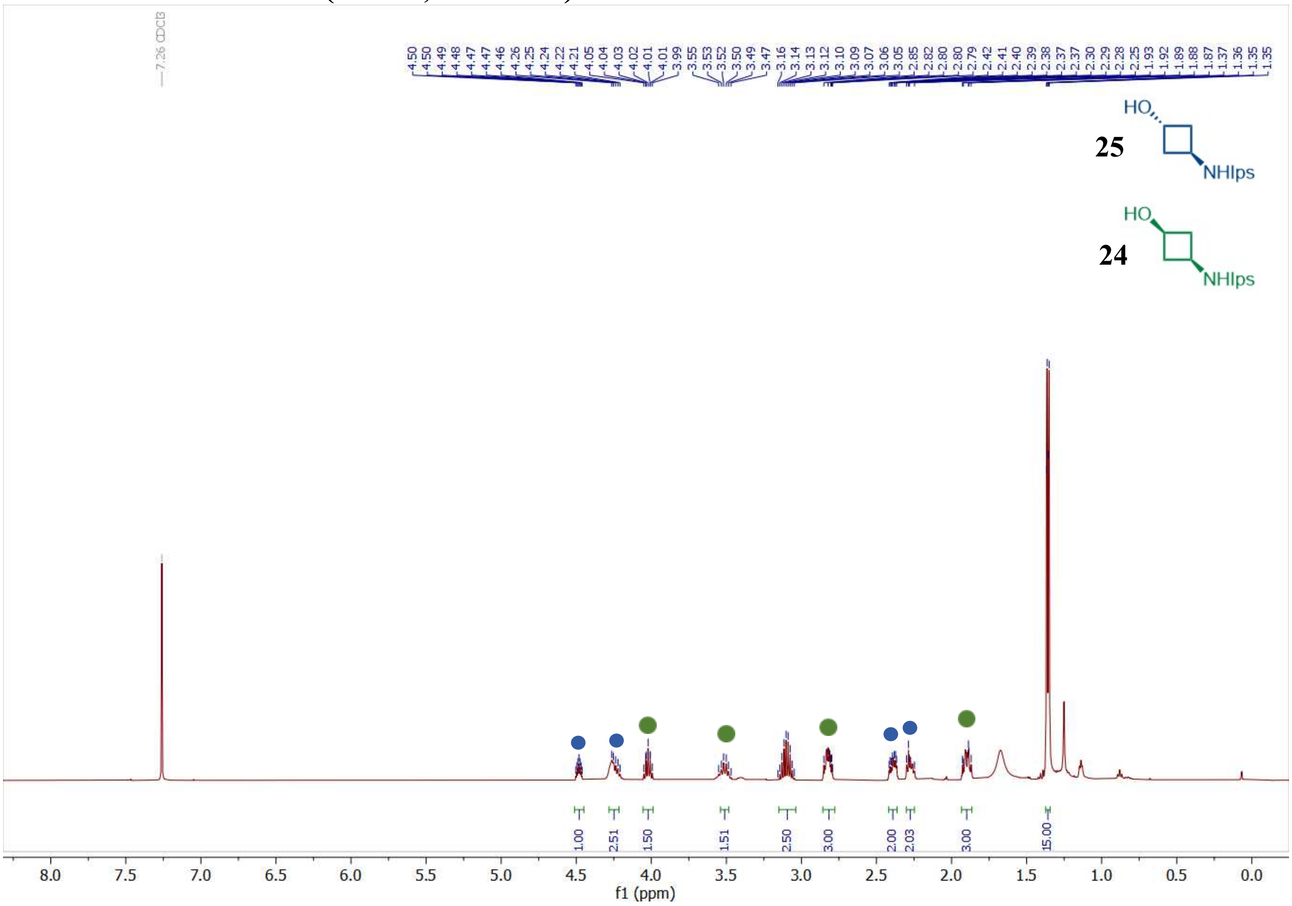

24 & 25 **^{13}C NMR ($CDCl_3$, 126 MHz)**

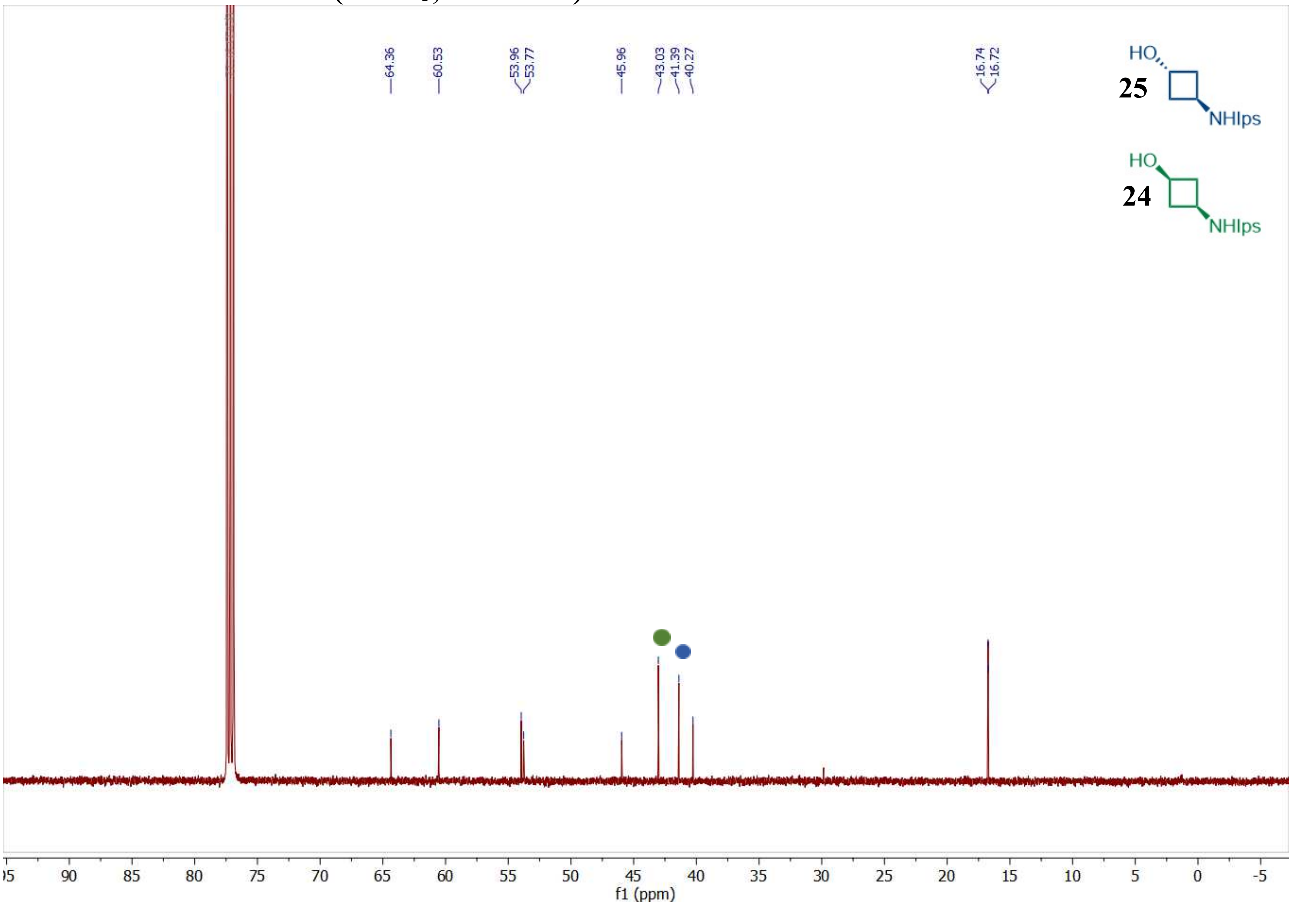

26 ^{1}H NMR ($CDCl_3$, 400 MHz)

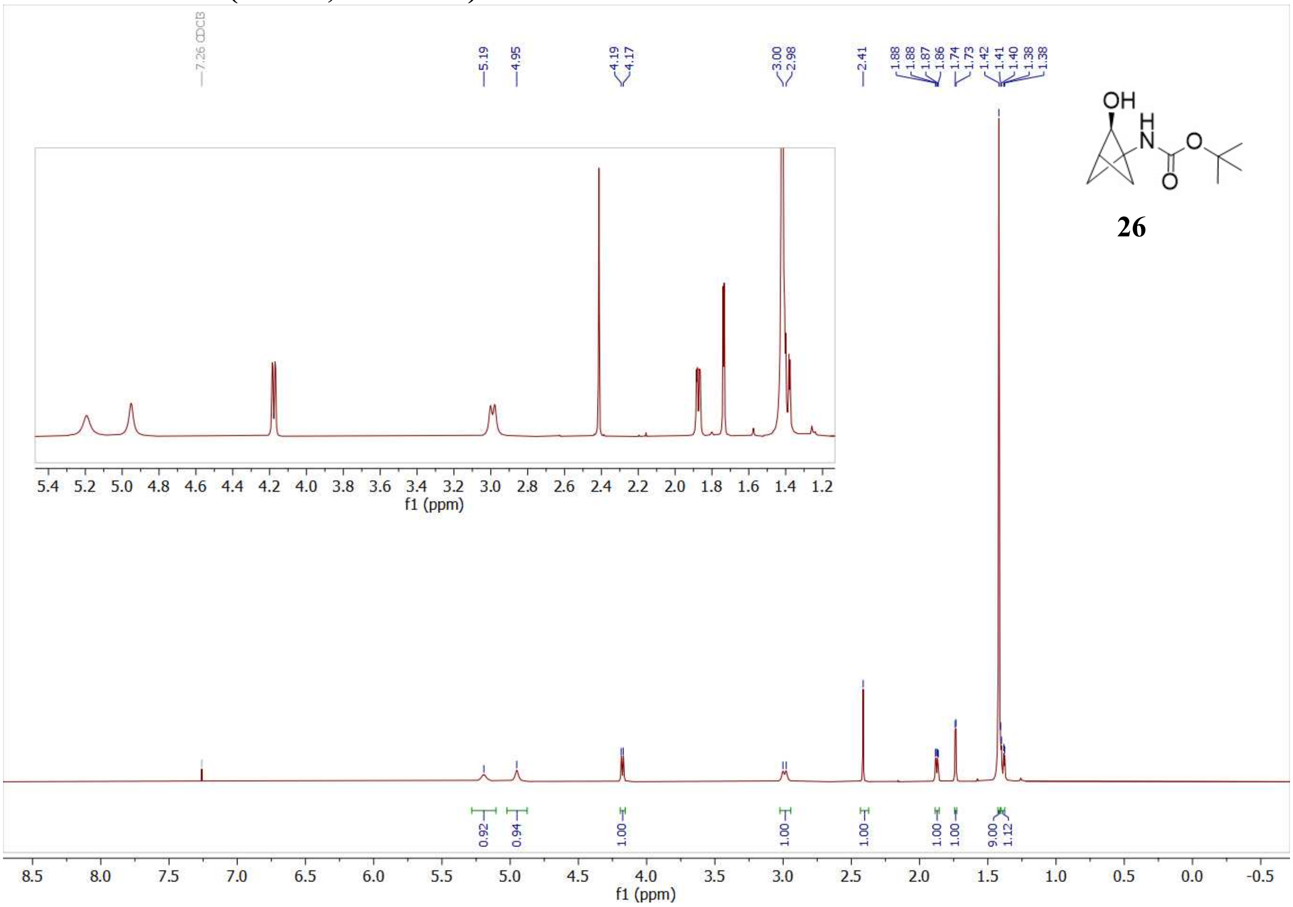

26 ^{13}C NMR ($CDCl_3$, 101 MHz)

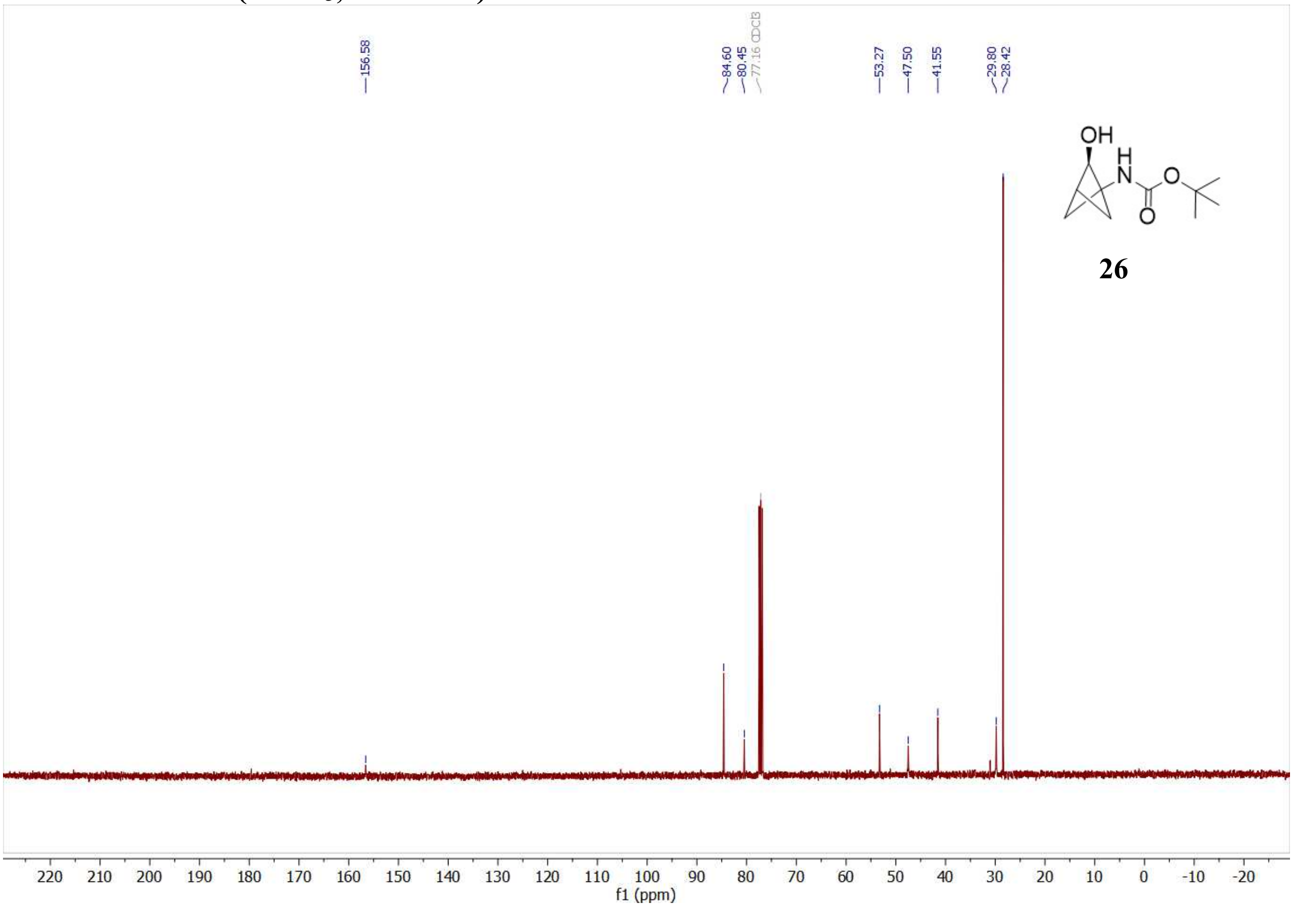

27 ^{1}H NMR (CDCl$_3$, 400 MHz)

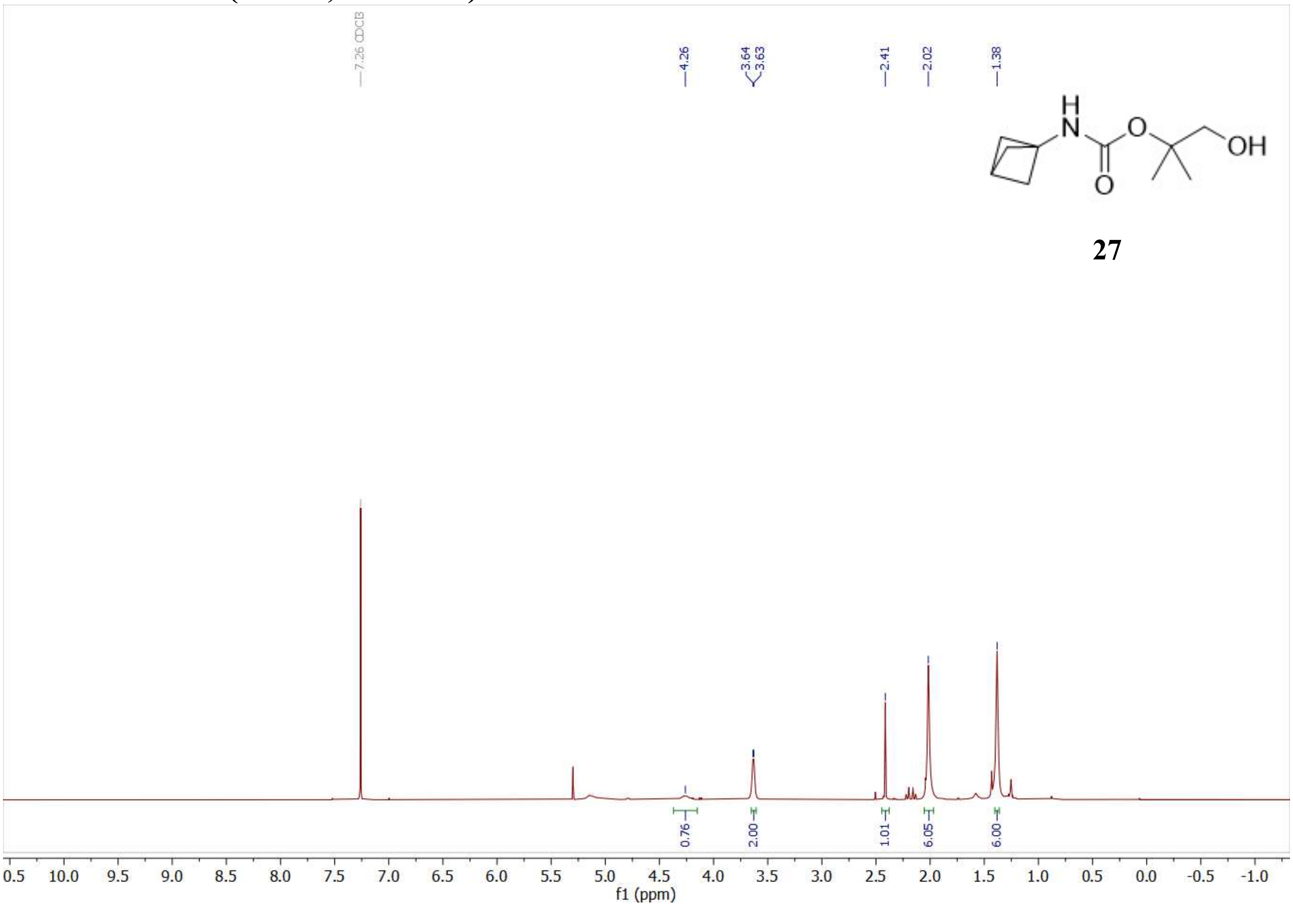

27

27 ^{13}C NMR (CDCl$_3$, 101 MHz)

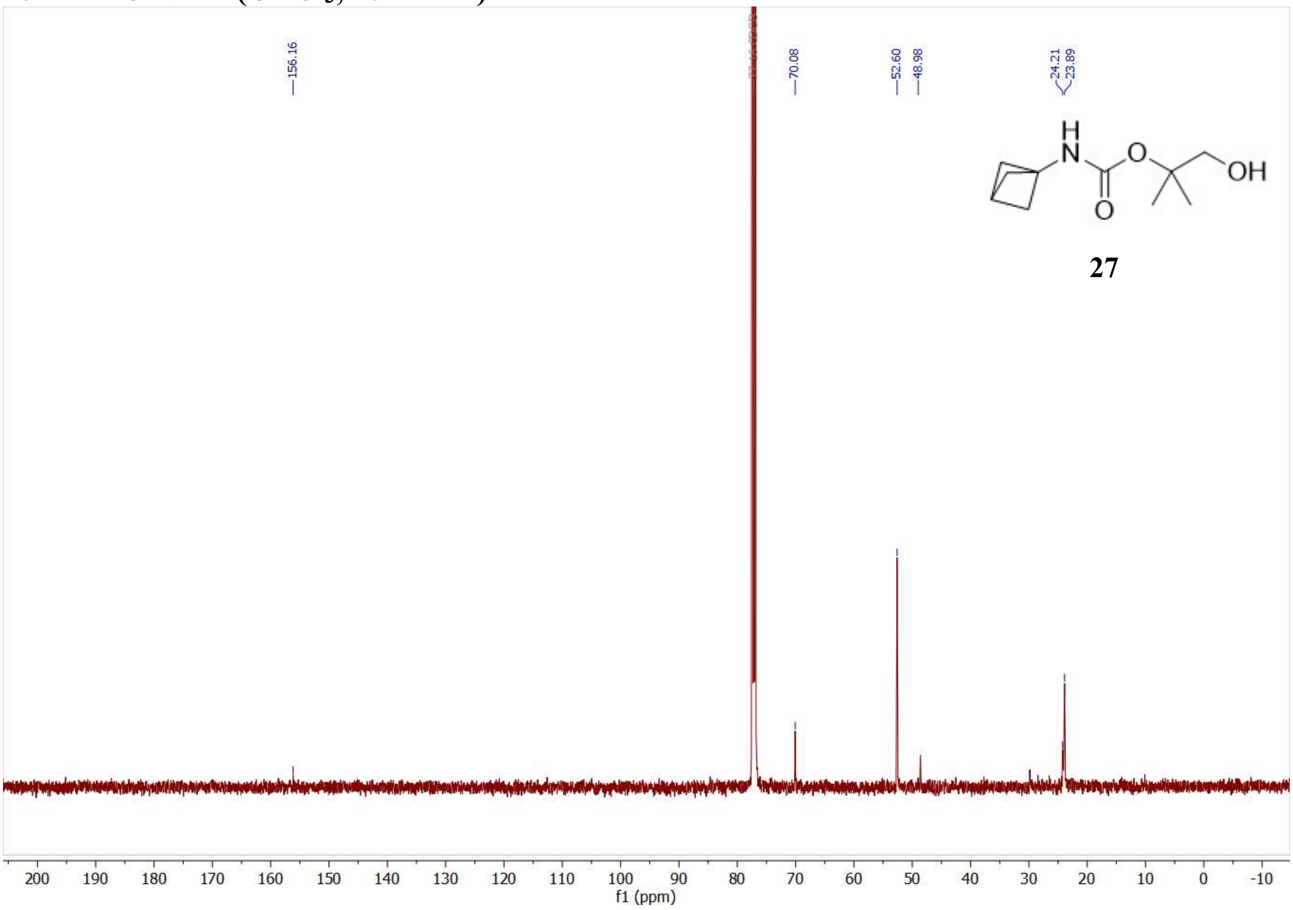

27

28 ^{1}H NMR ($CDCl_3$, 400 MHz)

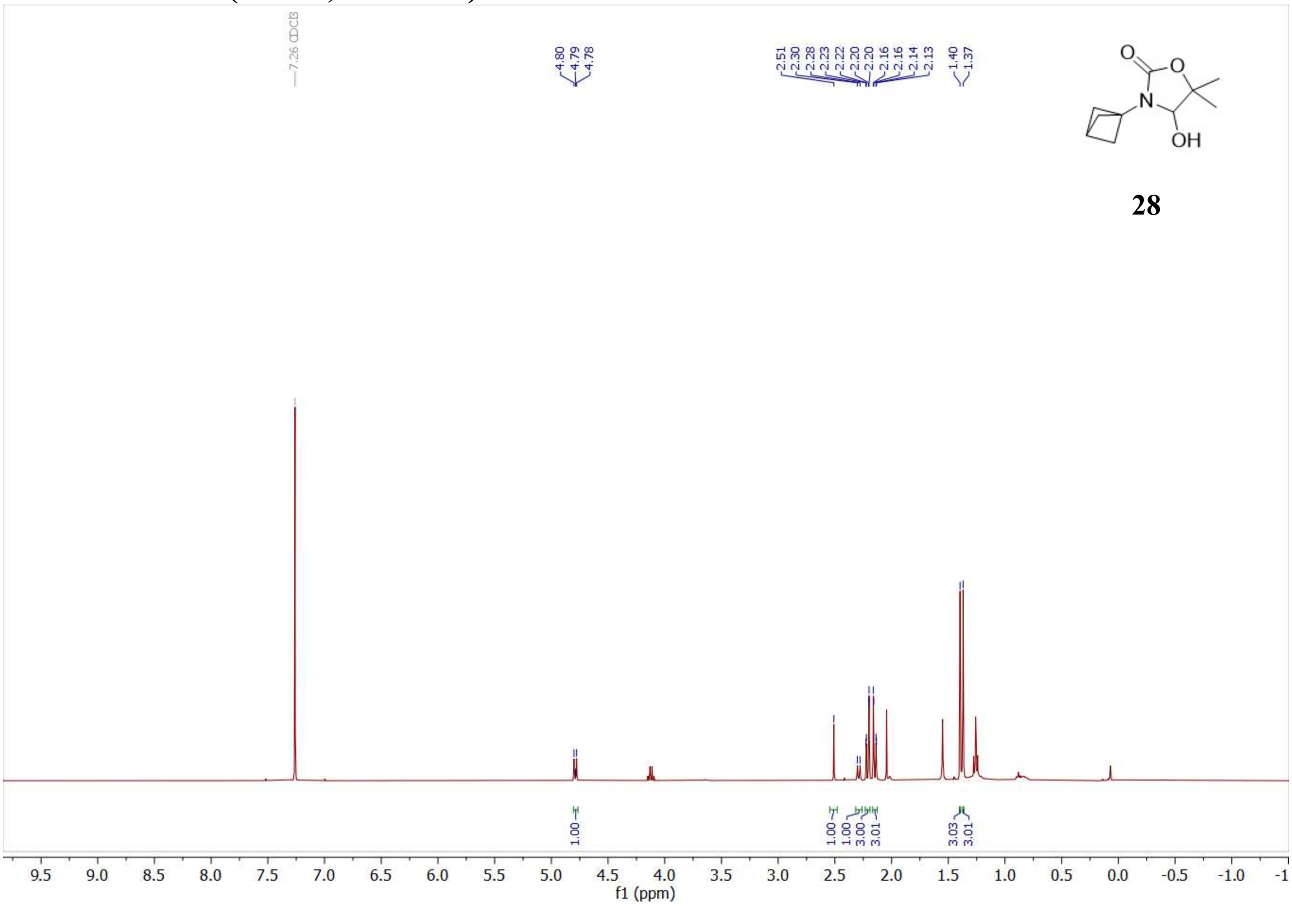

28 ^{13}C NMR ($CDCl_3$, 151 MHz)

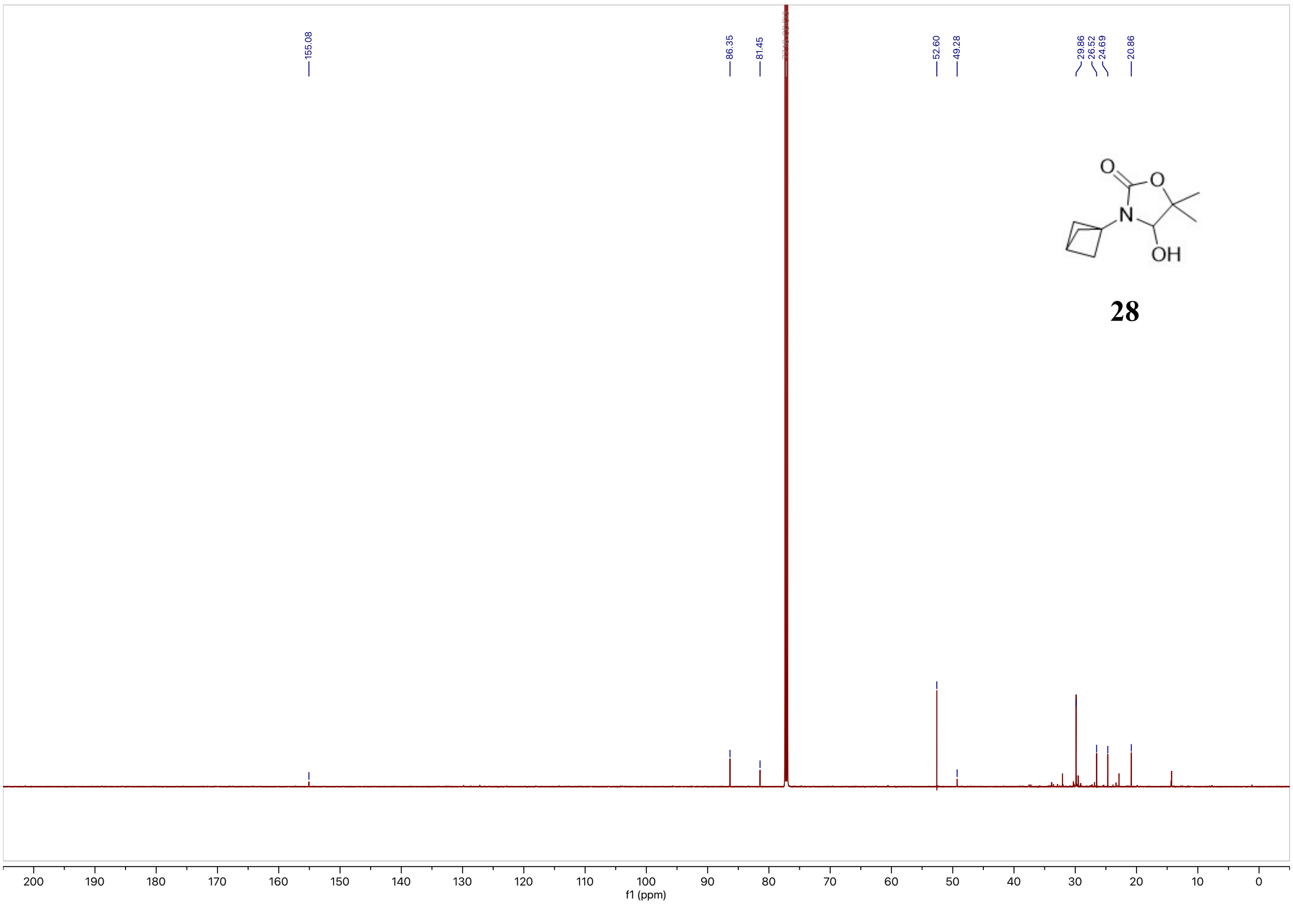

29 ^{1}H NMR (CDCl$_3$, 400 MHz)

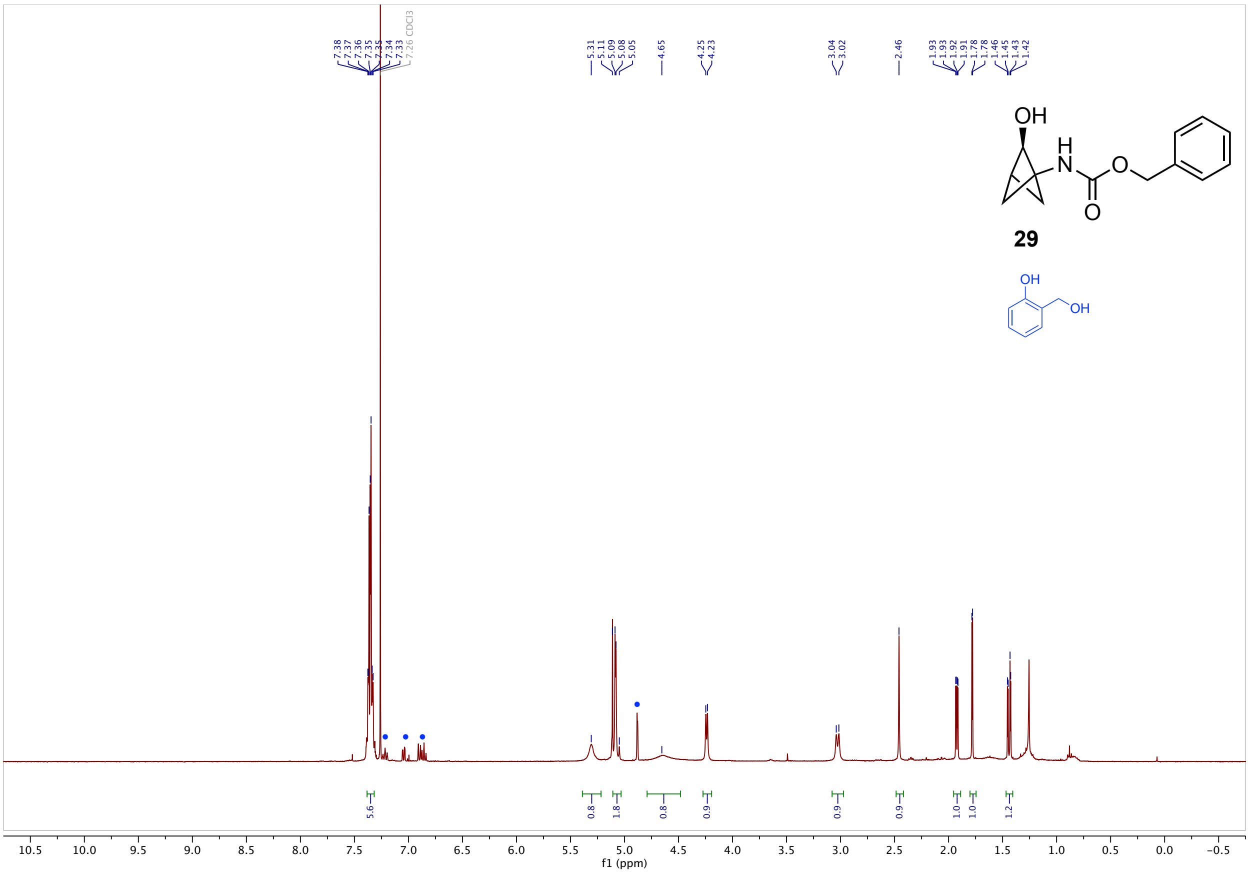

29 ^{13}C NMR (CDCl$_3$, 101 MHz)

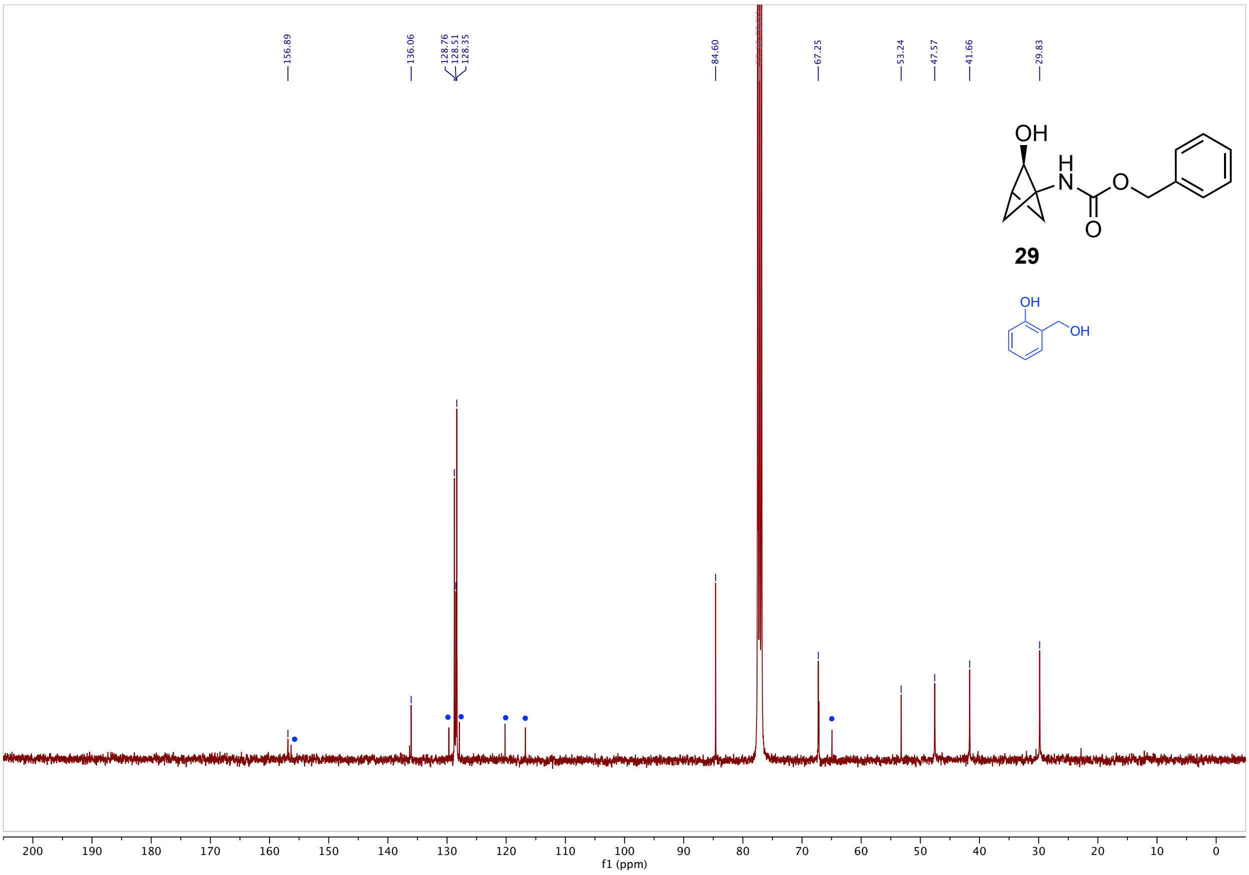

29 ^{1}H NMR (CDCl$_3$, 400 MHz)

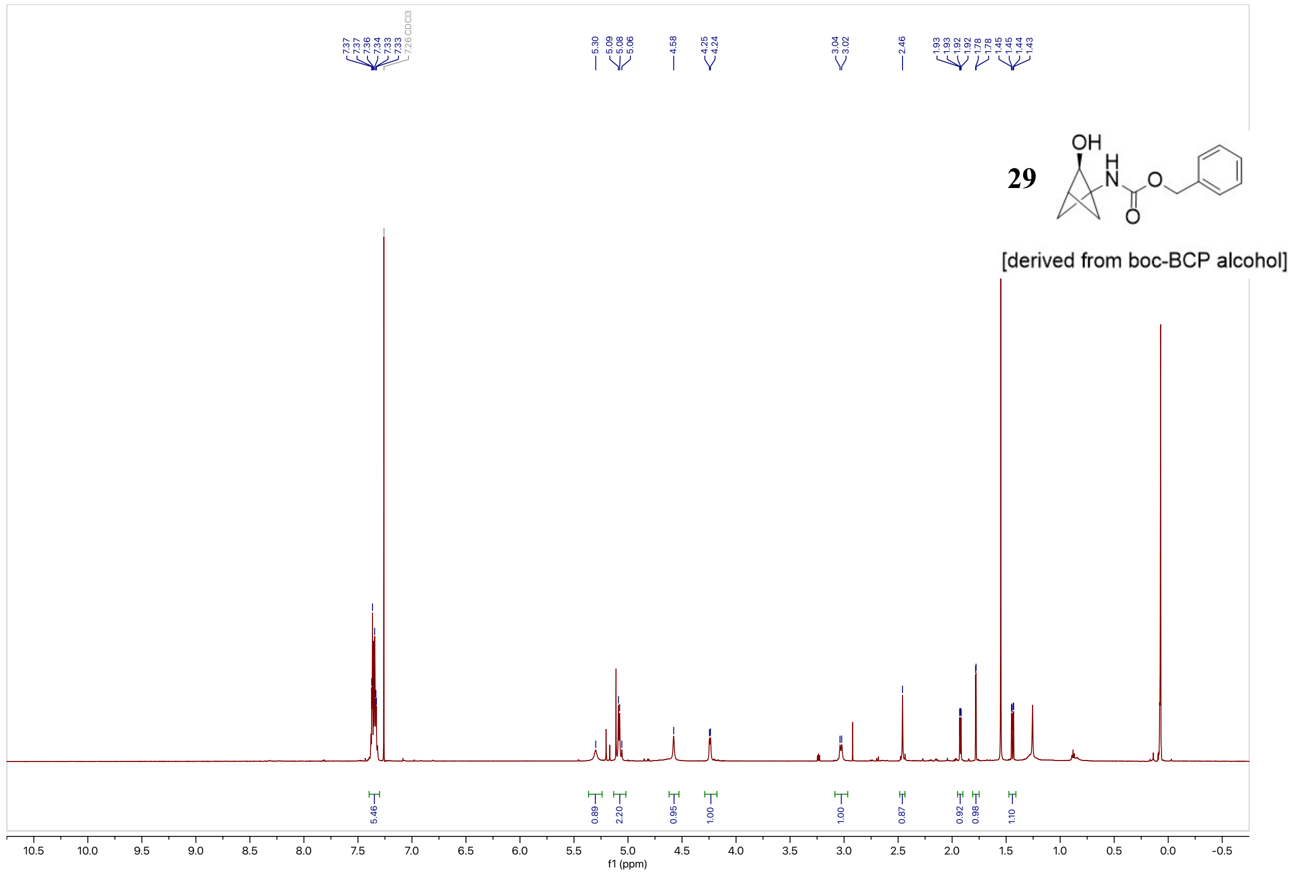

29 ^{13}C NMR (CDCl$_3$, 151 MHz)

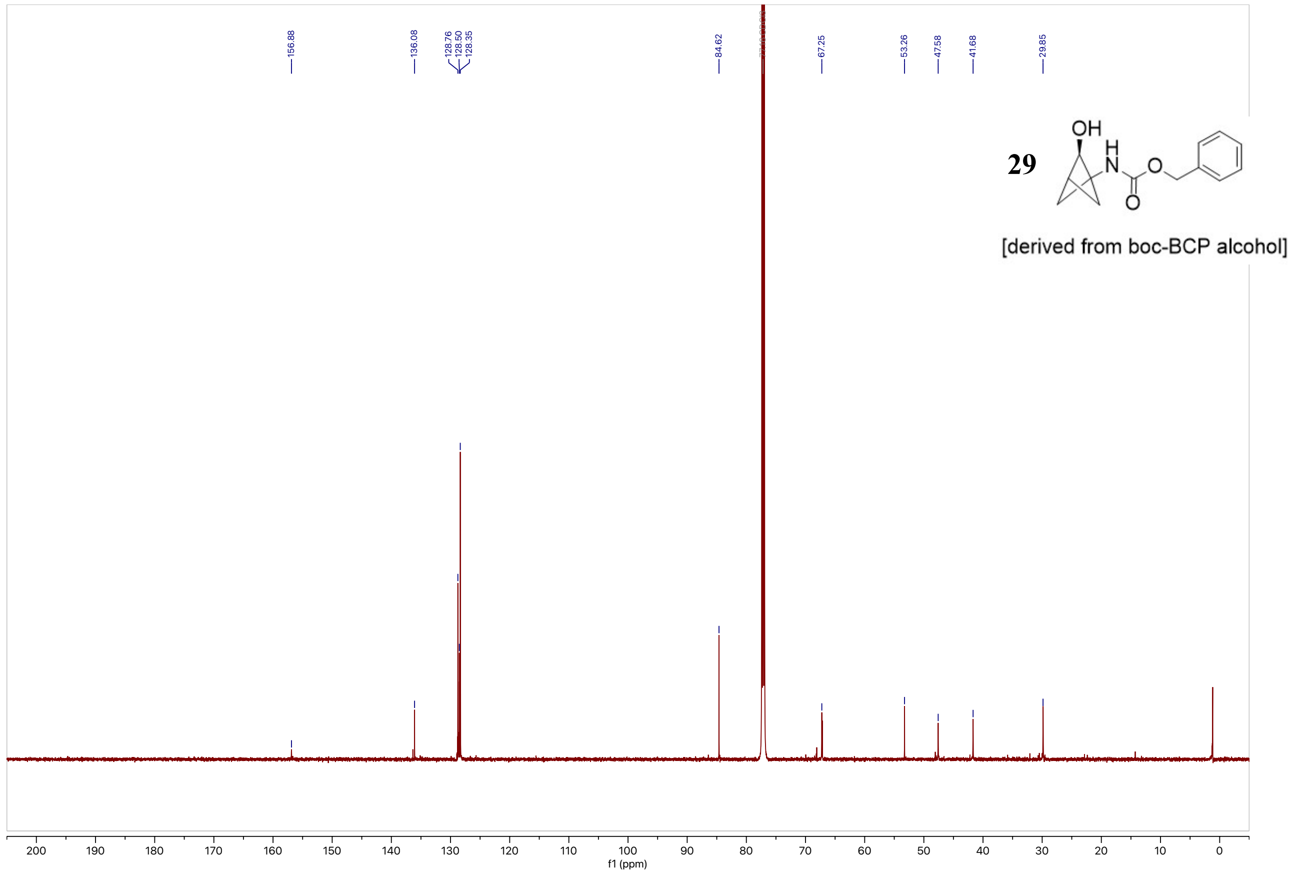

30 ^{1}H NMR ($CDCl_3$, 400 MHz)

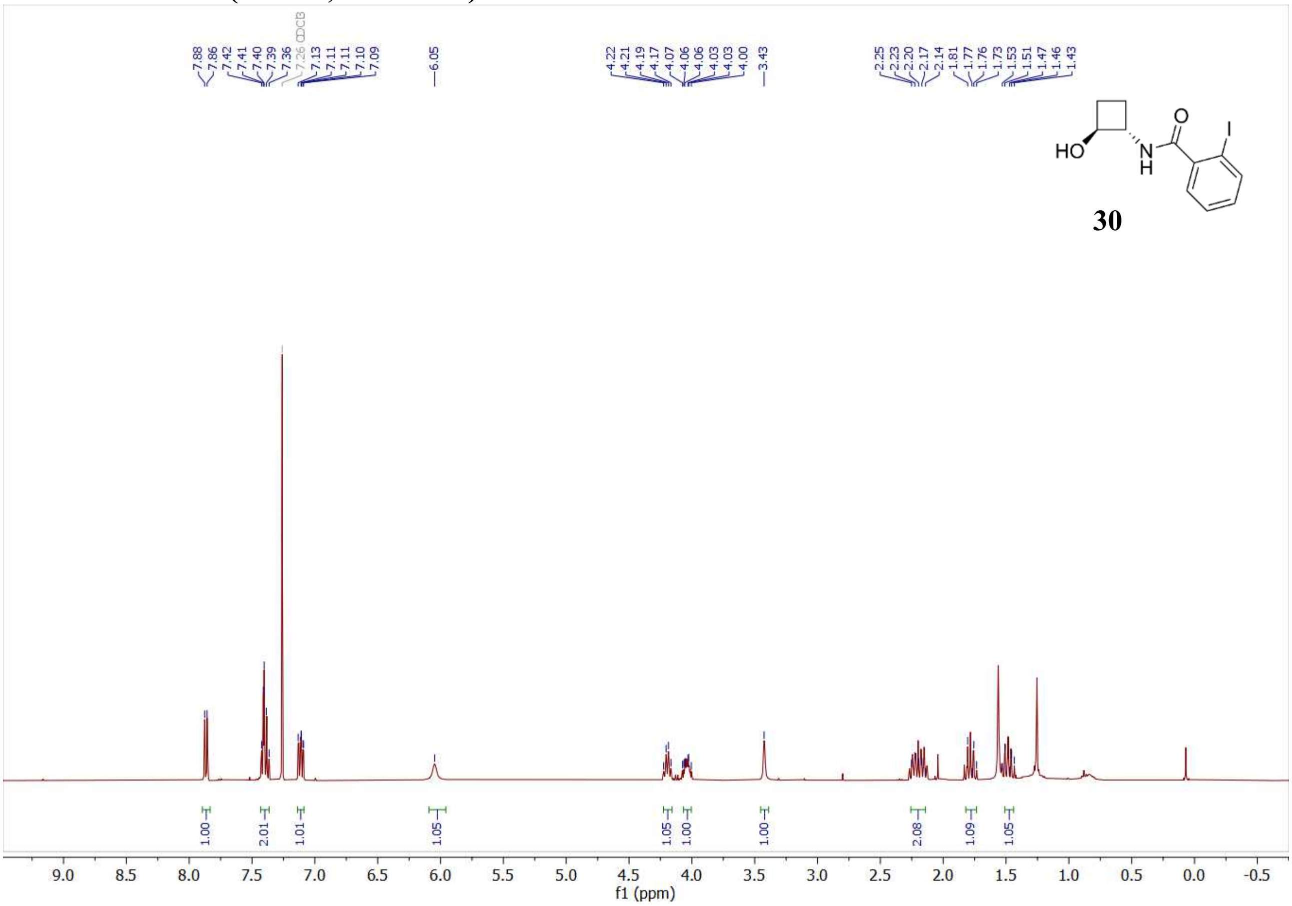

30 ^{13}C NMR ($CDCl_3$, 126 MHz)

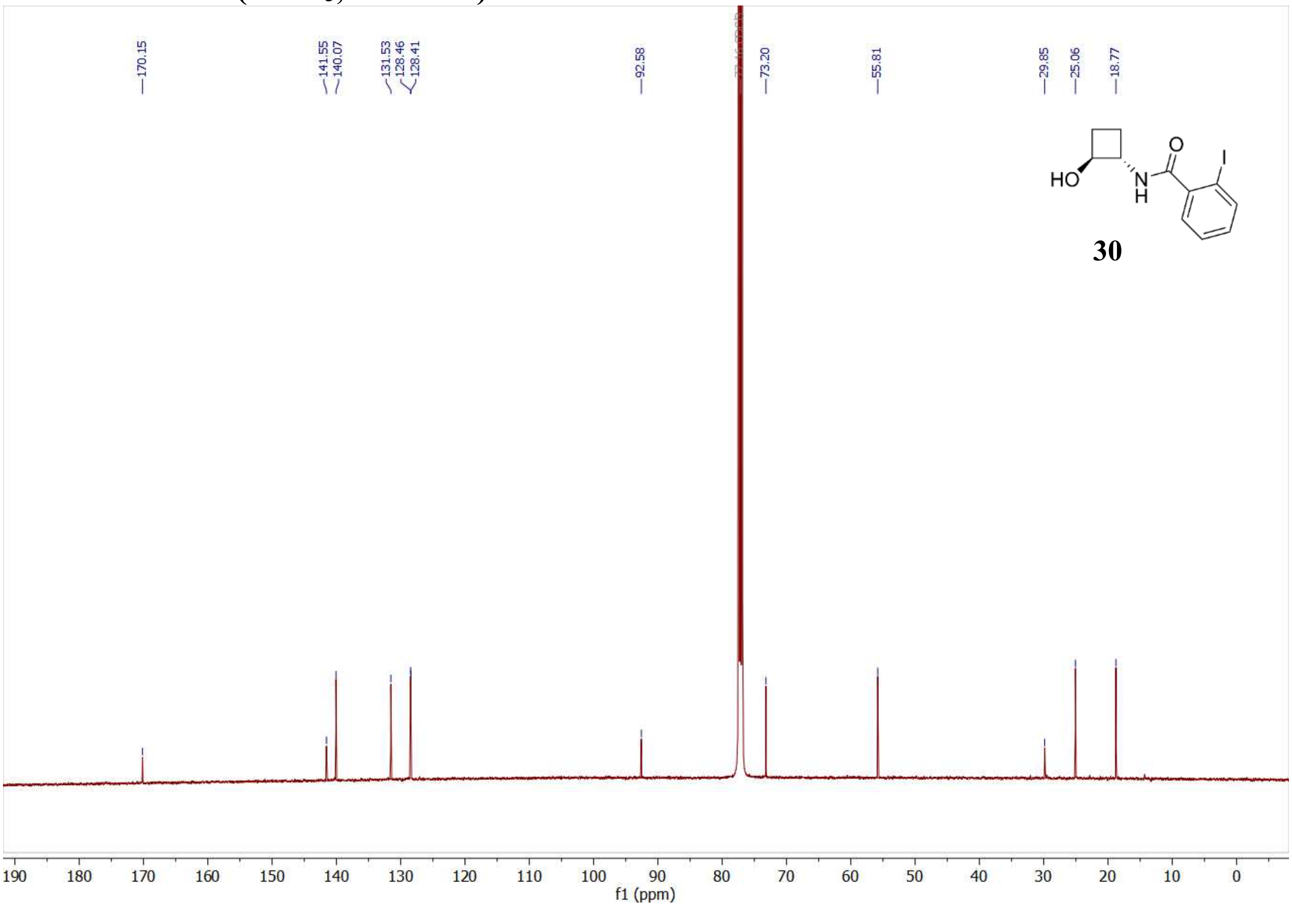

31 ^{1}H NMR ($CDCl_3$, 400 MHz)

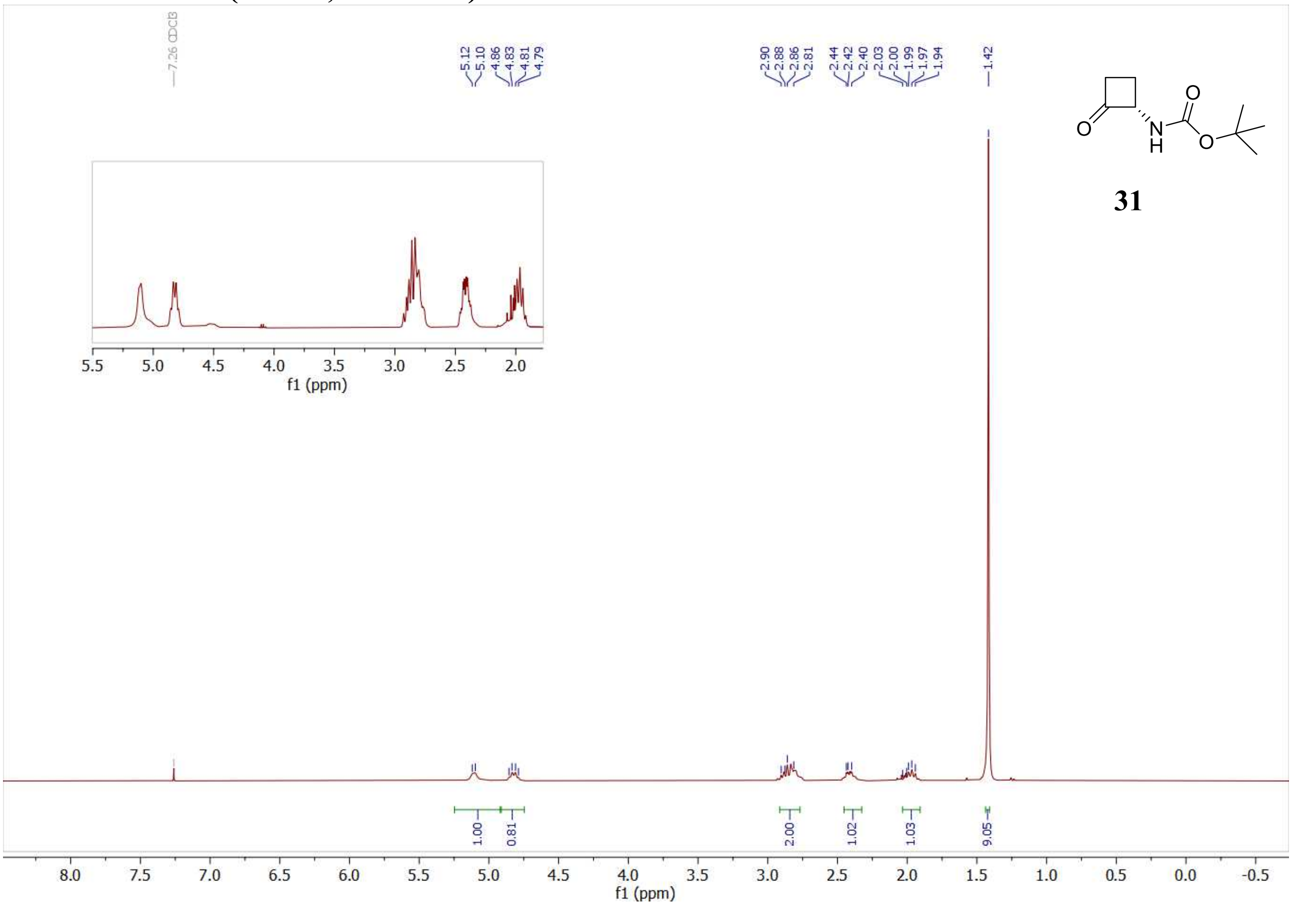

31 ^{13}C NMR ($CDCl_3$, 101 MHz)

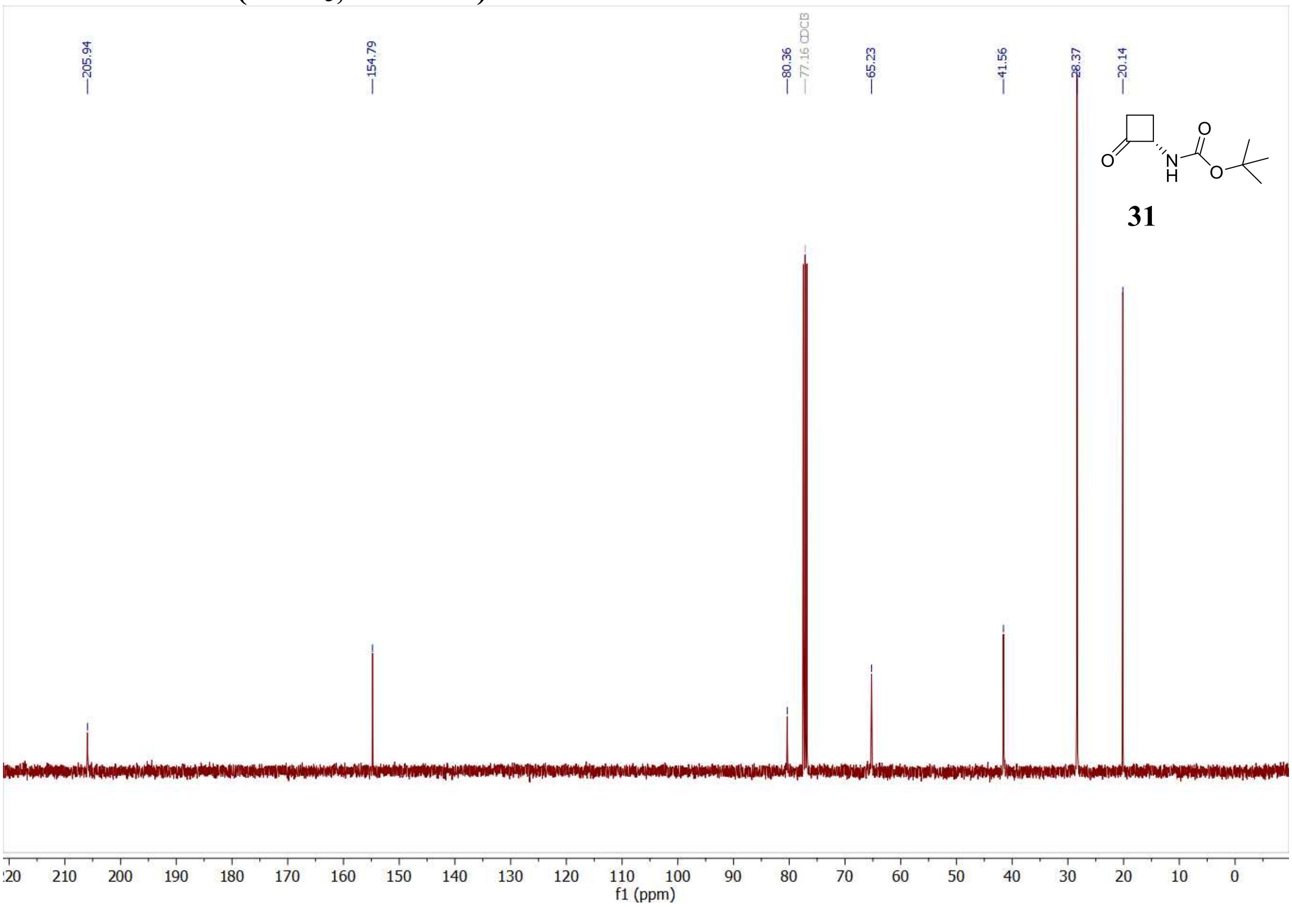

32 ^{1}H NMR (CDCl$_3$, 600 MHz)

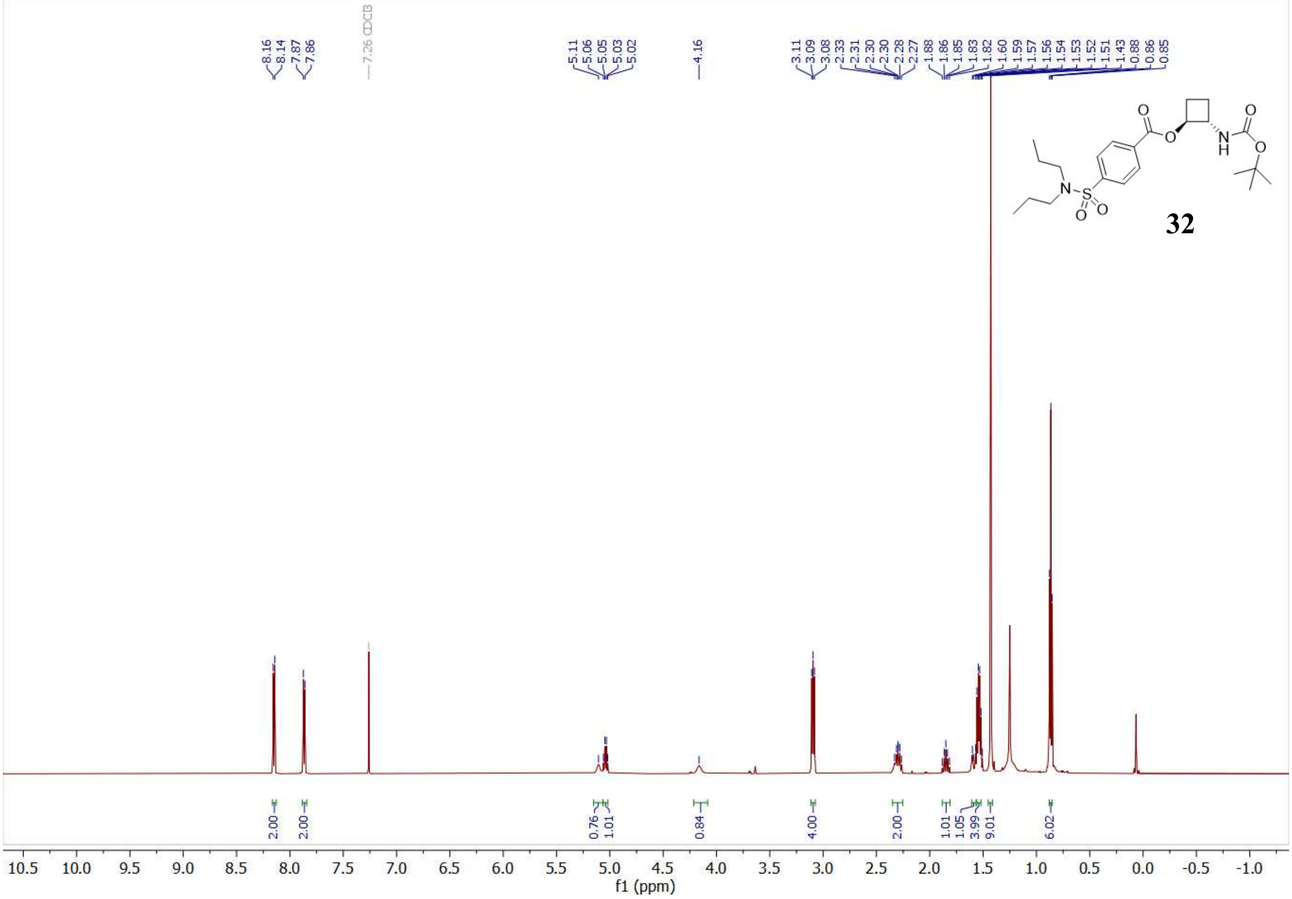

32

32 ^{13}C NMR (CDCl$_3$, 101 MHz)

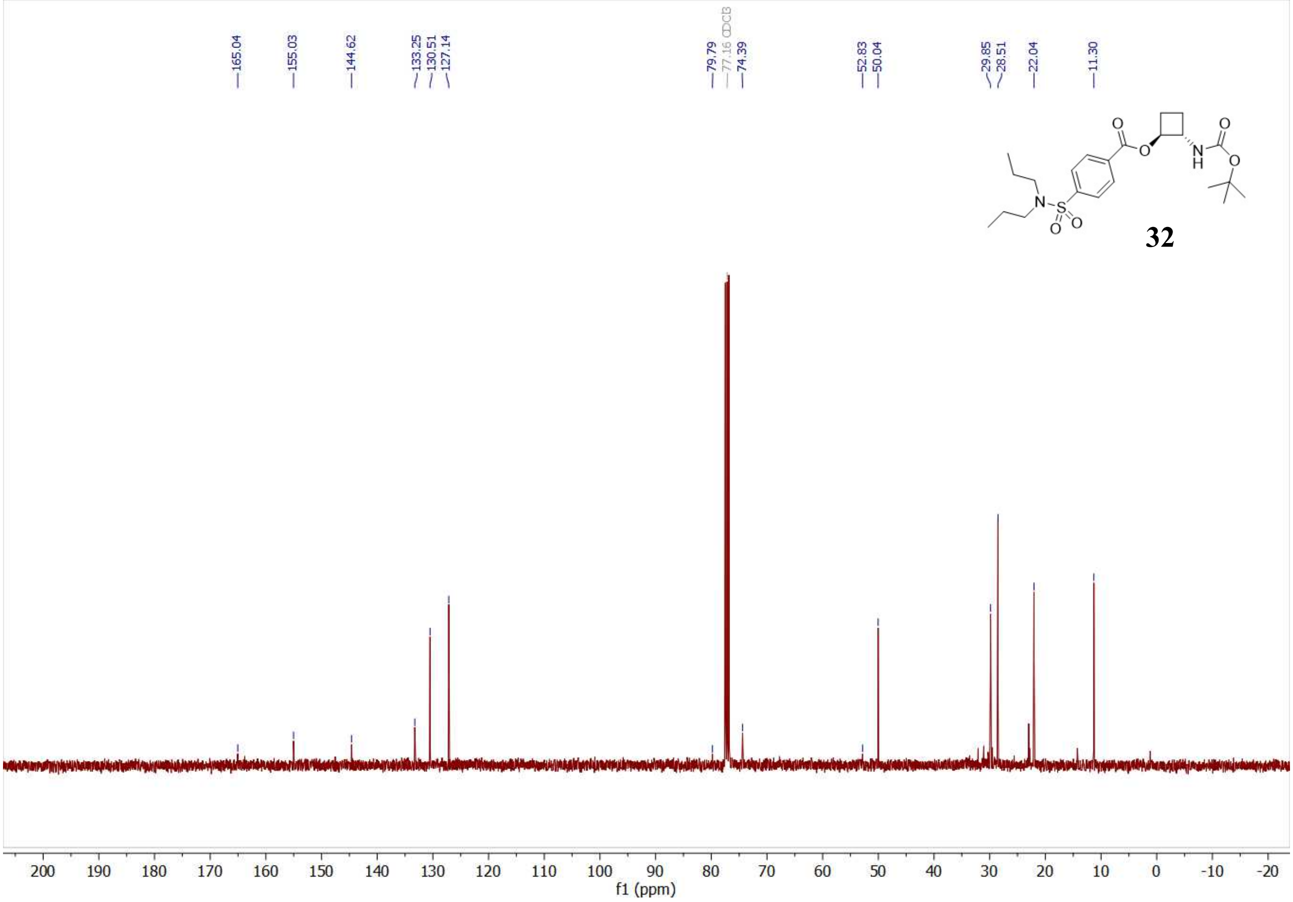

32

33 ^{1}H NMR ($CDCl_3$, 600 MHz)

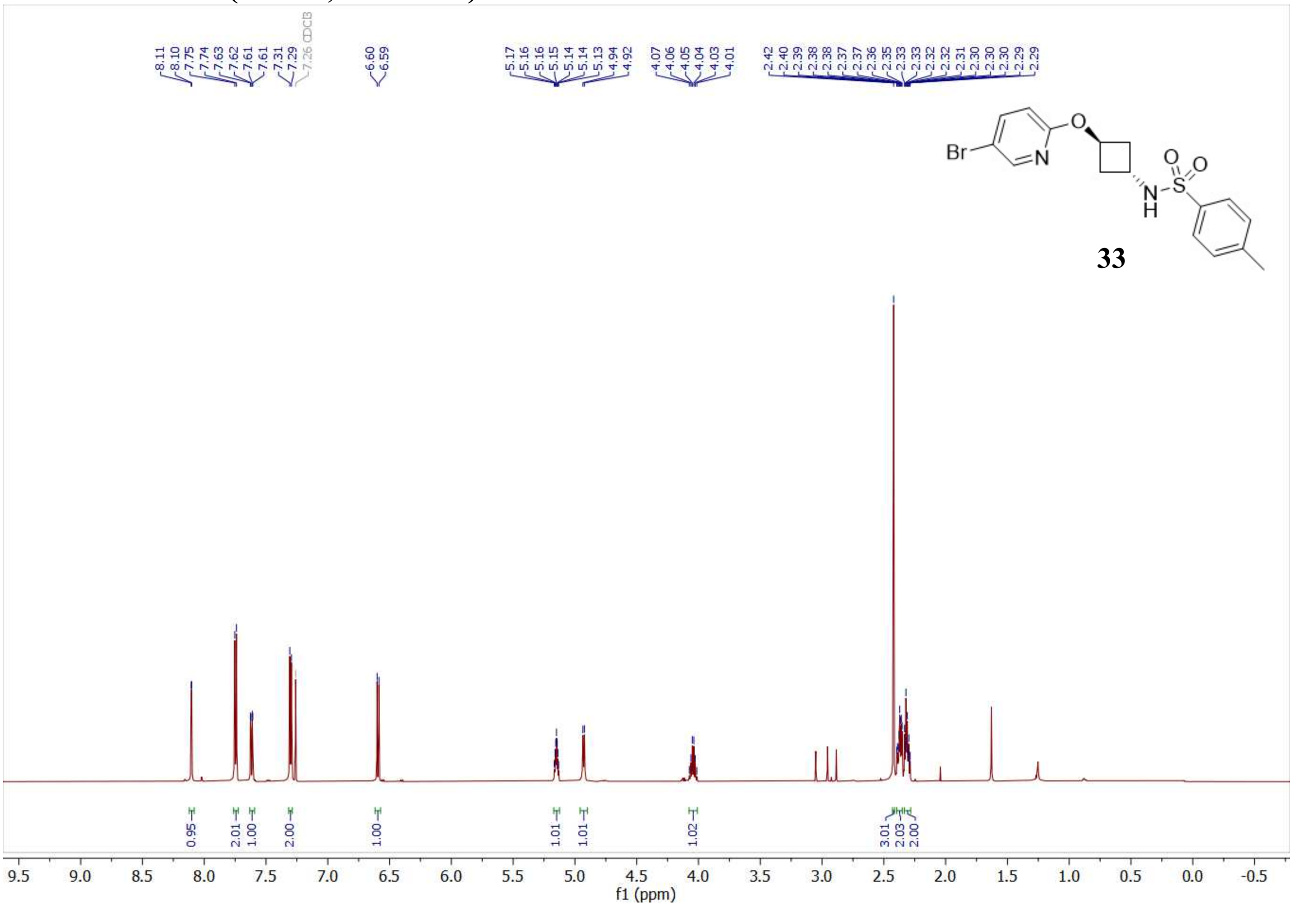

33 ^{13}C NMR ($CDCl_3$, 151 MHz)

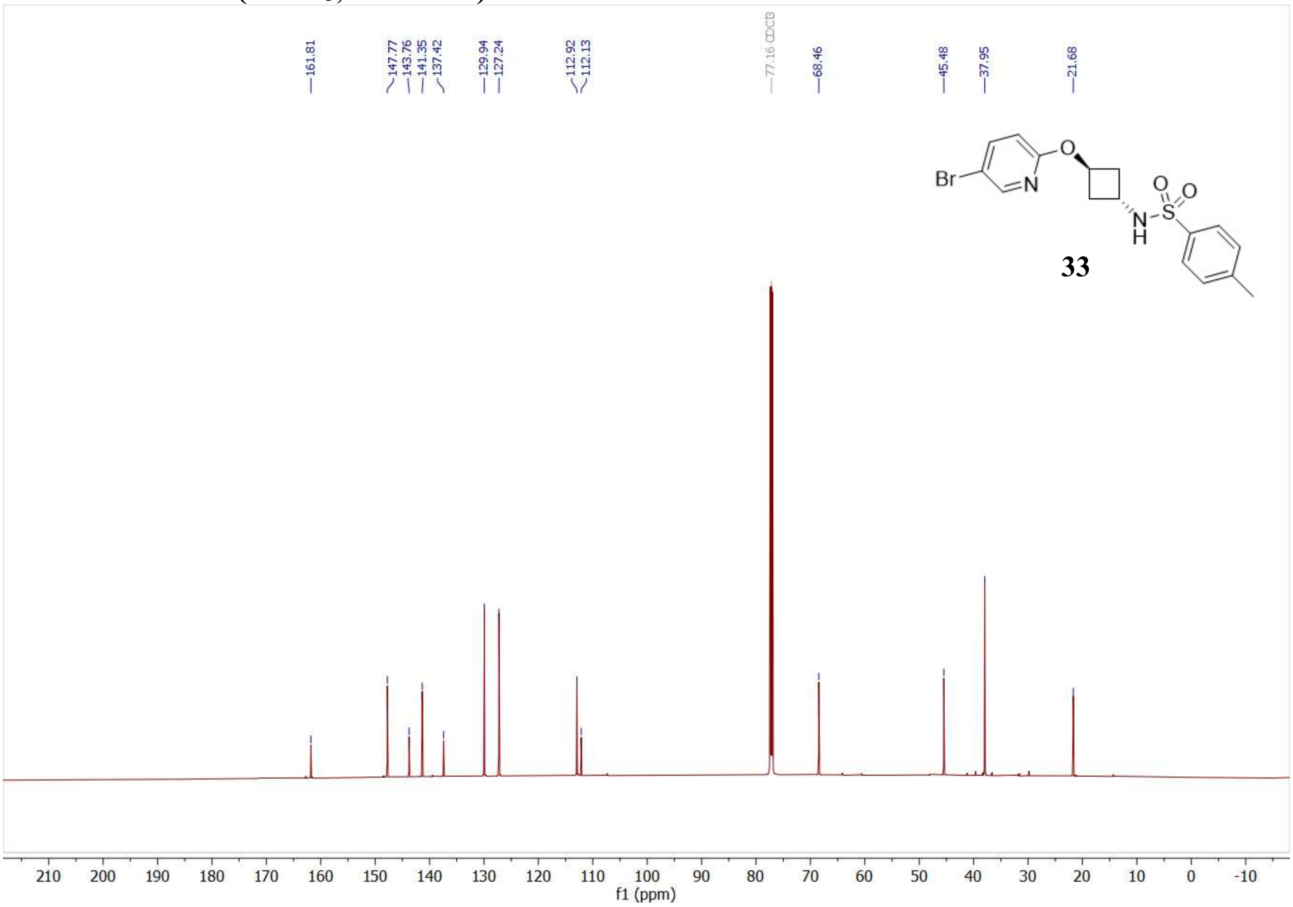

S8 Crystallographic data

Low temperature[11] single crystal X-ray diffraction data were collected using a (Rigaku) Oxford Diffraction SuperNova diffractometer. Raw frame data were reduced using CrysAlisPro and the structures were solved using 'Superflip'[12] before refinement with CRYSTALS;[13] full refinement details are given in the Supporting Information (CIF). Crystallographic data have been deposited with the Cambridge Crystallographic Data Centre (CCDC 2208866–71) and can be obtained via www.ccdc.cam.ac.uk/data_request/cif. Data for compound **18** were collected at both 300 K and 150 K to investigate the possibility of a phase transition but none was found.

Table S8.1: Crystal data and structure refinement for (1*S*,2*S*)-**14**

CCDC code	2208866	
Empirical formula	$C_9H_{17}NO_3$	
Formula weight	187.24	
Temperature	150 K	
Wavelength	1.54184 Å	
Crystal system / Space group	Orthorhombic	$P2_12_12_1$
Unit cell dimensions	a = 10.15740(10) Å	α = 90°
	b = 10.51910(10) Å	β = 90°
	c = 29.1806(2) Å	γ = 90°
Volume	3117.85(5) $Å^3$	
Z	12	
Density (calculated)	1.197 Mg/m^3	
Crystal size	0.20 × 0.19 × 0.12 mm^3	
Independent reflections	6541 [R(int) = 0.032]	
Completeness to theta = 76.470°	99.9 %	
Absorption correction	Semi-empirical from equivalents	
Refinement method	Full-matrix least-squares on F^2	
Data / restraints / parameters	6541 / 0 / 354	
Goodness-of-fit on F^2	1.0030	
Final R indices [I>2sigma(I)]	R1 = 0.0230, wR2 = 0.0633	
R indices (all data)	R1 = 0.0234, wR2 = 0.0638	
Absolute structure parameter	–0.01(2)	

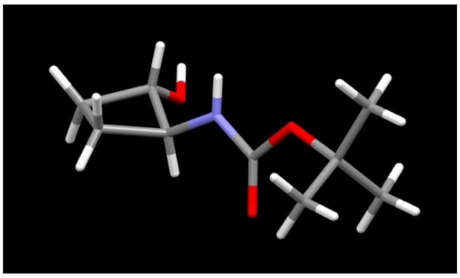

Table S8.2: Crystal data and structure refinement for **18** (300 K)

CCDC code	2208867	
Empirical formula	$C_{11}H_{15}NO_3S$	
Formula weight	241.31	
Temperature	300 K	
Wavelength	1.54184 Å	
Crystal system / Space group	Monoclinic	P 2_1/n
Unit cell dimensions	a = 6.67140(10) Å	α = 90°
	b = 24.4956(4) Å	β = 89.9743(17)°
	c = 7.22530(10) Å	γ = 90°
Volume	1180.76(3) Å^3	
Z	4	
Density (calculated)	1.357 Mg/m^3	
Crystal size	0.25 × 0.08 × 0.06 mm^3	
Independent reflections	2449 [R(int) = 0.036]	
Completeness to theta = 74.882°	99.5 %	
Absorption correction	Semi-empirical from equivalents	
Refinement method	Full-matrix least-squares on F^2	
Data / restraints / parameters	2446 / 40 / 198	
Goodness-of-fit on F^2	1.0410	
Final R indices [I>2sigma(I)]	R1 = 0.0786, wR2 = 0.1986	
R indices (all data)	R1 = 0.0797, wR2 = 0.1991	

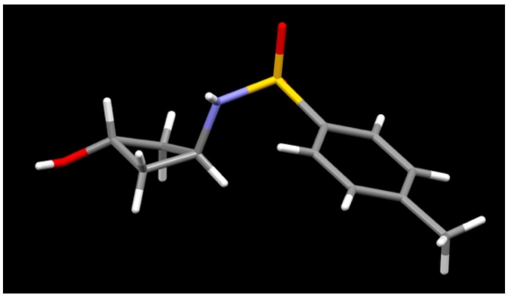

Table S8.3: Crystal data and structure refinement for **18** (150 K)

CCDC code	2208868	
Empirical formula	$C_{11}H_{15}NO_3S$	
Formula weight	241.31	
Temperature	150 K	
Wavelength	1.54184 Å	
Crystal system / Space group	Monoclinic	P 2_1/n
Unit cell dimensions	a = 6.62580(10) Å	α = 90°
	b = 24.4743(3) Å	β = 90.2022(11)°
	c = 7.09120(10) Å	γ = 90°
Volume	1149.91(3) $Å^3$	
Z	4	
Density (calculated)	1.394 Mg/m^3	
Crystal size	0.27 × 0.24 × 0.22 mm^3	
Independent reflections	2408 [R(int) = 0.019]	
Completeness to theta = 76.800°	99.6 %	
Absorption correction	Semi-empirical from equivalents	
Refinement method	Full-matrix least-squares on F^2	
Data / restraints / parameters	2403 / 152 / 209	
Goodness-of-fit on F^2	0.9818	
Final R indices [I>2sigma(I)]	R1 = 0.0825, wR2 = 0.0245	
R indices (all data)	R1 = 0.0826, wR2 = 0.0245	

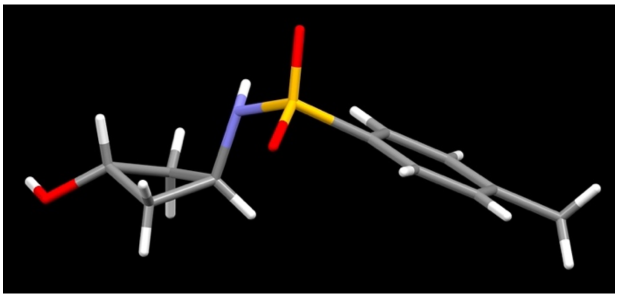

Table S8.4: Crystal data and structure refinement for (±)-**21**

CCDC code	2208869	
Empirical formula	$C_{11}H_{15}NO_3S$	
Formula weight	241.31	
Temperature	150 K	
Wavelength	1.54184 Å	
Crystal system / Space group	Monoclinic	P 2_1/n
Unit cell dimensions	a = 6.53200(10) Å	α = 90°
	b = 7.39220(10) Å	β = 94.7894(11)°
	c = 24.2354(3) Å	γ = 90°
Volume	1166.14(3) $Å^3$	
Z	4	
Density (calculated)	1.374 Mg/m^3	
Crystal size	0.26 × 0.20 × 0.07 mm^3	
Independent reflections	2428 [R(int) = 0.028]	
Completeness to theta = 74.681°	99.5 %	
Absorption correction	Semi-empirical from equivalents	
Refinement method	Full-matrix least-squares on F^2	
Data / restraints / parameters	2428 / 0 / 145	
Goodness-of-fit on F^2	1.0088	
Final R indices [I>2sigma(I)]	R1 = 0.0327, wR2 = 0.0934	
R indices (all data)	R1 = 0.0336, wR2 = 0.0944	

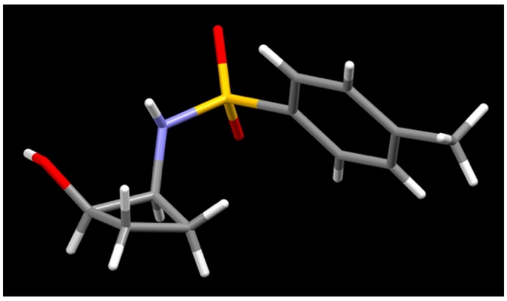

Table S8.5: Crystal data and structure refinement for (2*R*)-**26**

CCDC code	2208870	
Empirical formula	$C_{10}H_{17}NO_3$	
Formula weight	199.25	
Temperature	150 K	
Wavelength	1.54184 Å	
Crystal system / Space group	Orthorhombic	$P2_12_12_1$
Unit cell dimensions	a = 6.06410(10) Å	α = 90°
	b = 9.73480(10) Å	β = 90°
	c = 18.3114(2) Å	γ = 90°
Volume	1080.97(2) Å^3	
Z	4	
Density (calculated)	1.224 Mg/m^3	
Crystal size	0.24 × 0.21 × 0.08 mm^3	
Independent reflections	2260 [R(int) = 0.032]	
Completeness to theta = 76.063°	99.9 %	
Absorption correction	Semi-empirical from equivalents	
Refinement method	Full-matrix least-squares on F^2	
Data / restraints / parameters	2260 / 0 / 129	
Goodness-of-fit on F^2	1.0043	
Final R indices [I>2sigma(I)]	R1 = 0.0306, wR2 = 0.0855	
R indices (all data)	R1 = 0.0313, wR2 = 0.0866	
Absolute structure parameter	–0.05(4)	

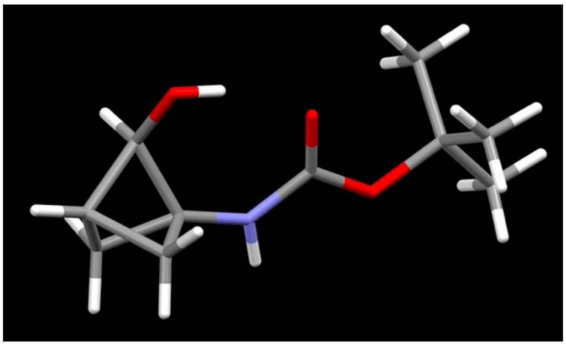

Table S8.6: Crystal data and structure refinement for (2*R*)-**26**/(2*S*)-**26**, 70:30

Acquired by Dr. Ruiyao Wang. A crystal of the compound was mounted on a glass fiber with grease and cooled to –93 °C in a stream of nitrogen gas from Cobra II. Data collection was performed on a Bruker D8 VENTURE Photon II diffractometer with graphite-monochromated Mo K_α radiation at 180 K, operating at 50 kV and 30 mA over θ ranges of 3.29 ~ 30.517°. No significant decay was observed during the data collection. Data were processed on a PC using the Bruker AXS Crystal Structure Analysis Package (Bruker *APEX2*) for data collection, cell refinement, and data reduction. Program(s) used to solve structure: *SHELXS2013*;[14] program(s) used to refine structure: *SHELXL2014*/7.[15] Neutral atom scattering factors were taken from Cromer and Waber.[16] Crystallographic data have been deposited with the Cambridge Crystallographic Data Centre (CCDC 226900, 226901) and can be obtained via www. ccdc.cam.ac.uk/data_request/cif.

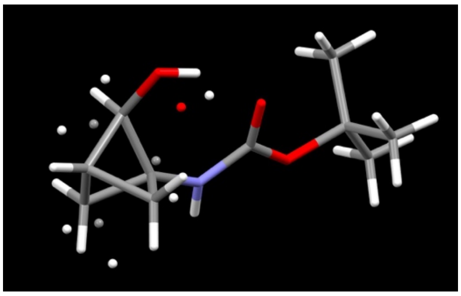

CCDC code	2269000	
Empirical formula	$C_{10}H_{17}NO_3$	
Formula weight	199.25	
Temperature	180 K	
Wavelength	0.71073 Å	
Crystal system / Space group	Monoclinic	$P2_1$
Unit cell dimensions	a = 6.1088(3) Å	α = 90°
	b = 9.8460(5) Å	β = 90.482(2)°
	c = 18.3621(8) Å	γ = 90°
Volume	1104.39(9) Å^3	
Z	4	
Density (calculated)	1.198 Mg/m^3	
Crystal size	0.35 × 0.04 × 0.04 mm^3	
Independent reflections	2260 [R(int) = 0.032]	
Completeness to theta = 25.242°	99.8 %	
Absorption correction	Semi-empirical from equivalents	
Refinement method	Full-matrix least-squares on F^2	
Data / restraints / parameters	6618 / 1 / 279	
Goodness-of-fit on F^2	1.054	
Final R indices [I>2sigma(I)]	R1 = 0.0525, wR2 = 0.1127	
R indices (all data)	R1 = 0.0667, wR2 = 0.1199	
Absolute structure parameter	0.0(4)	

Table S8.7: Crystal data and structure refinement for (2*R*)-**26** (*er* > 99:1) from the same crystal crop as above (Table S8.6)

Acquired by Dr. Ruiyao Wang. A crystal of the compound was mounted on a glass fiber with grease. Data collection was performed on a Rigaku XtaLAB Synergy, Dualflex, HyPix diffractometer at room-temperature using Cu K_α radiation. No significant decay was observed during the data collection. CrysAlisPro 1.171.42.79a was used for data collection, cell refinement, and data reduction. Other details as Table S8.6.

CCDC code	2269001	
Empirical formula	$C_{10}H_{17}NO_3$	
Formula weight	199.25	
Temperature	299(2) K	
Wavelength	1.54184 Å	
Crystal system / Space group	Orthorhombic	$P2_12_12_1$
Unit cell dimensions	a = 6.10680(10) Å	α = 90°
	b = 9.90590(10) Å	β = 90°
	c = 18.57777(3) Å	γ = 90°
Volume	1123.83(3) Å^3	
Z	4	
Density (calculated)	1.178 Mg/m^3	
Crystal size	0.20 × 0.08 × 0.08 mm^3	
Independent reflections	2336 [R(int) = 0.0178]	
Completeness to theta = 67.684°	99.9 %	
Absorption correction	Multi-scan	
Refinement method	Full-matrix least-squares on F^2	
Data / restraints / parameters	2336 / 0 / 134	
Goodness-of-fit on F^2	1.060	
Final R indices [I>2sigma(I)]	R1 = 0.0377, wR2 = 0.1112	
R indices (all data)	R1 = 0.0388, wR2 = 0.1129	
Absolute structure parameter	–0.07(6)	

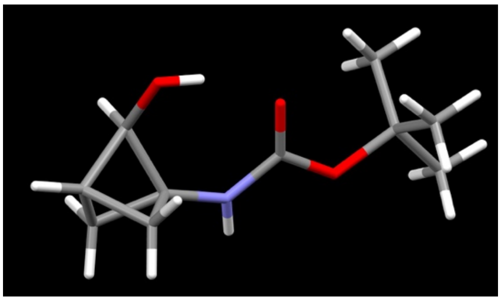

Table S8.8: Crystal data and structure refinement for (±)-**28**

CCDC code	2208871	
Empirical formula	$C_{10}H_{15}NO_3$	
Formula weight	197.23	
Temperature	150 K	
Wavelength	1.54184 Å	
Crystal system / Space group	Orthorhombic	P n a 2_1
Unit cell dimensions	a = 9.5843(3) Å	α = 90°
	b = 6.42090(10) Å	β = 90°
	c = 16.9036(4) Å	γ = 90°
Volume	1040.24(4) $Å^3$	
Z	4	
Density (calculated)	1.259 Mg/m^3	
Crystal size	0.25 × 0.12 × 0.05 mm^3	
Independent reflections	1862 [R(int) = 0.032]	
Completeness to theta = 74.661°	99.6 %	
Absorption correction	Semi-empirical from equivalents	
Refinement method	Full-matrix least-squares on F^2	
Data / restraints / parameters	1862 / 13 / 138	
Goodness-of-fit on F^2	1.0042	
Final R indices [I>2sigma(I)]	R1 = 0.0316, wR2 = 0.0839	
R indices (all data)	R1 = 0.0321, wR2 = 0.0849	

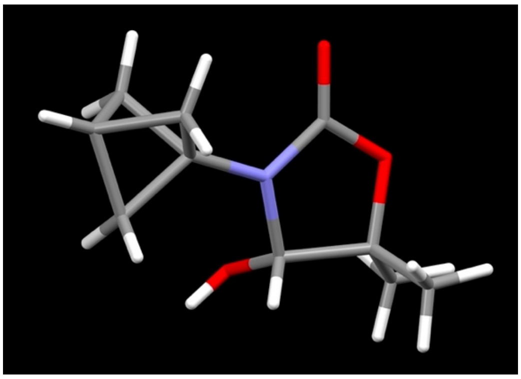

S9 References

(1) Whitehouse, C. J. C.; Bell, S. G.; Tufton, H. G.; Kenny, R. J.; Ogilvie, L. C.; Wong, L. L., *Chem. Commun.* **2008**, *44*, 966–968.

(2) (a) Whitehouse, C. J. C.; Yang, W.; Yorke, J. A.; Rowlatt, B. C.; Strong, A. J.; Blanford, C. F.; Bartlam, M.; Wong, L. L.; Rao, Z., *Chembiochem*, **2010**, *11*, 2549–2556; (b) Whitehouse, C. J. C.; Yang, W.; Yorke, J. A.; Tufton, H. G.; Ogilvie, L. C.; Bell, S. G.; Zhou, W.; Bartlam, M.; Rao, Z., Wong, L. L., *Dalton Trans.* **2011**, *40*, 10383–10396; (c) Ren, X.; Yorke, J. A.; Taylor, E.; Zhang, T.; Zhou, W.; Wong, L. L., *Chem. Eur. J.* **2015**, *21*, 15039–15047.

(3) Omura, T.; Sato, R., *J. Biol. Chem.* **1964**, *239*, 2370–2378.

(4) Li, P.; Ma, N.; Wang, Z.; Dai, Q.; Hu, C. *J. Org. Chem.* **2018**, *83*, 8233−8240.

(5) Zhou, S.; Lv, K.; Fu, R.; Zhu, C.; Bao, X. *ACS Catal.* **2021**, *11*, 5026–5034.

(6) Bunker, K. D.; Guo, C.; Grier, M. C.; Hopkins, C. D.; Pinchman, J. R.; Slee, D. H.; Huang, Q.; Kahraman, M. US Patent 2016/0075654 A1, Mar 17, 2016

(7) Chernykh, A. V.; Kudryk, O. V.; Olifir, O. S.; Dobrydnev, A. V.; Rusanov, E.; Moskvina, V. S.; Volochnyuk, D. M.; Grygorenko, O. O. *J. Org. Chem.* **2023**, *88*, 3109–3131.

(8) Radchenko, D. S.; Pavlenko, S. O.; Grygorenko, O. O.; Volochnyuk, D, M.; Shishkina, S. V.; Shishkin, O. V.; Komarov, I. V. *J. Org. Chem.* **2010**, *75*, 5941–5952.

(9) Jiang, C.; Chen, R.; Pandey, A.; Kalita, B.; Duraiswamy, A. J. US Patent 2019/0263802 A1, Aug 29, 2019.

(10) Hoye, T. R.; Jeffrey, C. S.; Shao, F. *Nat. Protoc.* **2007**, *2*, 2451–2458.

(11) Cosier, J.; Glazer, A. M. *J. Appl. Crystallogr.* **1986**, *19*, 105–107.

(12) Palatinus, L.; Chapuis, G. *J. Appl. Crystallogr.* **2007**, *40*, 786–790.

(13) (a) Betteridge, P. W.; Carruthers, J. R.; Cooper, R. I.; Prout, K.; Watkin, D. J. *J. Appl. Crystallogr.* **2003**, *36*, 1487–1487; (b) Parois, P.; Cooper, R. I.; Thompson, A. L. *Chem. Cent. J.* **2015**, *9*, 30.

(14) Sheldrick, G. M. *Acta Cryst.* **2008**, *A64*, 112–122.

(15) Sheldrick, G. M. Acta Cryst. **2015**, C71, 3–8.

(16) Cromer, D.T. and J.T. Waber, International Tables for X-ray Crystallography, Vol. 4, Table 2.2 A. 1974, Kynoch Press: Birmingham, UK.
